# The role of recessive inheritance in early-onset epileptic encephalopathies: a combined whole-exome sequencing and copy number study

**DOI:** 10.1038/s41431-018-0299-8

**Published:** 2018-12-14

**Authors:** Sorina M. Papuc, Lucia Abela, Katharina Steindl, Anaïs Begemann, Thomas L. Simmons, Bernhard Schmitt, Markus Zweier, Beatrice Oneda, Eileen Socher, Lisa M. Crowther, Gabriele Wohlrab, Laura Gogoll, Martin Poms, Michelle Seiler, Michael Papik, Rosa Baldinger, Alessandra Baumer, Reza Asadollahi, Judith Kroell-Seger, Regula Schmid, Tobias Iff, Thomas Schmitt-Mechelke, Karoline Otten, Annette Hackenberg, Marie-Claude Addor, Andrea Klein, Silvia Azzarello-Burri, Heinrich Sticht, Pascal Joset, Barbara Plecko, Anita Rauch

**Affiliations:** 1 Institute of Medical Genetics, University of Zurich, Schlieren-Zurich, 8952 Switzerland; 2 Victor Babes National Institute of Pathology, Bucharest, 050096 Romania; 3 Division of Child Neurology, University Children’s Hospital Zurich, Zurich, 8032 Switzerland; 4 CRC Clinical Research Center University, Children’s Hospital Zurich, Zurich, 8032 Switzerland; 5 radiz—Rare Disease Initiative Zürich, Clinical Research Priority Program for Rare Diseases University of Zurich, Zurich, 8032 Switzerland; 6 Institute of Biochemistry, Friedrich-Alexander-Universität Erlangen-Nürnberg (FAU), Erlangen, 91054 Germany; 7 Pediatric Emergency Department, University Children’s Hospital Zurich, Zurich, 8032 Switzerland; 8 Children’s department, Swiss Epilepsy Centre, Clinic Lengg, Zurich, 8000 Switzerland; 9 Division of Child Neurology, Kantonsspital Winterthur, Winterthur, 8401 Switzerland; 10 Municipal Hospital of Zurich Triemli, Zurich, 8063 Switzerland; 11 Division of Child Neurology, Children’s Hospital, Lucerne, 6000 Switzerland; 12 Department of Woman-Mother-Child, University Medical Center CHUV, Lausanne, 1015 Switzerland; 13 Division of Paediatric Neurology, University Childerns Hospital Basel, UKBB, 4031 Basel, Switzerland; 14 Division of Paediatric Neurology, Development and Rehabilitation, University Children’s Hospital, 3010 Bern, Switzerland; 15 Neuroscience Center Zurich, University of Zurich and ETH Zurich, Zurich, 8057 Switzerland; 16 Division of General Pediatrics, Department of Pediatrics, Medical University of Graz, 8036 Graz, Austria; 17 Zurich Center for Integrative Human Physiology, University of Zurich, Zurich, 8057 Switzerland

**Keywords:** Neurodevelopmental disorders, Medical genetics, Genetics research, Genetic testing, Paediatrics

## Abstract

Early-onset epileptic encephalopathy (EE) and combined developmental and epileptic encephalopathies (DEE) are clinically and genetically heterogeneous severely devastating conditions. Recent studies emphasized de novo variants as major underlying cause suggesting a generally low-recurrence risk. In order to better understand the full genetic landscape of EE and DEE, we performed high-resolution chromosomal microarray analysis in combination with whole-exome sequencing in 63 deeply phenotyped independent patients. After bioinformatic filtering for rare variants, diagnostic yield was improved for recessive disorders by manual data curation as well as molecular modeling of missense variants and untargeted plasma-metabolomics in selected patients. In total, we yielded a diagnosis in ∼42% of cases with causative copy number variants in 6 patients (∼10%) and causative sequence variants in 16 established disease genes in 20 patients (∼32%), including compound heterozygosity for causative sequence and copy number variants in one patient. In total, 38% of diagnosed cases were caused by recessive genes, of which two cases escaped automatic calling due to one allele occurring de novo. Notably, we found the recessive gene *SPATA5* causative in as much as 3% of our cohort, indicating that it may have been underdiagnosed in previous studies. We further support candidacy for neurodevelopmental disorders of four previously described genes (*PIK3AP1*, *GTF3C3, UFC1*, and *WRAP53*), three of which also followed a recessive inheritance pattern. Our results therefore confirm the importance of de novo causative gene variants in EE/DEE, but additionally illustrate the major role of mostly compound heterozygous or hemizygous recessive inheritance and consequently high-recurrence risk.

## Introduction

The term epileptic encephalopathy (EE) denotes a rare group of severe, early-onset epilepsies that tend to be pharmacoresistant with generally poor outcome and often severe intellectual disability [1]. EEs are heterogeneous with respect to seizure semiology and neurological comorbidities and encompass several clinical entities, such as Dravet- (MIM 607208), Ohtahara- (MIM 308350), and West syndromes (MIM 308350). They have been long thought to be acquired rather than genetic, given their common occurrence in a single individual with no family history [2]. Advances in genomic technologies over the past decade have unraveled a variety of genetic causes of epileptic encephalopathies and indicated that in some of these genetic entities intellectual disability or autism may be a primary clinical sign independent of the presence of epilepsy [1, 2]. These observations resulted in the new recommendation in 2017 to refer to EE only where “the abundant epileptiform activity interferes with development resulting in cognitive slowing and often regression” and “developmental and epileptic encephalopathy” (DEE) where there is additional developmental impairment independent of the epileptic activity [3].

Despite the growing number of known EE/DEE disease genes, the majority of patients still remain without etiological diagnosis. A retrospective, single-center cohort study evidenced that clinical signs, MRI, and targeted metabolic tests lead to an etiological diagnosis in about 13% of patients with “epileptic encephalopathy” [4]. In patients with a clinically unrecognized etiology (likely) causative de novo copy number variants (CNVs) accounted for about 3–10% of patients in several studies [4–8]. A large whole-exome sequencing study in “epileptic encephalopathy” patients established de novo variants, as disease cause in 12% [9]. This finding was further supported by gene panel and whole-genome sequencing studies which, considering those with a minimum of 50 patients, revealed a diagnostic yield of up to 37 and 32%, respectively, again with the vast majority of disease-associated variants occurring as de novo events [4, 10–17]. However, a recent report on diagnostic exome sequencing in 89 unselected patients with “epileptic encephalopathy” yielded a diagnosis in ∼43% cases, including ∼10% with recessive inheritance [18]. In order to further evaluate the etiology and the role of recessive inheritance in EE/DEE, we performed a comprehensive single-center study to unravel the underlying etiology in 63 deeply phenotyped patients with early-onset EE or DEE of clinically unrecognized etiology by combining whole-exome sequencing and high-resolution chromosomal CNV studies.

## Subjects and methods

### Patients

After approval of this single-center study by the ethical committee of the Kanton of Zurich and following informed consent by the participating individuals or their legal guardians, patients, available parents, and selected further family members were enrolled from 2013–2015 if the patients fulfilled the following inclusion criteria: (1) developmental delay and onset of epilepsy below the age of 4.5 years (in order to encompass early-infantile, infantile, and early childhood-onset epilepsy), (2) pharmacoresistance for at least 6 months (reflecting the common definition of pharmacoresistance as ongoing seizures after trial of two different drugs and the time needed to try two antiepileptics), (3) no persistent spike wave focus in EEG (because they may indicate localized cortical dysplasia which is not yet detectable on early MR images as a distinct etiological clue), (4) absence of specific malformations on cerebral MRI, and (5) unknown etiology after standard clinical evaluation including an extended targeted metabolic screening (B6 vitamers, amino acids, and pipecolic acid in the plasma, alpha-aminoadipic semialdehyde and organic acids in urine). All patients were seen by experienced neuropediatricians and medical geneticists and deeply phenotyped including clinical and family history, physical examination and analysis of seizure semiology, and EEG findings.

### Chromosomal microarray analysis (CMA)

After extraction of genomic DNA from peripheral blood samples, high-resolution chromosomal microarray studies were performed using Affymetrix Cytoscan HD arrays (containing about 750,000 genotype-able SNPs and 1.9 million non-polymorphic probes) (Affymetrix, Santa Clara, CA, USA), as previously described [19]. The analyses were primarily performed in patients only, followed by parental testing in case of (likely) causative or unclear findings in the patient. Rare CNVs not present in in-house and Affymetrix healthy control samples as well as CNVs overlapping recurrent disease loci underwent visual assessment of the actual marker location and distribution, and these were further investigated for their clinical relevance using the following databases: DECIPHER (https://decipher.sanger.ac.uk/), ISCA consortium (https://www.iscaconsortium.org/), OMIM (Online Mendelian Inheritance in Man, https://www.omim.org/), UCSC genome browser (http://genome.ucsc.edu/), and PubMed (http://www.ncbi.nlm.nih.gov/pubmed). De novo CNVs were confirmed by alternative methods when their confidence score or marker content did not allow reliable calling of the CNV by array data alone. All detected rare CNVs were submitted to the DECIPHER database [20].

### Whole-exome sequencing and further studies

Whole-exome sequencing was performed using the Agilent SureSelectXT Clinical Research Exome Kit (V5 CRE) (Agilent Technologies, Santa Clara, CA, USA) for capturing, followed by paired-end sequencing of 125 bp forward and 125 bp reverse using a HiSeq SBS Kit v4 on a HiSeq2500 System (Illumina, San Diego, CA, USA). To achieve a high average coverage, only 12–15 samples were pooled within four lanes. Raw fastQ files were aligned to the hg19 reference genome using the NextGENe software (SoftGenetics, State College, PA, USA). Average sequencing coverage was 212-fold with > 96% of the region-of-interest covered at least 20-fold. Variants were called if observed in at least 16% of reads with sufficient quality level. The resulting variants were annotated for potential damaging effects by standard in silico prediction tools, such as SIFT (http://sift.jcvi.org/), PolyPhen2 (http://genetics.bwh.harvard.edu/pph2/), LRT, MutationTaster (http://www.mutationtaster.org), MutationAssessor, FATHMM, GERP, and CADD (http://cadd.gs.washington.edu/), as provided within the NextGENe software. The ALAMUT visual prediction software version 2.7.2 (Interactive Biosoftware, Rouen, France) was used for additional exploration in selected variants. Rare protein changing variants and splice site variants (including 12 intronic basepairs) with an overall minor allele frequency below 2% were analyzed for de novo, compound heterozygous, and homozygous calls, and additionally for inherited X-chromosomal calls in boys, and visually inspected for verification of the assumed inheritance pattern. Resulting genes with potentially damaging variants were manually assessed for known or suspected association with EE or overlapping neurodevelopmental disorders using a variety of databases, such as ClinVar, DECIPHER, Exome Aggregation Consortium (ExAC, http://exac.broadinstitute.org/), HGMD (Human Genome Mutation Database, http://www.hgmd.org/), ISCA consortium, OMIM, UCSC genome browser, PubMed, Simons Foundation SFARI database (https://gene.sfari.org/), and SysID database (http://sysid.cmbi.umcn.nl/). All de novo variants, diagnostic and candidate disease variants were confirmed by Sanger sequencing and discussed within the team, including natural scientists, clinical geneticists, and neuropediatricians. In selected candidate missense variants, potential functional effects were further evaluated by structural mutation modeling. In patients with unavailability of one parent, in-silico designed filters for genes known to be associated with intellectual disability or epilepsy were used. Independently, all variants were filtered for overlap with specific variants described in the Human Genome Mutation Database, for inherited variants in known intellectual disability/epilepsy genes unreported at the time of analysis, and for loss-of-function variants in genes of the Human Genome Mutation Database. Classification of potentially deleterious variants in known disease genes followed the ACMG guidelines [21]. From the ExAC database, only variants that passed the quality filters were considered. In selected cases with only one disease-associated sequence or copy number variant in recessive disease genes, sequencing gaps were covered by Sanger sequencing. In selected cases with X-chromosomal variants potentially affecting function, X-inactivation ratios were tested as described by Lau et al. [22]. In selected cases with variants of unclear significance in splice sites or with monoallelic disease-associated variants in recessive disease genes RNA was extracted from blood samples using the PAXgene Blood System (PreAnalytiX, Hombrechtikon, Switzerland) and transcribed into cDNA using Superscript III Reverse Transcriptase and random hexamers (Invitrogen/ThermoFisher Scientific, Waltham, MA, USA) and used for PCR amplifications and Sanger sequencing, respectively. All EE-associated and candidate variants were submitted to the LOVD [23] (Leiden Open Variation Database) accessible at www.LOVD.nl/ followed by the respective gene name, e.g., www.LOVD.nl/SPATA5 database. The respective LOVD patient IDs are given in Table 3 and the Supplementary Tables.

In plasma samples of three patients with findings from exome sequencing that indicated disturbance of metabolic pathways, we performed an untargeted metabolomic screen as previously described [24, 25].

## Results

### Patient characteristics

We recruited a total of 63 independent, mostly sporadic index cases with EE (*n* = 42; 67%) or DEE (*n* = 21; 33%). Five (8%) had affected siblings: two had a likewise affected monozygotic twin, and three had one or two likewise affected siblings. Parents of four index cases (6%) were consanguineous (three sporadic and one patient with affected siblings). In all but two families in whom the fathers were not available, DNA samples of both parents could be collected. Index cases comprised 38 males and 25 females. The median age of seizure onset was 7 months (range 1 month to 4 3/12 years). The median age at last investigation of index patients was 7 years (range 6 months to 38 years). Four patients (6%) deceased during the study. The 58 patients for whom detailed information was available took on average 5.6 different antiepileptic drugs over the course of their disease. Neurodevelopmental characteristics of the cohort are summarized in Table 1 and detailed phenotypic information is provided in Table S1 in the Supplementary Material.

**Table 1:** Summary of main clinical characteristics in 63 index patients

Neurodevelopmental traits	Number of patients (percentage)
Profound intellectual disability	16 (25.3%)
Moderate to severe intellectual disability	47 (74.6%)
Ataxia	21 (33.3%) (44% of those who could walk)
Cerebral palsy	31 (49.2%)
Muscular hypotonia	49 (77.8%)
Microcephaly	27 (41.9%)
Macrocephaly	4 (6.3%)
Single seizure type	6 (9.5%)
Multiple seizure types	57 (90.5%)
Status epilepticus	13 (20.6%)
*Seizure Semiology*	
Epileptic spasms	20 (31.8%)
Generalized/focal tonic seizures	36 (57%)
Generalized/focal clonic seizures	10 (15.9%)
Generalized tonic-clonic seizures	33 (52.4%)
Generalized/focal myoclonic seizures	31 (49.2%)
Atonic seizures	15 (42.9%)
Myoclonic-atonic seizures	5 (7.9%)
Focal seizures aware	19 (30.2%)
Focal seizures with impaired awareness	17 (26.9%)
Early-onset absences	10 (15.9%)
Atypical absences	7 (11.1%)

### Genomic analyses

**Fig. 1:**
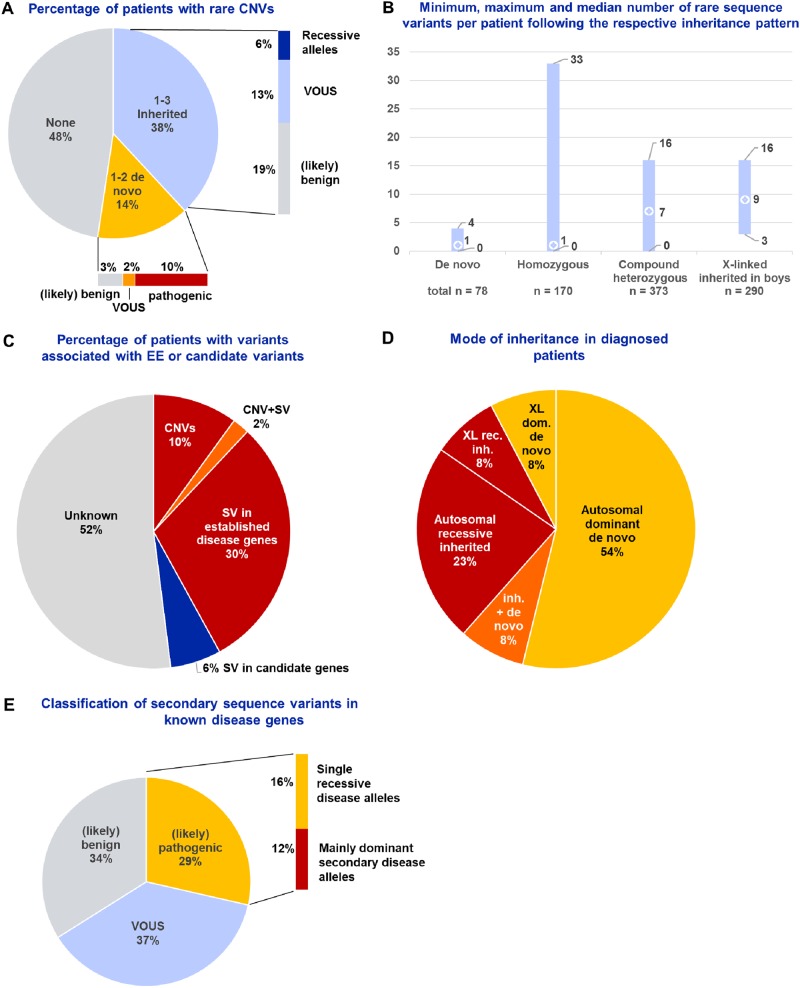
Summary of genetic findings. **a** Percentage of patients with ≥ 1 de novo or inherited rare copy number variants and their respective classification of clinical relevance. **b** Total numbers, and medians, minimum and maximum numbers per patients of variants following the respective modes of inheritance identified by whole-exome sequencing in 51 child–parent trios. **c** Percentage of patients with (likely) disease-associated CNV or SV findings in established EE genes or with potentially disease-causing candidate genes. **d** Distribution of inheritance modes in diagnosed patients with (likely) EE-associated CNV or SV in established disease genes. **e** Results of (re-) classification of 56 secondary findings from whole-exome sequencing, of which 16% represented carriership of single alleles of recessive disorders and 12% indicated mainly dominant disease alleles as secondary findings. CNV copy number variant, SV sequence variant, VOUS  variants of unknown clinical significance, XL dom. X-linked dominant, XL rec. inh. X-linked recessive inherited

Chromosomal microarray analysis revealed a total of 50 reliably called rare coding and recurrent disease loci CNVs in 33 (52%) of index patients sizing 7–7700 kb with a median size of 110 kb and a maximum number of three per patient (Table S2 in the Supplementary Material). Six of the nine de novo CNVs affected well-known disease loci (∼10% of the cohort) (Fig. 1a and Tables 2–3 and Tables S2 and S3 in the Supplementary Material). Five of the latter established a diagnosis unequivocally explaining the EE/DEE phenotype, but we cannot exclude further contributing factors to pathogenicity in the de novo 2 Mb duplication of the chromosomal region 22q11.22–22q11.23 (MIM 608363) representing a recurrent CNV between the low copy repeats LCR22-E and LCR22-H causing a variable neurodevelopmental phenotype with reduced penetrance [26]. Four of the inherited heterozygous deletions affected known recessive disease genes, of which two, *FARS2* (MIM 611592) and *SPATA5* (MIM 613940), have been associated with neurodevelopmental disorders. Notably, one male and two female patients (5%) without definite diagnosis harbored the recurrent Xp22.31 duplication or triplication of 1.7 Mb including the *STS* (MIM 300747) locus, which has been discussed as a risk factor for neurodevelopmental disease and epilepsy in both sexes [27–29]. In our Affymetrix European and American healthy control cohort, the duplication was found in three females out of 1038 individuals indicating a population prevalence of 0.3%, which is similar to that found in a previous study [29]. While the latter study found no significant difference between 20,095 various clinical cases and 5088 controls, the frequency of copy number gains at the *STS* locus was significantly increased in our epileptic encephalopathy cohort (*p* < 0.0001, Chi-Square two-sided test). We therefore analyzed further available members in two families, which indicated no segregation of the duplication with the phenotype, but left the clinical relevance of the triplication open (Supplemental Figure S1 and Table S2 in the Supplementary Material).

**Table 2:** Summary of established genes causing EE/DEE in our study cohort, as well as recurrent candidate genes for neurodevelopmental disorders

Disease gene category	Mode of inheritance			
	Autosomal		X-linked	
	Dominant	Recessive	Dominant	Recessive
	(*de novo*)	(inherited or otherwise indicated)	(*de novo*)	(inherited)
Established genes/regions	1p36 deletion	*ACO2* CH	*CDKL5* deletion	*ARX*
	22q11.2 duplication	*AP4S1* HO	*CDKL5*	*SMS*
	*KCNQ2*	*BRAT1* CH		
	*MBD5* deletion	*POLG* CH		
	*SCN1A* (3 × )	*SPATA5* CH (one allele deletion)		
	*SCN2A*	*SPATA5* CH (*de novo* and inherited)		
	*SCN8A*	*SZT2* CH (*de novo* and inherited)		
	*STXBP1* (2 × )	*PRUNE1* HO		
	*UBE3A* deletion (2 × )			
	*GABRB2*			
Recurrent candidate genes	*PIK3AP1*	*GTF3C3* CH		
		*UFC1* HO		
		*WRAP53* CH		

*CH* compound heterozygous, *HO* homozygous

**Table 3:** Main clinical features in diagnosed cases

Patient ID LOVD or Decipher ID	sex	(Likely) pathogenic sequence or copy number variant gene / region (inheritance) reference transcript (hg19)	Incidental findings	Ethnicity	Parental consanguinity	Age at last evaluation	Age at seizure onset	Type of encephalopathy and seizures	DD/ID	MRI findings	Hypotonia	Spasticity	Ataxia	Microcephaly
72356 Decipher 370071	f	**1p36.23-pter (AD, DN)** deletion (7.7 Mb) chr1:g.(0_849466)_(7666975_7683885)del	**-**	Caucasian	No	12 m	Neonatal	EE; Apneas, focal myoclonic (periorbital), My, T, C	Moderate-to-severe	Normal	+	-	-	+
71592 Decipher 370076	m	**22q11.22 -q11.23 (AD, DN)** recurrent microduplication chr22:g.(22953405_22953514)_(25026857_25027086)dup	**RAF1? (AD)**	Caucasian	No	16 y 2 m	FS at 2 y, seizures at 3 y 3 m	EE; FS, ES, myoclonic-atonic, A, C	Severe	Normal	+	-	+	+
69986 LOVD 176985	f	**ACO2 (AR, CH)** NM_001098.2:c.[1859G > A];[2048 G > T] p.[(Gly620Asp)];[(Gly683Val)]	**-**	Caucasian	No	1 y 10 m	7 m	EE; Generalized myoclonic, T, focal impaired awareness, SE	Severe	Marked cerebral and cerebellar atrophy, diffuse leukencephalopathy	+	+	-	+
70757 LOVD 176997	f	**AP4S1 (AR, HO)** NG_031913.1(NM_007077.4):c.[138 + 3_138+6del];[138+3_138+6del] p.[?];[?]	**-**	Caucasian	No	1 y 3 m	Neonatal	EE; T, focal aware, focal impaired awareness	Moderate	Aqueductal stenosis with hydrocephalus internus	+	+	+	-
72892 LOVD 177002	m	**ARX (XL-R, inherited)** NM_139058.2:c.1057 C > T p.(Pro353Ser)	**-**	Afghan	No	6 m	8w	EE; ES, C	Severe	Normal	+	+	-	-
73311 LOVD 177003	m	**BRAT1 (AR, CH)** NM_152743.3:c.[2125_2128del];[638dup]p.[(Phe709Thrfs*17)];[(Val214Glyfs*189)]	**-**	Caucasian	No	6 m	Neonatal	EE; My	Severe	NA	-	+	-	-
72128 Decipher 370072	f	**CDKL5 (XL-D, DN)** mosaic deletion (25 Kb) chrX:g.(18592712_18592741)_(18617503_18617862)del	**-**	Caucasian	No	11 m	6w	EE; ES, T, C, eye lid myoclonia	Moderate	Normal	+	-	-	+
72404 LOVD 177004	m	**CDKL5 (XL-D, DN)** NG_008475.1(NM_003159.2):c.282+3_282+6del	**-**	Caucasian	No	8 y 4 m	1 m	EE; T, GTC	Severe	Normal	+	+	-	-
73324 LOVD 177005	f	**GABRB2 (AD, DN)** NM_021911.2:c.719 G > C p.(Arg240Thr)	**-**	Caucasian	No	6 y 6 m	2 y 4 m	DEE; GTC, T, My, AA, subclinical SE	Severe	Normal	+	-	+	+
73214 LOVD 176996	f	**KCNQ2 (AD, DN)** NM_172107.2:c.740 C > T p.(Ser247Leu)	**-**	Caucasian	No	3y4m	Neonatal	EE; T, My	Severe	Normal	+	-	-	+
50126 Decipher 370070	m	**MBD5 (AD, DN)** deletion (197 Kb) chr2:g.(148757084_148762374)_(148959158_ 148960882)del	**-**	Caucasian	No	13 y 5 m	1 y 7 m	EE; FS (first seizures), GTC, T	Severe	Multiple T2 hyperintensities, multiple parenchymal lesions	-	+	+	+
71693 LOVD 177006	m	**POLG (AR, CH)** NM_002693.2:c.[2542 G > A];[824 G > A]p.[(Gly848Ser)];[(Arg275Gln)]	**-**	Caucasian	No	14 y 7 m	1 y 6 m	DEE; A, eyelid myoclonia, focal clonic (Epilepsia partialis continua)	Moderate	Low-grade glioma in the left thalamus, diffusion restriction in the left occipital region	+	-	+	+
69937 LOVD 177008	m	**PRUNE1 (AR, HO)** NM_021222.2:c.[316 G > A];[316 G > A]p.[(Asp106Asn)];[(Asp106Asn)]	**-**	Sri Lankan	1st degree cousins	1 y 2 m	6 m	DEE; ES, focal aware, T, My	Severe	Immature cortex differentiation (35 GW), punctate cerebellar hemorrhages, signs of hypoxia	+	+	-	+
34124 LOVD 177009	m	**SCN1A (AD, DN)** NM_001165963.1:c.5348 C > T p.(Ala1783Val)	-	Caucasian	No	16 y	4.5 m	EE; Focal clonic (sec gen), GTC with cyanosis, My, AB, recurrent SE, infection-triggered seizures	Severe	Cerebral atrophy, enlargement of Virchow Robin spaces	+	-	+	-
47970 LOVD 177010	m	**SCN1A (AD, DN)** NM_001165963.1:c.4754del p.(Thr1585Metfs*6)	**-**	Caucasian	No	17 y 6 m	2 m	EE; Focal, GTC, SE (infection-triggered), AB	Severe	Normal	-	+	-	-
75143 LOVD 177011	f	**SCN1A (AD, DN)** NM_001165963.1:c.1142del p.(Gln381Argfs*10)	**-**	Caucasian	No	28 y	3 m	EE; GTC, T, focal impaired awareness, reflex seizures (photosensitivity)	Severe	Cerebellar atrophy	-	+	-	NA
42680 LOVD 177012	m	**SCN2A (AD, DN)** NM_021007.2:c.5408 A > G p.(Glu1803Gly)	**-**	Caucasian	No	14 y	Neonatal	EE; T (serial), My, apneas	Severe	Generalized atrophy, hippocampal atrophy and sclerosis, atrophy of corpus callosum	+	+	-	-
43092 LOVD: 177013	m	**SCN8A (AD, DN)** NM_014191.3:c.5615 G > A p.(Arg1872Gln)	**CHST6 (carrier)**	Caucasian	No	13 y 7 m	3 m	EE; GTC, T, focal sec gen seizures with apneas and cyanosis	severe	Cerebral atrophy	+	-	+	-
72555 LOVD 177014	m	**SMS (XL-R, inherited)** NM_004595.4:c.388 C > T p.(Arg130Cys)	**FIG4 (carrier)**	Caucasian	No	2 y 11 m	12 m	DEE; ES, T, A, AA	Severe	Hypoplasia of corpus callosum, slight cerebral cortical atrophy	+	+	-	+
47651 Decipher 370082 LOVD 177015	m	**SPATA5 (AR, CH)** chr4:g.[(123951799_123952079)_(124003383_124003384)del];[123855735_123855737del] NM_145207.2:c.[2080_2213del];[989_991del] p.[(Gly694Phefs*23)];[(Thr330del)]	**-**	Caucasian	No	13 y	8 m	DEE; ES, T, C, A, AB, gelastic seizures	Severe	Delayed myelination, cortical atrophy, white matter atrophy, thin corpus callosum	-	+	-	-
73068 LOVD 177016	m	**SPATA5 (AR, CH, DN on one allele)** NM_145207.2:c.[2389 C > G];[1 A > C]p.[(Pro797Ala)];[(Met1?)]	**-**	Caucasian	No	5 y 7 m	5 m	EE; ES, GTC, T, focal aware	Severe	Delayed myelination, supra- and infratentorial white matter atrophy	-	+	-	+
52236 LOVD 00181099	f	**STXBP1 (AD, DN)** chr9:130422393 NG_016623.1(NM_003165.3):c.325+6 T > C	**-**	Caucasian	No	10 y 2 m	1 m	EE; Focal impaired awareness, eyelid myoclonia	Severe	Normal	+	-	+	-
73805 LOVD 177023	m	**STXBP1 (AD, DN)** NM_003165.3:c.1268 T > C p.(Leu423Pro)	**-**	Caucasian	No	7 m	Neonatal	EE; T, C, My, ES, fever- and infection triggered seizures	Moderate	Bilateral hyperintense ischemic lesion in the posterior limb of the capsula interna	+	+	-	-
72943 LOVD 177024	m	**SZT2 (AR, CH?, DN on one allele)** NM_015284.3:c.1045del(;)1891G > Ap.(Ser349Profs*9)(;)(Glu631Lys)	**-**	Caucasian	No	1 y 8 m	6 m	EE; ES, GTC, focal impaired awareness	Severe	Dysmorphic corpus callosum, septations in the frontal ventricles, polymicrogyria	+	-	-	-
71412 Decipher 370084	f	**UBE3A (AD, DN)** 15q11.2-q13.11 deletion (4.9 Mb) chr15:g.(23620154_23620191)_(28545355_28545445)del	**OTOF (carrier)**	Caucasian	No	2 y 5 m	1 y	DEE; AB	Severe	NA	+	-	+	+
49635 Decipher 370081	m	**UBE3A (AD, DN)** deletion (134 Kb) chr15:g.(25583244_25583408)_(25717757_25717851)del	**-**	Caucasian	No	12 y 10 m	FS at 3 y 10 m, seizures at 6 y 4 m	DEE; FS, GTC, focal impaired awareness	Severe	Normal	-	-	+	-

*A* atonic, *AA* atypical absences, *AB* absences, *AD* autosomal dominant, *AR* autosomal recessive, *C* clonic, *CH* compound heterozygous, *DEE* developmental and epileptic encephalopathy, *DN* de novo, *EE* epileptic encephalopathy, *ES* epileptic spasms, *F* female, *FS* febrile seizures, *GTC* generalized tonic-clonic, *HO* homozygous, *M* male, *m* months, *My* myoclonic, *NA* not available, *SE* status epilepticus, *sec gen* secondarily generalized, *T* Tonic, *w* weeks, *XL-D* X-linked dominant, *XL-R* X-linked recessive, *y* years

Whole-exome sequencing was performed in 60 patients, one sibling and 104 parents. Three of the five patients with a de novo CNV fully explaining the phenotype were not exome sequenced. In nine patients, only the index cases were exome sequenced and since a final diagnosis could be already reached in eight of these, parental samples were only used for segregation analysis by Sanger sequencing. Since only one patient in whom trio-sequencing was not possible due to lack of parental samples remained without diagnosis, this approach did not affect diagnostic yield statistics. In the 51 trios who underwent whole-exome sequencing in the patients and both parents we identified per patient a median of one de novo, one homozygous, seven compound heterozygous, and nine X-linked inherited rare variants (Fig. 1b). In 20 patients, disease-associated variants in established EE/DEE genes were identified establishing an etiological diagnosis in 32% of the total cohort (Fig. 1c, d, Table 3, Tables S3 and S4 in the Supplementary Material). These sequence alterations include 15 novel variants, first described in this paper, and occurred in 14 well-known EE/DEE genes as well as in two disease genes (*GABRB2* [MIM 600232], *PRUNE1* [MIM 617413]) previously reported in a few patients only (Table 2 and S3). Very recently, Hamdan et al. [16] reported 11 patients harboring de novo missense variants in *GABRB2* with early-onset epilepsy, severe intellectual disability, acquired microcephaly, hypotonia, and further neurological signs in the majority of patients. Our patient (ID 73324) harboring a novel de novo variant c.719 G > C p.(Arg240Thr) in *GABRB2* likely affecting function also shows these common features (see Table S1 and S3 in the Supplementary Material).

*PRUNE1* was recently described to cause a novel autosomal recessive disease (MIM 617481) characterized by severe global developmental delay, microcephaly, dysmorphic features, muscle tone abnormalities, and variable brain abnormalities such as delayed myelination, thin corpus callosum or cerebellar hypoplasia, but several patients are also reported to have early-onset seizures (see also Supplementary Table S8) [30]. Our index patient (ID 69937) from consanguineous parents had a severe DEE phenotype but cerebral MRI showed no malformation but rather immature cortex differentiation and discrete signs of hypoxia. He had two brothers with a similar phenotype that died at the age of 4 m and 2 y 9 m, respectively (see Table S1 in the Supplementary Material). The homozygous variant in *PRUNE1* (NM_021222.2:c.[316 G > A];[316 G > A] p.[(Asp106Asn)];[(Asp106Asn)]), details in Table S3 in the Supplementary Material) detected in our patient and his available affected brother is the same sequence variant that was already reported as disease-associated in two families by Karaca et al. [30].

In two index patients and one affected brother with variants of unclear significance in established disease genes affecting metabolic pathways, we were able to confirm pathogenicity by untargeted metabolomic analyses. In monozygotic twin brothers (ID 72555 and 72719) carrying a missense variant in the X-linked spermine synthase gene (*SMS*, MIM 300105) related to Snyder–Robinson syndrome, we found significantly increased N8-acetylspermidine in plasma, a metabolite that accumulates upstream to the enzyme defect [25]. In a female patient (ID 69986) with two novel inherited compound, heterozygous variants in the aconitase 2 gene (*ACO2*, MIM 100850) plasma metabolomic analysis distinguished a metabolic fingerprint profile that reflects the enzyme deficiency within the citric acid cycle but also perturbations in associated metabolic pathways [24].

Additionally, we searched for secondary findings in the exome sequencing data and hereby identified 56 specifically reported disease alleles or loss-of-function or unreported variants in *Human Genome Mutation Database* genes not related to EE and reclassified them manually (Fig. 1e and Table 3 and S7 of the Supplementary Material). An overview of primary and secondary findings, as well as variants in candidate genes is provided in Table S8 of the Supplementary Material.

## Discussion

**Fig. 2:**
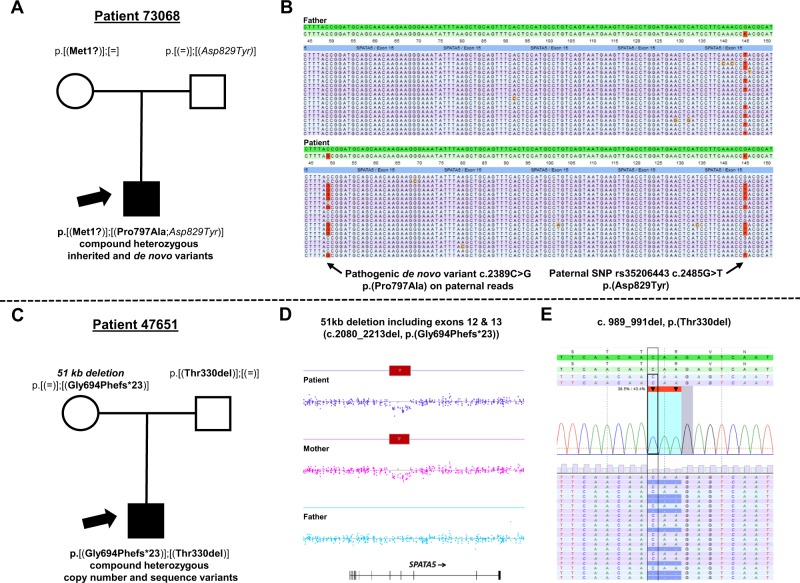
Disease causing biallelic *SPATA5* variants in two independent families demonstrating the value of hypothesis driven manual data analysis (variant nomenclature and exon numbering according to NM_145207.2 and NG_051570.1, respectively). **a**, **b** Pedigree of patient 73068 (**a**) summarizing the segregation of the maternally inherited variant p.(Met1?) and the *de novo* variant p.(Pro797Ala) on the paternal allele; (**b**) Whole-exome sequencing data of exon 15 in the patient demonstrating the localization of both, the de novo variant c.2389 C > G (p.(Pro797Ala)) and the paternally inherited single nucleotide polymorphism (SNP) rs35206443 (c.2485 G > T, p.(Asp829Tyr)) on the same allele. **c**-**e** Pedigree of patient 47651 illustrating the compound heterozygous constellation of the maternally inherited, heterozygous 51 kb deletion comprising exon 12 and 13 (c.2080_2213del; p.(Gly694Phefs*23)) detected by high-resolution chromosomal microarray analysis (**d**), and the paternally inherited disease-associated sequence variant c.989_991del (p.(Thr330del)) detected by whole-exome sequencing (**e**)

Applying high-resolution chromosomal copy number profiling and whole-exome sequencing in 63 index cases with EE/DEE and the majority of their healthy parents established a diagnosis in ∼42% of patients. De novo CNVs accounted for ∼10% of the diagnostic yield, which is higher than the 3–5% reported in most of previous studies [4–7], but is in line with a recent report on copy number profiling from exome sequencing data [8] and may be explained by the higher genome-wide resolution for CNV detection of our high-resolution chromosomal microarray analysis in comparison with the lower-resolution microarrays and exome sequencing data previously used. Although CNVs might be identified by read-depth analysis from exome sequencing data with a similar diagnostic yield as by high-resolution microarrays, high-resolution copy-number profiling from exome data requires significantly more confirmatory work-up owing to their lower specificity [31]. Our ∼32% diagnostic yield achieved by whole-exome sequencing is within the range published by previous studies, but due to varying pretesting, patient inclusion criteria and cohort compositions direct comparison is difficult [4, 9–16, 18, 32–34]. Nevertheless, with respect to our ∼16% diagnostic yield from causative de novo sequence variants, which comprise the vast majority in the latter studies, we found the same spectrum of affected genes. As a major difference, however, we additionally found (likely) disease-associated variants in autosomal and X-linked recessive genes in another ∼16% of index patients. This finding is not explained by consanguinity, since only one of ten of these index patients is an offspring of a consanguineous couple. There is also neither a difference in the ratio of EE/DEE diagnoses and age of onset of seizures, nor any other apparent clinical difference between the dominant and recessive disease group. The only previous report of a considerable diagnostic yield of 11% for recessive sequence variants so far was a diagnostic whole-exome sequencing study with limited clinical data [18]. Given the high number of rare inherited variants per patient (Fig. 1b), identification of causative inherited pathogenic variants is much more challenging than the evaluation of de novo variants, and in our study required extensive manual data annotation including interdisciplinary clinical assessment, a comprehensive search for second hits in single-recessive variants as well as functional evidence for pathogenicity of missense variants derived from structural mutation modeling and untargeted metabolome data. Notably, metabolomic analysis increased our diagnostic yield for recessive disease genes by supporting the pathogenicity of missense variants involved in metabolic pathways, as illustrated for Snyder Robinson syndrome and ACO2 deficiency [24, 25]. Moreover, in three cases with biallelic disease-associated variants in known autosomal recessive disease genes, only manual curation unraveled the respective second variant. In these three cases, there was a single-inherited sequence variant in trans with either a de novo sequence or an inherited copy number variant each (Fig. 2). Accordingly, the single-inherited sequence variants were initially filtered out by standard algorithms, since compound heterozygosity is commonly defined as two variants occurring at different genomic positions within the same gene with one variant inherited from each heterozygous parent [35]. As no previous study considered copy-number and sequence variants for compound heterozygous hits, such allele constellations were not called by filtering strategies reported so far. Without these three challenging cases, our diagnostic yield for recessive disease genes would be in the same range as reported by Helbig et al. [18]. These unexpected mechanisms may also explain why we found *SPATA5*, which was rarely reported so far [36–40], as a more common cause of EE/DEE (2/63; 3%). In order to independently prove this high frequency of *SPATA5-*related EE and DEE, we analyzed its prevalence in 39 cases with infantile or childhood epilepsies who received diagnostic exome sequencing and identified one case carrying a previously reported homozygous *SPATA5* disease-associated variant NM_145207.2(SPATA5):c.[251 G > A];[251 G > A] p.[(Arg84Gln)];[(Arg84Gln)]. This diagnostic case with developmental encephalopathy with late-onset epilepsy also had an unusual mutational mechanism with homozygotization of a maternally inherited variant due to maternal isodisomy of chromosome 4. Thus, our overall finding of 3 out of 102 combined research and diagnostic patients carrying causative biallelic variants indicates that *SPATA5* is a frequent cause of developmental epileptic disorders accounting for 3% of cases (Table 4). Notably, in a recently published whole-exome sequencing study of 320 patient–parent trios with EE a single patient with compound heterozygous *SPATA5* pathogenic variants was the only case with autosomal recessive disease cause found [33].

**Table 4:** Clinical characteristics of published and novel *SPATA5* patients

	Patient from our diagnostic cohort	This study ID 73068	This study ID 47651	Tanaka et al.^36^ 14 patients	Kurata et al.^37^ 3 patients	Buchert et al.^38^ 8 patients	Szczauba et al.^39^ 2 patients	Puusepp et al.^40^ 5 patients	Summary of all 35 patients
Variants	HO (UPD)	CH	CH	13 CH, 1 HO	3 CH	1 CH, 7 HO of one family	2 CH	5 CH	26 CH, 9 HO
Age	30 y	5 y 7 m	13 y	2 y – 19 y	1 y – 4 y 7 m	1 y 5 m – 41 y	4 y, 10 m	3 y - 9 y	10 m – 41 y
Sex	Female	Male	Male	8 female, 6 male	1 female, 2 male	3 female, 5 male	2 female	3 female, 2 male	18 female, 17 male
Microcephaly	+	+	-	12/13	+, -, +	7/8	-, -	5/5	28 (80%)
Moderate/severe ID	+	+	+	14/14	+, + , +	8/8	+ , too young^*^	5/5	34 (97%)
Seizures	+	+	+	13/14	+, + , +	1/8	+, -	5/5	26 (74%)
Hearing loss	+	+	-	14/14	+, + , +	4/8	+ , +	5/5	30 (86%)
Hypotonia	+	-	-	13/14	-, +, -	4/8	+, +	2/5	23 (66%)
Spasticity	+	+	+	9/14	+, -, +	-	-, -	5/5	19 (54%)
Visual impairment	+	+	+	13/13	+, NA, NA	3/8	-, -	3/5	23 (66%)
Speech	No word	No word	No word	No word 12/13 one word 1/13	No word (3/3)	Very limited	No word 1/2 too young 1/2	No words	No words or few words
Motor development	Head control, sitting	No head control	No head control	No sitting 11/12 sitting at 3 y 1/12	No head control 3/3	No sitting 1/8 delayed sitting and walking 7/8	Head control 2/2 no sitting 1/2 slightly delayed 1/2	No head control 2/5 no sitting 4/5 walking few steps 1/5	No head control – delayed walking
MRI anomalies	NA	Hypomyelination, progressive atrophy	Cerebral atrophy, hypomyelination, thin corpus callosum	Atrophy 2/12 hypomyelination 3/12 thin corpus callosum 2/12	Cerebral atrophy 2/3 delayed myelination 3/3 thin corpus callosum 3/3	Cerebral atrophy 1/4	-, NA	Atrophy 4/5 delayed myelination 3/5 thin corpus callosum 1/5	Atrophy, delayed myelination, thin corpus callosum
EEG	NA	Abnormal	Abnormal	Abnormal 14/14	Abnormal 3/3	Abnormal 2/2	Abnormal 1/2	Abnormal 5/5	

*Patient too young for proper assessment but shows hypotonia and significant delay of motor development

*CH* compound heterozygous, *EEG* electroencephalogram, *HO* homozygous, *ID* intellectual disability, *m* months, *MRI* magnetic resonance imaging, *NA* not available, *UPD* uniparental disomy, *y* years

Concerning the broad variant filtering applied (minor allele frequency (MAF) < 2%, + /−12 flanking intronic bp, 16% aberrant allele frequency), our findings of three EE-associated variants within three to six flanking intronic basepairs indicate the importance of evaluation of the splicing region beyond the canonical splice site. However, the highest European MAF observed amongst our EE-associated variants was 0.03% (*SPATA5* rs748291365) for recessive disease genes, and one de novo variant (*SCN8A* rs796053229) had a MAF of 0.003% in the Latino population and an overall MAF of 0.0004% in gnomAD. With reference to the aberrant allele frequency, the lowest observed in our cohort was 32% in apparently heterozygous inherited disease-associated variants, but we did not identify a de novo variant below this value indicative of a mosaic status.

It is also noteworthy that our findings further support the disease association of the two recently established disease genes *GABRB2* and *PRUNE1*, as well as the candidacy of *PIK3AP1*, *GTF3C3*, *WRAP53*, and *UFC1* as potential neurodevelopmental disease genes (see Table S5 in the Supplementary Material). To date, only two de novo missense variants in *PIK3AP1* have been reported in two independent patients with infantile spasms [41]. Our patient harbors an unreported de novo missense variant with deleterious predictions in the DBB domain, which is required in other proteins to mediate protein–protein interaction. Further reports and functional studies are needed to evaluate the potential role of this gene in neurodevelopmental disorders.

*GTF3C3* was classified as moderately confident candidate gene in a recently published study on neurodevelopmental disorders in consanguineous families after detection of homozygous missense variants in two affected sisters with a phenotype including mild ID, seizures, and dysmorphism [42]. Another study reported a patient with profound microcephaly, facial dysmorphism, and failure to thrive to harbor a homozygous splice site variant in *GTF3C3* causing skipping of exon 10 and parts of exon 11 (in-frame deletion) [43]. Furthermore, the yeast ortholog of GTF3C3, Tfc4, has been shown to interact with BRF1 to regulate RNA polymerase III-mediated transcription [44, 45]. Biallelic variants in *BRF1* (MIM 604902) cause the cerebellofaciodental syndrome (MIM 616202) with a similar phenotype as our patient (delayed development, intellectual disability, abnormal facial and dental findings, and cerebellar hypoplasia) [46, 47]. Thus, we consider *GTF3C3* as a candidate for neurodevelopmental disorders which may or may not include epilepsy.

Autosomal-recessive variants in *WRAP53* have been reported as disease cause in two unrelated patients with the classical phenotype of Dyskeratosis congenita (DC) (MIM 613988) [48], supported by functional studies of the detected variants [48]. Our patient harbors two variants in compound-heterozygous constellation, NM_018081.2:c.[1034 A > G];[1303 G > A] p.[(Tyr345Cys)];[[(Gly435Arg)] (see Table S5 in the Supplementary Material), of which the first is novel and the latter has been reported and functionally studied by Zhong et al. [48]. As previously described in other DC subtypes [49], our 2 ½ year-old patient shows delayed development, intrauterine growth retardation, cerebellar hypoplasia, microcephaly, sparse fine hair, and bone marrow failure, but does not yet present classical signs of variable age of onset, such as nails dysplasia, abnormal pigmentation, and oral leukoplakia. Additionally, our patient has early onset and therapy resistant absence seizures, which is in line with findings in a DC mouse model [50], but this expansion of the phenotype needs confirmation by further observations.

*UFC1* was first proposed as a candidate gene for recessive ID after the detection of a homozygous missense variant in two siblings with global developmental delay and microcephaly by Anazi et al. [51]. A collaborative effort on this gene revealed in addition to our patient with a homozygous missense variant further five patients from two families harboring a distinct homozygous variant, all sharing a phenotype of early-onset encephalopathy (microcephaly, short stature, global developmental delay, seizures, failure to thrive). This cohort was recently published along with evidence for a hypomorphic effect of both missense variants in functional studies [52]. Of note, variants in *UBA5* and *UFM1* in the same ufmylation pathway have also been described in patients with early-onset encephalopathy [52–56].

With respect to genetic counseling, autosomal or X-linked recessive disorders were identified in 38% of diagnosed cases. Since in two cases with autosomal recessive variants one allele each occurred as de novo alteration, a high-recurrence risk of 25% for further siblings was present in 31% of the diagnosed cases. In contrast to previous assumptions [2], this high-recurrence risk is not restricted to offspring of consanguineous parents, which accounted for a small minority of our cases with recessive inheritance. Thus, the true recurrence risk may have been underestimated due to the low number of pregnancies following such severely affected patients in the hitherto absence of prenatal diagnostic options. Accordingly, as a direct result from the identification of the underlying genetic cause, 19% of all diagnosed families benefited from targeted prenatal testing during this study. Moreover, the genetic diagnosis led to a change in epilepsy treatment in 12% of diagnosed patients. In line with a previous report indicating a higher diagnostic yield of 39% de novo pathogenic variants in very early-onset epilepsy (≤2 months) in comparison with seizure disorders in general (18%) [57], we found a remarkably high-diagnostic yield of 77% de novo and recessive pathogenic variants in patients with seizure onset within the first 2 months in comparison with our whole cohort (∼42%).

We also revealed additional genetic disorders as secondary findings in known disease genes in 10% of patients (Asthenozoospermia, late-onset mild hearing impairment, predisposition to endocrine tumors and adrenal Cushing syndrome, Noonan syndrome 5, left ventricular noncompaction 7, MPO deficiency; Supplementary Table S8). This observation of blended phenotypes is higher than in a broader clinical exome sequencing study [58], but similar to the results of a whole-exome sequencing study in patients with combined phenotypes of metabolic and intellectual developmental disorders [59].

In conclusion, we found evidence of causative copy number and sequence variants in established disease genes explaining together with recurrent candidate genes about 48% of patients with EE/DEE with an unexpected high contribution of inherited recessive disorders, which is neither explained by founder mutations nor by consanguinity, imposing a significant recurrence risk even in outbred populations.

## Electronic supplementary material

Supplemental Material
