# Supplementary material for: The role of recessive inheritance in early-onset epileptic encephalopathies: a combined whole-exome sequencing and copy number study
Source: Eur J Hum Genet. 2018 Dec 14;27(3):408–21. doi: 10.1038/s41431-018-0299-8 (PMC6460568; doi:10.1038/s41431-018-0299-8)
Supplement: Supplementary file 1 — Supplemental Material [file 41431_2018_299_MOESM1_ESM.docx]

## Supplemental Material to

**The role of recessive inheritance in early-onset developmental and epileptic encephalopathies: a combined whole-exome sequencing and copy number study**

This file contains detailed clinical features of the patients; all true positive, rare findings from chromosomal microarray analysis; the summarized (likely) causative findings and their respective ACMG variant classification; recurrent candidate genes; incidental findings; and structural modeling of and functional information on selected variants.

**Table of Content**

Abbreviations 2

**Table S1.** Clinical features 3 - 28

**Table S2.** Rare coding variants detected by chromosomal microarray analysis (CMA) 29 - 32

**Table S3.** Disease-associated sequence and copy number variants in established EE/ID genes or regions 33 - 36

Table S4. ACMG variant classification of disease-associated variants in established genes causing epileptic encephalopathy 37 - 38

Table S5. Details on variants in recurrent candidate genes for neurodevelopmental disorders 39 - 40

**Table S6.** Structural modeling of and functional information on selected variants 41 - 46

**Table S7.** Patients with incidental findings of previously reported or truncating variants in known disease genes 47 - 57

**Table S8.** Summary of relevant findings per patient 58 - 61

**Figure S1.** Interphase FISH with a red STS probe and a green centromeric X probe indicating triplication of the STS gene region in the

female patient 48264. 62

**Web Resources** 63 - 64

**Supplemental References** 65 - 74

**Abbreviations**

**AD** autosomal dominant

**AR** autosomal recessive

**ASD** autism spectrum disorders

**CH** compound heterozygous

**CMA** chromosomal microarray analysis

**CNV** copy number variation

**DN** *de novo*

**EE** epileptic encephalopathy

**EEG** electroencephalography

**DQ** Developmental quotient

**ER** endoplasmic reticulum

**F** female

**fs** frameshift

**HC** head circumference

**HO** homozygous

**ID** intellectual disability

**Kb** kilo basepairs

**L** lenght

**LoF** loss-of-function

**M** male

**m** month

**MAF** minor allele frequency

**Mat** maternal

**Mb** mega basepairs

**na** not available

**NDD** neurodevelopmental disorder

**Pat** paternal

**sib** sibling

**SNP** single nucleotide polymorphism

**VOUS** variant of unknown significance

**w** weeks

**W** weight

**WES** whole exome sequencing

**XL-D** X-linked dominant

**XL-R** X-linked recessive

**y** year

Table S1. Clinical features

| **Patient ID** | **sex** | **Number and sex of affected siblings** | **Number and sex of healthy siblings** | **CMA** | **WES-trio, WES** | **Diagnostic Gene / region (mode of inheritance)** | **Change of drug therapy or diet after diagnosis** | **Prenatal diagnostic provided during course of study** | **Incidental findings** | **Abnormal biochemistry** | **Ethnicity** | **Parental consanguinity** | **Age at last evaluation** | **Initial normal development** | **Indication for initial referral** |
| --- | --- | --- | --- | --- | --- | --- | --- | --- | --- | --- | --- | --- | --- | --- | --- |
| 59248 | f | none | none | yes | WES-trio | **-** | na | na | **DPM1 (carrier)** | no | Caucasian (Swiss) | no | 7y 3m | na | Seizures |
| 66415 | f | none | 1 f | yes | WES-trio | **-** | na | na | **-** | no |  | no | 17y | yes | failure to thrive at 1y 3 mo |
| 73790 | m | 5 m (all siblings speech delay, 1 sib with febrile seizures, 1 sib with epilepsy (CSWS)) | none | yes | WES-trio | **-** | na | na | **-** | no | Caucasian (Swiss) | no | 2y 2m | yes | Febrile seizures, seizures |
| 58962 | m | no | 1 m | yes | WES-trio | **-** | na | na | **-** | no | Caucasian (Swiss) | no | 10y | no | Global developmental delay, dystonic cerebral palsy at 7m |
| 72128 | f | none | none | yes | **-** | **CDKL5 (XL, DN)** | no | na | **-** | no | Caucasian (Swiss) | no | 11m | na | Focal clonic seizures at 6w |
| 73705 | m | none | none | yes | WES-trio | **-** | na | na | **-** | no | Caucasion (Turkish) | first cousins | 4y | no | neonatal |
| 73214 | f | none | none | yes | WES-trio | **KCNQ2 (AD)** | no | na | **-** | mildly elevated pipecolic acid in plasma, elevated pipecolic acid in urine | Caucasian (Italian) | no | 3y4m | no | Seizures (neonatal myoclonic) |
| 71412 | f | none | none | yes | WES-trio | **UBE3A (AD)** | no | **yes** | **OTOF (carrier)** | no | Caucasian (father Swiss, mother Slovene) | no | 2y 5m | no | Absences with febrile infection at age 1y |
| 72984 | m | none | 1 m | yes | WES-trio | **-** | na | na | **-** | no | Caucasian (Swiss) | no | 10y 10m | yes | Infantile spasms at 4m |
| 73237 | f | none | 1 m | yes | WES-trio | **-** | na | na | **-** | no | Caucasian (Swiss) | no | 3y 2m | no | Developmental delay |
| 68942 | m | none | 1 f | yes | WES-trio | **-** | na | na | **-** | no | Caucasian (Swiss) | no | 3y 3m | yes | Focal clonic seizures |
| 72555 | m | 1 m | none | no | WES | **SMS (XL)** | **yes (experimental diet)** | na | **FIG4 (carrier)** | no | Caucasian (South Italian) | no | 2y 11m | no | Global developmental delay |
| 72719 | m | 1 m | none | yes | WES | **SMS (XL)** | **yes (experimental diet)** | na | **FIG4 (carrier)** | no | Caucasian (South Italian) | no | 2y 11m | no | Global developmental delay |
| 71118 | m | none | none | yes | WES-trio | **-** | na | na | **POF1B (carrier)** | no | Caucasian | no | 4y 2m | yes | Seizures |
| 45175 | f | 1 f (learning difficulties and mild overgrowth) | 1 f | yes | WES-trio | **-** | na | na | **-** | no | Caucasian (former Yugoslavian) | no | 14y 11m | yes | Febrile seizures, status epilepticus without fever |
| 72404 | m | none | none | yes | WES | **CDKL5 (XL, DN)** | no | na | **-** | no | Caucasian (Macedonian) | no | 8y 4m | na | Seizures |
| 70757 | f | 1 f (developmental delay, microcephaly, ataxia, febrile seizure) | none | yes | WES-trio | **AP4S1 (AR)** | no | na | **-** | no | Caucasian (Kosovan) | no | 1y 3m | na | Seizures |
| 62075 | f | none | 1 m (half sib) | yes | WES-trio | **-** | na | na | **PRODH (carrier)** | no | Caucasian (father south Italian, mother Swiss/south Italian) | no | 6y 11m | no | Seizures |
| 33386 | f | none | 1 f | yes | WES-trio | **-** | na | na | **-** | no | Caucasian (Swiss) | no | 16y 5m | no | Global developmental delay |
| 73704 | f | none | 2 half sib from mother (1 m (POS), 1 f) side, 3 half sib from father side | yes | WES-trio | **-** | na | na | **-** | no | Caucasian/Asian (mother Swiss, father Tibetan) | no | 10y 8m | no | Global developmental delay |
| 46917 | f | none | 2 half sib from mother (1 m (POS), 1 f) side, 3 half sib from father side | no | WES-trio | **-** | na | na | **-** | no | Caucasian/Asian (mother Swiss, father Tibetan) | no | 10y 8m | no | Global developmental delay |
| 49635 | m | none | 1 m | yes | **-** | **UBE3A (AD)** | no | na | **-** | Decreased neurotransmitters and MTHF in CSF (unclear etiology) | Caucasian (Armenian) | no | 12y 10m | no | Global developmental delay |
| 50126 | m | 1 f (developmental delay), 1 m died at age 24h (premature birth at 28 weeks of gestation) | 1 f | yes | WES-trio | **MBD5 (AD)** | no | na | **-** | no | Caucasian (Kosovan (Roma)) | no | 13y 5m | yes | Seizures |
| 72156 | m | none | 2 f | yes | WES-trio | **-** | na | na | **-** | no | Caucasian (Father Croatian, mother South-Italian) | no | 3y | yes | Seizures |
| 69937 | m | 2 m (same disorder, died at age 4m and 2y 9m) | none | yes | WES-trio | **PRUNE1 (AR)** | no | na | **-** | no | Sri Lankan | 1st degree cousins | 1y 2m | no | Global developmental delay |
| 41637 | m | none | 1 m | yes | WES-trio | **-** | na | na |  | no | Caucasian (Swiss) | no | 18y 5m | yes | Seizures |
| 47970 | m | none | 2 f | yes | WES | **SCN1A (AD)** | **yes** | na | **-** | reduced biopterin and neopterin | Caucasian (Swiss) | no | 17y 6m | na | Seizures |
| 73068 | m | none | 3 half sib (2 m, 1 f) from mother side, 1 half sib (1 m) from father side | yes | WES-trio | **SPATA5 (AR)** | no | na | **-** | no | Caucasian (Swiss) | no | 5y 7m | yes | Seizures with global developmental delay |
| 71592 | m | none | none | yes | WES-trio | **dup 22q11.2 (DN)** | no | na | **RAF1? (AD)** | reduced neopterin in CSF | Caucasian (Swiss) | no | 16y 2m | yes | Seizures |
| 48264 | f | none | 1 f (ADHS) | yes | WES-trio | **-** | na | na | **-** | no | Caucasian (Swiss) | no | 16y 4m | no | Seizures |
| 73175 | m | none | 1 m, 1 f | yes | WES-trio | **-** | na | na | **-** | no | Caucasian (Swiss) | no | 13y 2m | na | Seizures |
| 69733 | m | none | 1 f | yes | WES-trio | **-** | na | na | **-** | no | Caucasian (Kosovan) | no | 3y 2m | no | Seizures |
| 73798 | f | none | 2 m | yes | WES-trio | **-** | na | na | **-** | no | Caucasian (Swiss) | no | 4y 8m | na | Seizures |
| 52236 | f | none | 1 f | yes | WES | **STXBP1(AD)** | no | na | **-** | no | Caucasian (Swiss) | no | 10y 2m | na | Seizures |
| 43990 | m | none | 1 f | yes | WES-trio | **-** | na | na | **-** | mildly decreased HVA/5HIAA ratio, elevated TSH, normal FT4 | Caucasian (Swiss) | no | 12y 9m | no | Global developmental delay |
| 32546 | m | 1 m (same disorder, died at 1y 7m) | none | yes | WES-trio | **-** | na | na | **NPHP1 (carrier)** | no | Caucasian (Swiss) | no | 16y 3m | no | Global developmental delay |
| 72356 | f | none | 1 f | yes | **-** | **1P36DEL (AD)** | no | na | **-** | no | Caucasian (Swiss) | no | 12m | na | Seizures |
| 69986 | f | none | 1 m | yes | WES-trio | **ACO2 (AR)** | no | na | **-** | no | Caucasian (Swiss) | no | 1y 10m | yes | Abnormal visual behavior |
| 70855 | f | none | 1 m | yes | WES-trio | **-** | na | na | **-** | no | Caucasian (mother Bosnian, father Croatian) | no | 3y 3m | no | Developmental regression in the first year |
| 65195 | f | none | none | yes | WES-trio | **-** | na | na | **-** | no | Caucasian (Kosovan) | no | 5y 8m | no | Seizures |
| 73311 | m | 1 m | none | yes | WES-trio | **BRAT1 (AR)** | no | **yes** | **-** | no | Caucasian (Austrian) | no | 6m | na | Seizures |
| 73450 | m | none | none | yes | WES-trio | **-** | no | na | **MIB1, MPO, ACTA1 (carrier)** | macrocytic hyperchromic anemia, thrombocytopenia | Caucasian (mother German, father Swiss/Ghanaian) | no | 2y 6m | yes | Global developmental delay |
| 68047 | m | none | 2 m (1 m died due to astrocytoma at age 5y) | yes | WES-trio | **-** | na | na | **-** | no | Caucasian (Swiss) | no | 4y 1m | no | Global developmental delay |
| 43092 | m | none | none | yes | WES | **SCN8A (AD)** | no | na | **CHST6 (carrier)** | no | Caucasian (mother Russian, father Austrian) | no | 13y 7m | yes | Seizures |
| 34124 | m | none | 3 | yes | WES-trio | **SCN1A (AD)** | no | na | - | no | Caucasian (Swiss) | no | 16y | yes | Seizures |
| 69314 | f | none | none | yes | WES-trio | **-** | no | na | **PDE11A** | elevated pipecolic acid in plasma | Caucasian (Swiss) | no | 3y 7m | no | Gross motor delay and speech development delay |
| 72925 | f | none | 2 f | yes | WES-trio | **-** | no | na | **-** | reduced folate in CSF | Caucasian (Swiss) | no | 21y | yes | Seizures |
| 73134 | m | none | 1 f | yes | WES-trio | **-** | na | na | **-** | no | Caucasian (Swiss) | no | 8y 3m | yes | Seizures |
| 48863 | m | none | 1 m | yes | WES-trio | **-** | na | na | **-** | no | Caucasian (Portuguese) | no | 12y 11m | yes | Seizures |
| 73805 | m | none | none | yes | WES-trio | **STXBP1 (AD)** | no | **yes** | **-** | elevated pipecolic acid in plasma | Caucasian (Italian) | no | 7m | na | Seizures |
| 72892 | m | none | none | yes | WES | **ARX (XL)** | no | **yes** | **-** | no | Afghan | no | 6m | yes | Seizures |
| 56302 | m | 1 m (gait ataxia, developmental delay, behavioral disturbances, celiac disease) | 2 m | yes | WES-trio | **-** | na | na | **GALNTL5** | no | Assyrian | Parents are second degree cousins | 9y 3m | no | Seizures |
| 42680 | m | 1 m (developmental delay, cleft lip and palate) | none | yes | WES-trio | **SCN2A (AD)** | **yes** | na | **-** | no | Caucasian (Swiss) | no | 14y | na | Seizures |
| 73324 | f | none | none | yes | WES-trio | **GABRB2 (AD)** | no | na | **-** | low glycine and elevated neopterin in CSF | Caucasian (Swiss) | no | 6y 6m | yes | Global developmental delay |
| 68944 | m | none | 1 m | yes | WES-trio | **-** | na | na | **GJB2** | no | Caucasian (Swiss) | no | 3y | no | Seizures |
| 71693 | m | 1 f (same disorder, died at age 6y) | 1 m half sib, 1 f half sib | yes | WES | **POLG (AR)** | no | na | **-** | slightly elevated lactate, elevated glycine, elevated TSH | Caucasian (mother Swiss, father Swiss/Austrian) | no | 14y 7m | no | Seizures |
| 69415 | f | none |  | yes | WES-trio | **-** | na | na | **-** | no | Caucasian (Portuguese) | no | 5y 9m | yes, developmental regression along with increase in seizure frequency at age 2y 8m (particularly speech development affected) | Seizures |
| 72440 | m | none | 1 f | yes | WES-trio | **-** | na | na | **ABCC2 (carrier)** | no | Eritrean | no | 7y | na | Seizures |
| 65136 | f | none | 1 f | yes | WES | **-** | na | na | **-** | no | Ghanaian | no | 11y 5m | no | Seizures |
| 75143 | f | none | 4 | yes | WES | **SCN1A (AD)** | no | na | **-** | no | Caucasian (Swiss) | no | 28y | yes | Seizures |
| 75605 | m | none | 1 f (behavioral disturbances, development normal), 1 m | yes | WES-trio | **-** | na | na | **FARS2 (carrier)** | mildly elevated pipecolic acid (urine) | Mother Spanish, father from Bangladesh | no | 8y 11m | yes | Seizures |
| 47651 | m | none | 2 f | yes | WES-trio | **SPATA5 (AR)** | no | na | **-** | no | Caucasian (Swiss) | no | 13y | no | Global developmental delay |
| 72943 | m | none | none | yes | WES-trio | **SZT2 (AR)** | no | **yes** | **-** | no | Caucasian (Swiss) | no | 1y 8m | yes | Global developmental delay |
| 76366 | m | none | none | yes | WES-trio | **-** | na | na | **-** | no | Caucasian (Swiss) | Paternal and maternal grandmothers are second degree cousins | 1y 9m | no | Hyperexcitability, myoclonuses |
| 68681 | f | none | 1 m (25y) | yes | WES-trio | - | na | na | - | no | Caucasian (Italian) | no | 38y | yes | Seizures (infantile spasms) |

Table S1. Clinical features continued

| **Patient ID** | **Age of seizure onset** | **Type of encephalo-pathy** | **Seizure type** | **Developmental achievements** | **Degree of developmental delay or intellectual disability** | **Autistic features** | **MRI findings** | **Neurological anomalies** | **Growth parameters** | **Minor morphological anomalies** | **Additional features** | **Comments** |
| --- | --- | --- | --- | --- | --- | --- | --- | --- | --- | --- | --- | --- |
| 59248 | 3m | EE | Generalized asymmetric tonic-clonic (cluster), tonic | Babbling at 2y, social smile and intermittent smooth eye movements at 5.5y | Severe ID at age 7y 3m (sitting, walking, grasping and speech not achieved) | no | Normal at age 4m, white matter atrophy at age 5y | muscular hypotonia of the trunk (HP:0008936), spastic tetraparesis (HP:0001285), intermittent spontaneous nystagmus(HP:0000639) | HC (<P3) L (P3-10) W (P75-90) | Round face (HP0000311), narrow mouth (HP0000160), sparse eye lashes (HP0000653), epicanthus (HP0000286), short nose (HP0003196), anteverted nares (HP0000463), low-set frontal hairline (HP0000294), wide intermammillary distance (HP0006610) | Hypothyreosis, bruxism |  |
| 66415 | 1y 11m | DEE | Generalized tonic-clonic, focal aware, focal impaired awareness, absences, | Sitting at 7 mo walking at 2y 4mo 100 words at 5 y | severe ID  DQ 45 at 3y9m 100 words, rarely 2-word sentences | at 5y | Normal at age 2y and age 14y | Spastic tetraplegia (HP:0002510), broad-based gait (HP0002136) | HC (<P25-50) L (P50-75) W (P10-25) | full cheeks (HP0000293), short ears (HP04000005), small phalanges of the hand (HP0009803), bil. Fifth digit clinodactyly (HP0004209) | Severe myopia |  |
| 73790 | 14m | EE | Tonic, clonic, focal aware, focal impaired awareness (absence-like) | Social smile at 3m, rolling over at 6m, walking at 12, 2 words at 2y 1m | Mild speech and cognitive delay | no | Unspecific white matter changes in the right-sided frontal region at age 1y 3m | Mild facial hypotonia (HP:0000297), excessive salivation (HP:0003781) | HC (P10-25) L ? W (P10) | High, narrow palate (HP0002705), low hanging columella (HP0009765), short philtrum (HP0000322), everted upper lip vermilion (HP0010803), small, wide-spaced teeth (HP0000691), abnormality of canine (HP0011078), wide nasal bridge (HP0000431), low-set ears (HP0000369), dry hair (HP0011359), frontal upsweep of hair (HP0002236), displacement of 3rd toe under 2nd toe left II (HP0010338), displacement 3rd toe under 4th toe right (HP0010338), short distal phalanx of the thumb (HP0009650) |  |  |
| 58962 | 7m | DEE | Epileptic spasms, focal impaired awareness | Absent | Severe ID at 9y 8m (sitting, walking, speech and visual fixation not achieved) | na | White matter atrophy, delayed myelination and decreased NAA peak and mildly reduced choline peak in the white matter at age 7m and age 3y 6m | Sensorineural deafness (HP0000407), nystagmus (HP0000639), absent visual fixation points and smooth eye movements, muscular hypotonia (HP0001252), spastic-dystonic tetraparesis, marked upper limb dystonia (HP0002451) | HC (<P3) L (<P3) W (<P3) | absent | central disturbance of vision, episodic tachypnea (HP0002789) |  |
| 72128 | 6w | EE | Epileptic spasms, tonic, clonic, eye lid myoclonia | Social smiling at 6w, rolling to the side at 8m | Moderate ID at age 11m (motor skills 3m, cognitive skills 4m, speech skills 3m) | na | Normal at age 2m | Axial hypotonia (HP0001252), partial visual fixation and abnormality of ocular smooth pursuit (HP:000061)7, intermittent convergent squint (HP0000486) | HC (<P3) L (P50-75) W (P25-50) | full cheeks (HP0000293), brachycephaly (HP0000248), anterior plagiocephaly (HP0011326) |  |  |
| 73705 | abnormal EEG at 3-4 mo, seizures at 2y | DEE | Epileptic spasms, generalized tonic-clonic | no speech, no walking | severe (sitting, walking and speech not achieved) | na | Cerebral microangiopathy with periventricular calcifications | Severe axial hypotonia (HP0001252), spastic tetraparesis (HP:0001285), progressive macrocephaly | HC (P>97) L (P10-25) W (P50-75) | none | gastrostomy | Clinical suspicion of Aicardi-Goutier syndrome, but pleiocytosis was excluded in cerebral fluid and interferon alpha was normal |
| 73214 | neonatal | EE | Tonic, myoclonic | None | Severe ID at age 5y (sitting, walking, head control and speech not achieved) | na | Normal at age 1y 1m and 2y 4m | Muscular hypotonia (HP:0001252), convergent squint | HC (<P3) L (P10-25) W (P75-90) | mild hypertrichosis, synophrys, convergent strabism, short neck, low set ears, upslanting palpebral fissures, asymmetric face, tapering fingers |  |  |
| 71412 | 1y | DEE | Absences | Sitting at 1y 6m, crawling at 1y 7m, standing at 2y 5m, babbling at 2y 5m | Severe ID at 2y 5m (walking and speech not achieved) | na | na | Axial (HP0001252) and facial (HP0000297) hypotonia, truncal ataxia (HP0002078), standing and gait ataxia (HP0002066), divergent right-sided squint (HP0000486) | HC (<P3) L (P>97) W (P90-97) | brachycephaly (HP0000248), low-set ears (HP0000369), downslanted palpebral fissures (HP0000494) | Friendly and cheerful character |  |
| 72984 | 4m | EE | Epileptic spasms, generalized tonic -clonic, tonic, myoclonic, focal impaired awareness (absence-like) | Rolling over at 12m, sitting at 1y 3m, standing at 4y 4m | Severe ID at 10y 10m (free walking and speech not achieved) | no | Normal at age 7m (slight difference in myelination between right and left hemisphere) and 2y 1m | Axial hypotonia (HP0001252), spastic paraparesis (HP0002313) | HC (<P3) L (P25-50) W (P25-50) | Pectus carinatum (HP0000768), wide mouth (HP0000154), macrodontia of permanent maxillary central incisors (HP0000675) | Partial heterochromia iridis right eye (HP0001100) |  |
| 73237 | 1y 6m | DEE | Clonic, focal impaired awareness, (partially fever- and infection-triggered) | Sitting at 1y 2m, walking at 2y, babbling at 2y 3m | Severe ID at 3y 2m (walking and speech not achieved) | no | Normal at age 11m. Small frontal lobes (microcephaly) and mesial temporal sclerosis on the right side at age 3y 2m | facial hypotonia (HP0000297), excessive salivation (HP0003781), Axial hypotonia (HP0001252), limb ataxia (HP0002070), gait ataxia (HP0002066), intermittent head titubation (HP0002599) | HC (<P3) L (P10-25) W (P10-25) | High, narrow palate (HP0002705), tented-upper lipvermilion (HP0010804), full cheeks (HP0000293), downslanted palpebral fissures (HP0000494), broad eyebrows (HP0011229),narrow forehead (HP0000341), macrotia (HP0000400), bilat. short distal phalanx of the 5th finger (HP0004227) , dorsal hand edema, bilat. 2-3 toe syndactyly (HP0005709) |  |  |
| 68942 | 3m 3w | EE | Tonic, clonic, focal impaired awareness | Sitting at 2y 4m, aided walking at 3y 3m, first words at 3y 3m | Moderate ID at 3y 3m (free walking not achieved, 2-3 words) | no | punctate periventricular T2 hyperintense foci at age 3m, periventricular calcifications at age 3m (CT), white matter atrophy (HP0002500) and corpus callosum hypoplasia at age 2y 3m | Axial hypotonia (HP0001252), spastic left-sided hemiparesis (HP0011099) | HC (<P3) L (P<3) W (P10-25) | Absent |  |  |
| 72555 | 12m | DEE | Epileptic spasms, tonic, atonic, atypical absences | Social smile at 5m, rolling to the side at 5m, babbling at 5m | Severe ID at 2y 11m (motor skills 5-6m, fine motor, speech, visual and cognitive skills 3-4m; sitting, walking and grasping not achieved) | no | Hypoplasia of corpus callosum (HP0002079) and slight cerebral cortical atrophy(HP0002120) at 13m | Axial hypotonia (HP0001252), lower limb hypertonia (HP0002509), choreoathetosis (HP0001266) at age 29m | HC (<P3) L (P50) W (P75) | Thin upper lip vermilion (HP0000219), high, narrow palate (HP0002705), smooth philtrum (HP000319), epicanthus(HP0000286), downslanted palpebral fissures (HP0000494), ptosis (HP0000508), dry hair (HP0011359), fingernail dysplasia (HP0100798) |  |  |
| 72719 | 12m | DEE | Tonic, atonic myoclonic, atypical absences | Babbling at 5m, sitting, walking and grasping not achieved | Severe ID at 2y 11m (motor skills 5-6m, fine motor, speech, visual and cognitive skills 3-4m) | no | Isolated bilateral pallidal hyperintensity at age 14m | Axial hypotonia (HP0001252), lower limb hypertonia (HP0002509), | HC (<P3) L (P50) W (P75) | Thin upper lip vermilion (HP0000219), high, narrow palate (HP0002705), smooth philtrum (HP000319), epicanthus (HP0000286), downslanted palpebral fissures (HP0000494), dry hair (HP0011359), fingernail dysplasia (HP0100798) |  |  |
| 71118 | 1y 9m | EE | Epileptic spasms, tonic, atonic, myoclonic | Sitting at 6.5m, walking at 13m, first words at 9m (lost again) | Severe ID at 4y 2m (speech skills 15m) | no | Normal at age 17m. Left-sided hippocampal sclerosis at age 2y 11m | Axial hypotonia (HP0001252), standing ataxia | HC (P50-75) L (P25-50) W (P75-90) | Absent |  |  |
| 45175 | 1y 9m | EE | Generalized tonic-clonic, clonic (partially fever-associated) | Reported normal until 21m, walking at 13m, developmental regression (speech) at 4 years | Severe ID, DQ 2y at age 3y 10m, rarely 2-word sentences at 14y 11m | no | Normal at age 1y 9m. Cerebral atrophy at age 4y 10m | Gait ataxia (HP:0002066) | HC (P>97) L (P50-75) W (P50-75) | large ears (HP0000400), long fingers (HP0100807) and long toes (HP0010511) | Scoliosis |  |
| 72404 | 1m | EE | Generalized tonic-clonic, tonic | Absent | Severe ID at 8y 4m (sitting, walking, speech and visual fixation not achieved) | no | na (reported normal) | Nystagmus (0000639), absent fixation (Poor eye contact HP0000817), facial and axial hypotonia (HP0000297, HP0008936), right-sided divergent squint(HP:0000486), spastic tetraparesis (HP:0001285), Excessive salivation (HP:0003781), dysphagia (HP:0002015) | HC (P25-50) L (P50-75) W (P75-90) | High, narrow palate (HP0002705) | Scoliosis, recurrent pulmonary infection due to dysphagia |  |
| 70757 | neonatal | EE | Tonic, focal aware, focal impaired awareness | Grasping at 8m, rolling over at 11m, sitting and babbling (syllables) at 16m | Moderate ID at 1y 3m | no | Aqueductal stenosis with consecutive hydrocephalus internus at age 1m | Axial hypotonia (HP0001252), truncal ataxia (HP:0002078), spastic paraparesis (HP:0002313), alternating esotropia (HP:0000565) | HC (P90-97) L (P75-90) W (P75-90) | narrow mouth (HP:0000160) | Joint hyperlaxity |  |
| 62075 | 10m | EE | Tonic, myoclonic, hyperkinetic (pedaling), atypical absences, non-convulsive status epilepticus | Social smile at 6w, rolling over at 5m | Severe ID at 6y 11m (sitting, walking, speech, visual fixation and smooth pursuit eye movements not achieved) | no | Initial evaluation reported normal. Reevaluation reported dysmorphic hemispheres, frontal lobe hypoplasia with simplified gyration at age 3y 4m | Axial hypotonia (HP0001252), spastic paraparesis (HP:0002313), alternating divergent strabism (HP:0000486) | HC (P<3) L (P90-97) W (P25-50) | Short philtrum (HP:0000322), Microretrognathia (HP:0000308), high, narrow palate (HP0002705), Microdontia (HP:0000691),Sparse eyebrow (HP0000535), Abnormality of the hairline (HP0009553) ,Tapered finger (HP0001182), displacement 2nd toe over 3rd toe bilat. (HP:0010326) | Bilateral myopia, sleep disorder, slow hair growth |  |
| 33386 | 4y 3m | DEE | Generalized tonic-clonic, myoclonic (reflex seizures) | Babbling and laughing, partial visual fixation at 16y 4m | Severe ID at 6y 11m (sitting, walking and speech not achieved) | no | Corpus callosum hypoplasia at 8m. Severe cerebral and marked cerebellar atrophy with simplified gyral pattern at age 15y 11m | Axial hypotonia (HP:0001252), spastic dyskinetic tetraparesis HP:0001285, abnormal conjugate eye movement(HP:0000549) and Impaired smooth pursuit (HP:0007772) | HC (P<3) L (P10-25) W (P<3) | high, narrow palate (HP:0002705), Abnormality of the hairline (HP:0009553), widow` peak (HP:0000349), broad eyebrow (HP:0011229), Macrodontia of permanent maxillary central incisor (HP:0000675), diastema (HP:0000699), short distal phalanx of finger (HP:0009882), overlapping toe (HP:0001845), short foot bilat. (HP:0001773) | Scoliosis, choreoathetotic movement disorder at age 2m to 6m |  |
| 73704 | 5y 5m (suspected earlier) | DEE | Generalized tonic-clonic, tonic, myoclonic, myoclonic-atonic | Sitting at 12m, standing and aided walking at 3y, absent speech development | Severe ID ad 10y 8m | no | Unspecific T2-hyperintense lesion in the medial frontal gyrus (gliosis) otherwise normal at age 5y | Spastic tetraparesis (HP:0001285), esotropia (HP:0000565) | HC (P3-10) L (P<3) W (P<3) | high, narrow palate (HP0002705), narrow face (HP:0000275) | Scoliosis, marked increase in seizure frequency at age 6y with loss of developmental achievements, feeding difficulties |  |
| 46917 | 5y 5m (suspected earlier) | DEE | Generalized tonic-clonic, tonic, myoclonic, myoclonic-atonic | Sitting at 12m, standing and aided walking at 3y, absent speech development | Severe ID at 10y 8m (sitting, walking, speech and visual fixation not achieved) | no | Unspecific T2-hyperintense lesions in the capsula interna and putamen on postnatal images. Unspecific white matter signal alteration and atrophy of the cerebellar vermis at age 6y 8m | Spastic tetraparesis (HP:0001285), right-sided divergent squint (HP:0000486), dysphagia (HP:0002015) | HC (P3-10) L (P10-25) W (P25-50) | High, narrow palate (HP:0002705), Hypopigmentation of the skin along Blaschko lines on the right flank (HP:0001010), narrow face (HP:0000275) | Marked increase in seizure frequency at age 7y with loss of developmental achievements |  |
| 49635 | febrile seizures at 3y 10m, seizures at 6y 4m | DEE | Febrile seizures, generalized tonic-clonic, focal impaired awareness | Sitting at 2y, walking at 3y | Severe ID at 12y 10m (speech not achieved) | no | Normal at age 3y 10m | Gait ataxia (HP:0002066), left-sided esotropia (HP:0000565) | HC (P10-25) L (P90-97) W (P25-50) | prominent nose (HP0000448) | Intermittent aggressive behavior, cheerful character |  |
| 50126 | 1y 7m | EE | Febrile seizure (first seizures), generalized tonic-clonic, tonic | Walking at 1y 2m, 3 words at 3y | Severe ID at 13y 5m (absent speech, deterioration of walking ) | no | Normal at age 2y 10m. Bilateral thalamic T2-hyperintensity with central hypointensity, T2 hyperintensities in the right hippocampus, in the dorsal left hippocampus, in the periaequaductal gray region, in the subthalamic nucleus, in the dorsal lateral pons and multiple cortical T2 hyperintensities at age 2y 11m. Parenchymal lesions in the same structures as described above at age 9y 8m | Gait ataxia (HP:0002066), spastic paraparesis(HP:0002313), excessive salivation (HP:0003781) | HC (P<3) L (P<3) W (P25-50) | Thick eyebrows (HP:0000574), Deeply set eye (HP:0000490) | Hyperactive behavior, scoliosis, acute necrotizing encephalopathy after infection at age 2y 11m, generalized tremor and right-sided hemiparesis with infection at age 3y 2m |  |
| 72156 | 9m | EE | Focal impaired awareness | Rolling over at 4m, sitting at 5-6m, babbling at 6m, walking at 11m | Severe ID at 3y, DQ 41, several words, no sentences, motor development age-appropriate | yes | Normal at age 1y. Highly suspicious of focal cortical dysplasia in the anterior temporal lobe and hippocampal dysplasia at age 2y 11m | Impaired social interactions (HP:0000735) | HC (P90-97) L (P>97) W (P>97) | Wide nasal bridge (HP:0000431), hypertelorism (HP:0000316), epicanthus (HP:0000286) | Aggressive behavior, autoaggressive behavior (headbanging, biting) at age 12m, loves music, hand stereotypies. Sleep disturbances at age 2y 9m |  |
| 69937 | 6m | DEE | Epileptic spasms, focal aware, tonic, myoclonic | Absent | Severe ID at 1y 2m | no | Immature cortex differentiation (35 GW), punctate cerebellar hemorrhages, discrete signs of hypoxia, increased lactate peak in the white matter at age 35 4/7 weeks of gestation | Muscular hypotonia (HP:0001252), spastic tetraparesis (HP:0001285) | HC (P<3) L (P3-10) W (P3-10) | Retrognathia (HP:0000278), sacral mongolian blue spot (HP:0011369), Deviation of the 3rd toe over 4th toe bilat. (HP:0010332) | Premature birth at 35 4/7 weeks of gestation, recurrent apneas |  |
| 41637 | 12m | EE | Epileptic spasms, atonic, tonic absences, focal impaired awareness (BNS-like) | Responsive smile at 2m, sitting 8m, walking 11m, thereafter developmental stagnation | Severe ID at 18y 5m (absent speech) | yes | Mild cerebellar atrophy, suspicion of beginning mesial temporal sclerosis at 4y 6m | Muscular hypotonia of the trunk (HP:0008936), gait disorder (external rotation of feet) (HP:0003783) | HC (P90-97) L (P75-90) W (P90-97) | Abnormality of the hairline (HP:0009553),Pes planus (HP:0001763), deeply set eye (HP:0000490), kyphosis(HP:0002808) | Arm stereotypies, emotional behavioral disorder, intermittent autoaggressive behavior |  |
| 47970 | 2m | EE | Focal, generalized tonic-clonic, status epilepticus (infection-triggered), absences | Sitting 6m, crawling 12, standing 15m | Severe ID at 18y 6m (absent speech, aided walking) | no | Normal at 4m | Spastic tetraplegia (HP:0002510), excessive salivation (HP:0003781) | HC (P3-10) L (P<3) W (P<3) | High, narrow palate (HP:0002705), wide nasal bridge (HP:0000431), abnormality of canine (HP:0011078) and 12th tooth |  |  |
| 73068 | 5m | EE | Epileptic spasms, generalized tonic-clonic, tonic, focal aware | Babbling at 6m (lost again), visual fixation and smooth pursuit eye movement at 1y | Severe ID at 5y 7m (sitting, walking, grasping and speech not achieved) | no | Normal at age 6m. Supratentorial white matter atrophy and delayed myelination at age 13m. Supra- and infratentorial white matter atrophy at age 2y 9m. | sensorineural hearing impairment (HP:0000407), spastic tetraparesis (HP:0001285), pendular nystagmus (HP:0012043), Esotropia (HP:0000565), dysphagia (HP:0002015) | HC (P<3) L (P50-75) W (P25-50) | Long (HP:0000276), narrow (HP:0000275) face, epicanthus (HP:0000286), wide nasal bridge (HP:0000431), high, narrow palate (HP:0002705), edema of the dorsum of feet (HP:0012098), Thoracic hypoplasia (HP:0005257), hyperpigmentation of the skin on thigh (HP:0000953) | Poor pigmented retina, hyperopia, secundum-type atrial septal defect with left to right shunting, diastolic dysfunction |  |
| 71592 | febrile seizures at 2y, seizures at 3y 3m | EE | Febrile seizures, epileptic spasms, myoclonic-atonic, atonic, clonic | Walking at 13m, several words at 2y | Severe ID at 16y 2m | no | Normal at age 3y 6m | muscular hypotonia of the trunk (HP:0008936), gait ataxia (HP:0002066), crouch gait, myoclonus (HP:0001336) | HC (P<3) L (P<3) W (P<3) | kyphosis (HP:0002808) | Diagnosis of colitis ulcerosa at age 14 y 8m, |  |
| 48264 | febrile seizures at 1y 10m, seizures at 2y | DEE | Febrile seizures, myoclonic, atonic, absences, (ESES-like EEG at 11y) | Visual fixation at 9m, sitting at 12m, crawling at 13m, walking at 1y 8m, 2-word sentences at 4y, loss of speech at 8y | Severe ID at 16y 4m, DQ 55-60 at 3y | no | Mild cerebral atrophy and mildly elevated lactate peak in the basal ganglia and in the parietal white matter, thrombosis of sigmoid sinus and right-sided sinus rectus at age 10y 11m | muscular hypotonia of the trunk (HP:0008936), excessive salivation (HP:0003781) | HC (P50-75) L (P<3) W (P25-50) | Dysostosis multiplex (HP:0000943), coarse facial features (HP:0000280), full cheeks (HP:0000293), narrow nasal tip (HP:0011832), long fingers (HP:0100807) | Joint hyperlaxity of upper distal extremities, behavioral and sleep disorder at 8y (together with loss of speech and ESES-like EEG) |  |
| 73175 | neonatal | EE | Epileptic spasms, generalized tonic-clonic, tonic, myoclonic | Partial visual fixation at 7m, rolling to the side and babbling at 9m | Severe ID at 13y 2m (sitting, walking and speech not achieved) | no | Postnatal MRI with hippocampal malrotation. Diffuse white matter atrophy with frontal preponderance, delayed myelination at age 11m | Muscular hypotonia of the trunk (HP:0008936), spastic tetraplegia (HP:0002510), nystagmus (HP:0000639) | HC (P3-10) L (P<3) W (P<3) | widely spaced teeth (HP:0000687), extra concha fold (HP:0400002), mild hypoplasia of midface (HP:0011800), left-sided perioral and chin freckling (HP:0001480), mild pectus excavatum (HP:0000767), long (HP:0100807) and tapered finger (HP:0001182) |  |  |
| 69733 | 11m | EE | Generalized tonic-clonic, febrile seizures | Crawling at 9m, sitting at 10m, walking at 1y 7m, first words ad 13m, 2 words at 3y | Severe ID at 3y 2m, DQ 36 at 3y | no | Normal at age 12m | mild muscular hypotonia HP:0001252), mild ataxia (HP:0001251), mild dysmetria (HP:0001310) | HC (P25-50) L (P25-50) W (P75-90) | upslanted palpebral fissure (HP:0000582), large fleshy ears (HP:0002265) |  |  |
| 73798 | neonatal | EE | Apneas, febrile seizures, generalized tonic-clonic, tonic (cluster), myoclonic, status epilepticus | Rolling over at 9m, sitting at 12m, standing at 4y 4m, babbling at 2y 6m | Severe ID at 4y 8m (walking and speech not achieved) | no | Normal at age 2y 1m | muscular hypotonia HP:0001252), pes planus valgus (HP:0001763) | HC (P50-75) L (P<3) W (P25-50) | wide nasal bridge (HP:0000431), thin anteverted nares (HP:0004495), upslanted palpebral fissure (HP:0000582), epicanthus (HP:0000286), short ear (HP:0400005), full upper lip vermilion (HP:0000215), smooth philtrum (HP:0000319), small hands (HP:0200055), dry skin (HP:0000958), asymmetry of the thorax (HP:0001555) | Intermittent autoaggressive behavior (headbanging, biting), hyperactive behavior, stereotype head shaking, sleep disorder from age 2y to 4y, eating disorder reduced pain sensation |  |
| 52236 | 1m | EE | Focal impaired awareness, eyelid myoclonia | Sitting at 2y 6m, crawling at 2y 9m, walking at 3y 4m, babbling and syllables at 2y 9m, first words at 4y 10m | Severe ID at 10y 2m, 10 words at 10y 2m | yes | Normal at age 1m | muscular hypotonia of the trunk (HP:0008936), gait ataxia (HP:0002066), mild action tremor (HP:0002345) | HC (P25-50) L (P10-25) W (P75-90) | Hypertelorism (HP:0000316), short chin (HP:0000331), macrodontia of permanent maxillary central incisor (HP:0000675) with diastema (HP:0000699), protruding ear (HP:0000411), kyphosis (HP:0002808), truncal obesity (HP:0001956), pes planus valgus (HP:0001763), sandal's gap (HP:0001852) | Mild myopia, hyperactive behavior, marked weight gain at age 6m, eating disorder (absent feeling of satiety), sleep disorder at age 7y |  |
| 43990 | 7m | DEE | Epileptic spams, generalized tonic-clonic, hemitonic, clonic (eyelid myoclonia and hyperkinetic aspects) | Visual fixation at 3w, aided standing at 6y 9m | Severe ID at 12y 9m (sitting, walking and speech not achieved) | no | Mild cerebral atrophy at age 6m. Microcephaly with progressive loss of associative and commissural myelination at age 3y 11m. | muscular hypotonia of the trunk (HP:0008936), spastic tetraparesis (HP:0001285), partial visual fixation and abnormality of ocular smooth pursuit (HP:000061), persisting oral exploration | HC (P<3) L (P3-10) W (P3-10) | wide nasal bridge (HP:0000431), hypotelorism (HP:0000316), epicanthus (HP:0000286), hyperpigmentation of the skin left lower leg (HP:0000953), generalized joint laxity (HP:0002761) | Small for gestational age (placental insufficiency), nephrocalcinosis of unclear etiology, liver failure of unclear etiology at age 3y (regressive after discontinuation of ketogenic diet), pubarche at age 4y, periodic respiration and central apneas at age 7m, central disturbance of vision |  |
| 32546 | febrile seizures at 7m, seizures at 1y 2m | DEE | Febrile seizures, generalized tonic-clonic, hemiclonic, tonic, fever-and non-fever associated status epilepticus | Rolling over at 10m, sitting at 1y 2m, babbling (syllables) at 1y 5m, first words at 2y, several words at 5y | Severe ID at 16y 3m, DQ 50 at 12m, DQ 44 at 3y (absent speech) | no | Mild frontal cerebral atrophy and delayed myelination at 1y 1m. Progressive frontal and temporal cerebral atrophy, delayed myelination at 1y 8m. | muscular hypotonia of the trunk (HP:0008936,spastic tetraparesis (HP:0001285), Gait ataxia (HP:0002066) | HC (P25-50) L (P50-75) W (P25-50) | wide nasal bridge (HP:0000431), short (HP:0003196),hypoplastic nasal tip (HP:0005278),highly arched eyebrow (HP:0002553), synophris (HP:0000664), high, narrow palate (HP:0002705), Increased laxity of fingers (HP:0006149), pes planus valgus (HP:0001763), decreased palmar crease (HP:0006184) | Premature birth at 36 GW, hand stereotypies, hyperactivity, mild scoliosis, structural vertebral anomalies (coronal clefting of the lumbar fourth vertebra with narrow disc interspace at T12 and L1 with possible coronal clefting at the T8 and T4) |  |
| 72356 | neonatal | EE | Apneas, focal myoclonic (periorbital), myoclonic, tonic, clonic | Babbling at 6m, grasping at 11m | Moderate to severe ID at 12m (sitting and walking not achieved) | na | Normal at age 1m | Left-sided sensorineural deafness (HP:0000407), muscular hypotonia (HP:0001252), down-beat nystagmus (HP:0010545), alternating divergent strabism (HP:0000486), partial visual fixation and abnormality of ocular smooth pursuit (HP:0000617) | HC (P<3) L (P25-50) W (P10-25) | narrow mouth (HP:0000160), high, narrow palate (HP:0002705), thin vermilion border (HP:0000233), hypoplasia of midface (HP:0011800), hypoplastic nasal bridge (HP:0005281), synophris (HP:0000664), deeply set eyes (HP:0000490), pes planus valgus (HP:0001763), single transverse palmar crease (HP:0000954), nevus flammeus nuchae (HP:0007616), deviation of the 2nd toe over 3rd (HP:0010326) | Postnatal sucking weakness, central disturbance of vision, ostium secundum atrial septal defect |  |
| 69986 | 7m | EE | Generalized myoclonic, tonic, focal impaired awareness, status epilepticus | Social smile and babbling at 2.5m, intermittent active grasping at 14m, response to sounds and touch | Severe ID at 1y 10m (sitting, walking and speech not achieved) | no | Pallidal signal hyperintensity signal hyperintensity on diffusion weighted images and borderline lactate peak within the basal ganglia and periventricular white matter at age 8m. Marked cerebral and cerebellar atrophy and diffuse leukencephalopathy at age 2y 5m. | muscular hypotonia of the trunk (HP:0008936), spastic paraparesis (HP:0002313), horizontal pendular nystagmus (HP:0007811), esotropia (HP:0000565), absent visual fixation and abnormality of ocular smooth pursuit (HP:0000617) | HC (P<3) L (P10-25) W (P25-50) | no | Progressive feeding difficulties and secondary microcephaly at age 8m. Progressive optic atrophy and retinopathy. Disease progression and neurological impairment with viral infections. Recurrent nightly hypoventilation episodes of unknown etiology without cyanosis from the age 2y 8m on. |  |
| 70855 | 3y 2m | DEE | Generalized tonic-clonic, myoclonic | Social smile at 2m, rolling over 5m, babbling (syllables) at 6m, sitting at 7m | Severe ID at 3y 2m , DQ 23 at 1y 11m (walking and speech not achieved, loss of purposeful hand function) | no | T2-hyperintensities on the level of the cella media at age 1y 7m. Mild paratrigonal T2-hyperintensitiesl at age 2y 7m. | muscular hypotonia of the trunk (HP:0008936), standing and gait ataxia (HP:0002066), intermittent hyperventilation (HP:0002883), partial visual fixation | HC (P3-10) L (P90-97) W (P75-90) | no | Hand stereotypies, developmental regression with loss of speech and purposeful hand function at the end of the first year of life, crying episodes |  |
| 65195 | 2.5m | EE | Epileptic spasms, myoclonic, focal impaired awareness, fever- and infection associated recurrent status epilepticus | Walking at 2y, first word at 4y, 2-word sentences at 5y | Moderate to severe ID at 5y 8m | no | Normal at age 4m (mild enlargement of the outer CSF space) | muscular hypotonia of the trunk (HP:0008936), mild gait ataxia (HP:0002066) | HC (P50-75) L (P25-50) W (P>97) | wide nasal bridge (HP:0000431), inverted nipples (HP:0003186), short fifth finger bilat. (HP:0009237), pes planus valgus (HP:0001763), truncal obesity (HP:0001956), flat occiput (HP:0005469) |  |  |
| 73311 | neonatal | EE | Myoclonic | absent | Severe ID | no | na | spastic tetraparesis | HC (P25) L (P25-50) W (P25-50) | na |  |  |
| 73450 | 2y | DEE | Absences | crawling at 9m, sitting at 2y, walking at 2y 2m, 40 words at 2y 6m | Moderate ID at 2y 6m, DQ 75 at 12m | no | Cerebellar hypoplasia and cerebellar-medullary arachnoid cyst at age 2y 2m | muscular hypotonia of the trunk (HP:0008936), gait ataxia (HP:0002066) | HC (P<3) L (P<3) W (P<3) | prominent forehead (HP:0011220), thin and sparse hair (HP:0008070), generalized joint laxity (HP:0002761), pes planus valgus (HP:0001763) | Intrauterine growth retardation, premature birth at 32 GW (placental insufficiency), atopic dermatitis, failure to thrive, thrombocytopenia, sleep disorder |  |
| 68047 | febrile seizures at 11m, status epilepticus without fever at 1y 6m | DEE | Generalized tonic-clonic, atonic, absences, focal impaired awareness, fever- and infection associated recurrent seizures/status epilepticus | sitting at 6m, rolling over at 7m, standing at 1y 4m, walking at 2y 2m, 3 words at 4y 1m | Severe ID at 3y 1m | no | Periventricular white matter atrophy (residual periventricular leukomalacia), retrocerebellar arachnoid cyst, megacisterna magna and incomplete left-sided hippocampal rotation at age 1y 4m and age 2y 4m | Muscular hypotonia (HP:0001252), gait ataxia (HP:0002066), right-sided spastic hemiparesis (HP:0011099), excessive salivation (HP:0003781) | HC (P25-50) L (P10-25) W (P10-25) | high, narrow palate (HP:0002705), bifid tongue (HP:0010297), pseudo strabism, wide nasal bridge (HP:0000431), short nose (HP:0003196), long (HP:0100807) and tapered fingers (HP:0001182), abnormality of the hairline (HP:0009553), increased laxity of fingers (HP:0006149), short palpebral fissures (HP:0012745), broad eyebrow (HP:0011229), hyperpigmentation of the skin on the left knee (HP:0000953), frontal upsweep of hair (HP:0002236) | Intermittent autoaggressive behavior in emotional situations |  |
| 43092 | 3m | EE | Generalized tonic-clonic, tonic, focal secondarily generalized seizures with apneas and cyanosis | Social smile at 1m, babbling (syllables) at 6m, rolling over 7m, sitting 9m, walking at 9y 3m | Severe ID at 13y 7m, DQ 11 at 4y 3m (absent speech) | no | Normal at age 4m. Cerebral atrophy at age 9m. | Muscular hypotonia (HP:0001252), gait ataxia (HP:0002066), | HC (P50-75) L (P50-75) W (P10-25) | Long fingers (HP:0100807), decreased palmar crease (HP:0006184), pectus excavatum (HP:0000767) | Recurrent fractures in the context of seizures at age 1y, generalized osteopenia, failure to thrive in the second year of life, hand stereotypies (washing movements), scoliosis, bruxism |  |
| 34124 | 4.5m | EE | Focal clonic (secondarily generalized), generalized tonic-clonic with cyanosis, myoclonic, absences, recurrent status epilepticus, infection-triggered seizures | Sitting 7m, walking 3y 6m | Severe ID at 16y, DQ of 50 at 1y 8m (absent speech) | yes | Normal at age 4m. Cerebral atrophy, enlargement of Virchow Robin spaces in the parieto-occipital lobes and the anterior commissure at age 1y 7m and 13y 11m. | Muscular hypotonia (HP:0001252), mild gait ataxia (HP:0002066) | HC (P25-50) L (P50-75) W (P10-25) | Absent | Aggressive, destructive behavior and crying episodes from the age of 4y 6m on, sleep disorder |  |
| 69314 | 1y 11m | DEE | Generalized tonic-clonic, myoclonic-atonic | Sitting at 11m, walking at 2y 9m, 5 words at 2y 1m, 10 words and 2-word sentences at 3y 10m | Moderate ID at age 3y 7m, DQ of 63 at age 2y 9m | no | Normal at age 2y | Muscular hypotonia (HP:0001252), mild gait ataxia (HP:0002066) | HC (P75-90) L (P3-10) W (P50-75) | Frontal bossing (HP:0002007), short ears (HP:0400005), depressed nasal ridge (HP:0000457) |  |  |
| 72925 | 2.5m | EE | Epileptic spasms, generalized tonic-clonic, tonic, atonic myoclonic, absences | Social smile at 7w, rolling to the side at 5y 10y | Severe ID at age 21y (sitting, walking and speech not achieved) | no | Normal at age 3m and 5m. Cortical, marked cerebellar atrophy, right-sided reduced hippocampal volume at age 7y 5m | Muscular hypotonia of the trunk (HP:0008936), spastic tetraparesis (HP:0002273), partial visual fixation, abnormality of ocular smooth pursuit (HP:0000617), dysphagia (HP:0002015) | HC (P<3) L (P3-10) W (P<3) | high, narrow palate (HP:0002705), thoracic hypoplasia (HP:0005257), long fingers(HP:0100807) overlapping finger 2nd-3rd, hort foot bilat. (HP:0001773), hypertrichosis of forearms (HP:0000998) | Scoliosis (spondylodesis) |  |
| 73134 | 3y 4m | EE | Generalized tonic-clonic, tonic, atonic, myoclonic, status epilepticus | Sitting 7m, walking 12m, first words 13m, 2-word sentences 15m, developmental regression and behavioral disturbances at age 5y with increase in seizure frequency | Moderate ID at 8y 3m (neuropsychological evaluation with verbal IQ 93, performance IQ 85 and working speed IQ 45 at 6y, neuropsychological evaluation with global developmental delay at 7y 10m) | no | Normal at age 4y 2m and 7y. | facial hypotonia (HP:0000297), excessive salivation (HP:0003781), mild right-sided spastic hemiparesis (HP:0011099), gait ataxia (HP:0002066) | HC (P50-75) L (P25-50) W (P50-75) | wide nasal bridge (HP:0000431), macrodontia of permanent maxillary central incisor (HP:0000675), increased laxity of fingers (HP:0006149), camptodactyly (HP:0012385) 5th bilat. |  |  |
| 48863 | 6m | EE | Generalized tonic-clonic, clonic, focal impaired awareness | Sitting at 14m, walking at 2y, first words at 2y | Severe ID at age 12y 11m, DQ 30 at age 3y | no | Slightly reduced choline peak in the right-sided hippocampal region, otherwise normal at age 3y 8m | Fine motor difficulties | HC (P>97) L (P25-50) W (P90-97) | Truncal obesity (HP:0001956), left inverted nipple (HP:0003186), macrotia (HP:0000400), thick lower lip vermilion (HP:0000179), long fingers (HP:0100807), finger syndactyly (HP:0006101), Valgus foot deformity (HP:0008081) | Hyperactive behavior |  |
| 73805 | neonatal | EE | Tonic, clonic myoclonic, epileptic spasms, fever- and infection triggered seizures | Social smile at 2m, rolling to the side at 4m, grasping at 7m | Moderate ID at age 7m | na | Bilateral hyperintense ischemic lesion in the posterior limb of the capsula interna, reduced NAA peak and elevated lactate peak in the left-sided basal ganglia at age 1d | muscular hypotonia of the trunk (HP:0008936), spastic paraparesis (HP:0002313), inspiratory stridor (HP:0005348), choreatiform and atactic movement disorder of upper extremities (HP:0007028, HP:0007337, HP:0006811, HP:0002469) | HC (P50-75) L (P90-97) W (P50-75) | Sloping forehead (HP:0000340), high, narrow palate (HP:0002705), thoracic hypoplasia (HP:0005257), pectus excavatum (HP:0000767), prominent fingertip pads (HP:0001212) |  |  |
| 72892 | 8w | EE | Epileptic spasms, clonic | Social smile at 2m, visual fixation at 1m, loss of achievements after onset of seizures | Severe ID at age 6m | na | Normal at age 2m | Muscular hypotonia of the trunk (HP:0008936), spastic tetraparesis (HP:0002273), left-sided divergent strabism (HP:0000486), partial visual fixation and abnormality of ocular smooth pursuit (HP:0000617) | HC (P25-50) L (P10-25) W (P50-75) | Tented upper lip vermilion (HP:0010804) |  |  |
| 56302 | 4y 3m | DEE | Tonic, clonic, eyelid and mouth myoclonia, atypical absences | Crawling at 24m, rolling over at 36m, aided walking at 3y 7m (lost again after orthopedic operation), grasping at 5y | Severe ID at age 9y 3m (sitting and speech not achieved) | na | Supra- and infrantentorial cerebral atrophy, mild thalamic atrophy and atrophy of caudate nucleus at age 1y 11m. Supra- and infratentorial cerebral atrophy at age 3y 7m | Muscular hypotonia of the trunk (HP:0008936), spastic tetraparesis (HP:0002273), excessive salivation (HP:0003781) | HC (P<3) L (P3-10) W (P3-10) | Narrow face (HP:0000275), prominent nose (HP:0000448) and convex nasal ridge (HP:0000444), long eyelashes (HP:0000527), high, narrow palate (HP:0002705), thick lower lip vermilion (HP:0000179), exaggerated cupid's bow (HP:0002263), abnormality of the hairline (HP:0009553), tapered fingers (HP:0001182), hypertrichosis of arms, legs and back (HP:0000998), brittle scalp hair (HP:0004779) | Sleep disorder, failure to thrive, frequent infections of the upper respiratory tract, bilateral cataract |  |
| 42680 | neonatal | EE | Tonic (serial), myoclonic, apneas | Social smile at 1y, occasional rolling to the side in the second half of the first year | Severe ID at age 14y (sitting, walking and speech not achieved) | no | Normal at age 4d, generalized supra- and infratentorial atrophy, bilateral hippocampal atrophy, left-sided hippocampal sclerosis and atrophy of corpus callosum at age 14y | Axial hypotonia, spastic tetraparesis, horizontal nystagmus, partial visual fixation, absent smooth ocular eye pursuit, hypersalivation | HC (P10-25) L (P<3) W (P<3) | high, narrow palate (HP:0002705) | Sleep disorder, intermittent autoaggressive behavior at age 11y, central disturbance of vision, feeding difficulties |  |
| 73324 | 2y 4m | DEE | Generalized tonic-clonic, tonic, myoclonic, atypical absences, subclinical status epilepticus | Rolling to the side at 9m, sitting 1y 2m, crawling and standing at 4y 4m, aided walking at 5y, babbling (syllables) at 2y 11m | Severe ID at age 6y 6m (speech not achieved) | no | Normal at age 2y 1m, delayed myelination at age 3y 7m, normal at age 5y 9m | muscular hypotonia of the trunk (HP:0008936), facial myoclonuses, multifocal myoclonuses, standing and gait ataxia, convergent squint | HC (P<3) L (P<3) W (P75-90) | Deeply set eye (HP:0000490), straight eyebrows (HP:0011228), pes planus valgus (HP:0001763), hypopigmented nevus left thigh)(HP:0001053, Dig. I and III cross Dig. II right foot, Dig. II. and Dig. III cross Dig I left foot | Intermittent (often infection- or fever-triggered) neurological deterioration (crisis) with frequent myoclonic seizures, encephalopathy and loss of vision (first time at 2y, 1-3x/year), hand stereotypies |  |
| 68944 | 2m | EE | Generalized tonic-clonic, tonic, clonic, eyelid myoclonia | Social smile at 7w, babbling at 2m, rolling over at 15m, aided sitting at 2y | Severe ID at age 3y (walking and speech not achieved) | no | Bilateral white matter signal hyperintensity in the parietal lobes, elevated lactate peak in the parietal left-sided region at age 3m. Bilateral periventricular and frontal white matter signal hyperintensity at age 1y 11m | Marked muscular hypotonia of the trunk (HP:0008936), horizontal nystagmus (HP:0000666), partial visual fixation and abnormality of ocular smooth pursuit (HP:0000617) , dysmetria (HP:0001310), intermittent myoclonuses | HC (P3-10) L (P75-90) W (P10-25) | pectus excavatum (HP:0000767), widely spaced teeth (HP:0000687) | Floppy infant at 1m |  |
| 71693 | 1y 6m | DEE | Atonic, eyelid myoclonia, focal clonic (Epilepsia partialis continua) | Walking at 21m, first words at 24m, 2-word sentences at 4y | Moderate ID at age 14y 7m | no | Normal at age 3y 2m. Low-grade glioma in the left thalamus, diffusion restriction in the left occipital region at age 10y 4m | muscular hypotonia of the trunk (HP:0008936), truncal and gait ataxia (HP:0002078), (HP:0002066), areflexia of lower limbs (HP:0002522), discrete pes cavus (HP:0001761) | HC (P<3) L (P<3) W (P<3) | wide mouth (HP:0000154), highly arched eyebrow (HP:0004533) | Failure to thrive, short stature, friendly character, electrophysiology suspicious of sensory axonal neuropathy (reduced amplitudes) |  |
| 69415 | 1y 4m | EE | Epileptic spasms, tonic, atonic, myoclonic-atonic, atypical absences | Walking at 18m, babbling (syllables) at 6m, first words at 13m, 2-word sentences at 2y 6m | Moderate ID at age 5y 9m | yes (at age 3y) | Normal at 1y 8m and 3y | mild muscular hypotonia of the trunk (HP:0008936) | HC (P10-25) L (P50-75) W (P50-75) | Broad forehead (HP:0000337), straight eyebrows (HP:0011228), long eyelashes (HP:0000527), narrow nasal tip (HP:0011832), smooth philtrum (HP:0000319), widely spaced teeth (HP:0000687), retrognathia (HP:0000278) | Sleep disorder, hyperactive behavior and crying episodes from age 2y 8m on |  |
| 72440 | 4y 3m | EE | Generalized tonic-clonic (clusters), tonic, status epilepticus | Walking at 13m, babbling (syllables) at 6m, first words at 18m, 2-word sentences at 24m | Moderate ID at age 7y | no | Bilateral hippocampal hyperintensity (reported as not pathological due to the MRI-sequence) at age 6y 10m | no | HC (P25-50) L (P25-50) W (P90-97) | no | Impulsive and hyperactive behavior, postictal aggression |  |
| 65136 | 6m | EE | Epileptic spasms, generalized tonic-clonic, tonic | Sitting at 2y 11m, crawling at 5y 2m, aided walking at 11y, babbling at age 5y 2m | Severe ID at age 11y 5m, DQ 10 at age 5y 2m (walking and speech not achieved) | no | Normal at age 5m and 1y 6m | Alternating divergent strabism (HP:0000486), facial hypotonia (HP:0000297), lower limb hypertonia (HP:0006895) | HC (P<3) L (P10-25) W (P10-25) | Brachycephaly (HP:0000248), wide nasal bridge (HP:0000431), broad forehead((HP:0000337), widow's-peak (HP:0000349), telecanthus (HP:0000506), hypoplasia of midface (HP:0011800), short philtrum (HP:0000322), everted lower lip vermilion (HP:0000232), dental crowding (HP:0000678), diastema (HP:0000699), bilateral camptodactyly 5th (HP:0100490), brachydactyly (HP:0001156), 3-4 toe syndactyly (HP:0009779), pes planus valgus (HP:0001763) | Optic atrophy at age 2y 2m, failure to thrive at age 7y (due to impaired swallowing), premature adrenarche of unknown etiology at 4y 3m, hand and truncal stereotypies, recurrent pneumonias, atypical intrathoracic mycobacterial infection |  |
| 75143 | 3m | EE | Generalized tonic-clonic, tonic, focal impaired awareness, reflex seizures (photosensitivity) | Standing at 9m, first words at 10m, 10 words at 18m | Severe ID at age 28y (walking lost at age 10y, max. 40 words, lost in the first 10y) | no | Cerebellar atrophy at age 15y | Spastic tetraparesis | HC ? L (P<3) W (P3) |  | Scoliosis |  |
| 75605 | 5m | EE | Epileptic spasms, atonic | Walking at 14m, first words at 12m | Severe ID at age 8y 11m (30 words) | no | Left-sided hippocampal atrophy and right-sided temporo-polar arachnoid cyst at age 7y 4m | muscular hypotonia of the trunk (HP:0008936), gait disorder (forefoot internal rotation and frequent staggering), intermittent tongue fasciculations (HP:0001308) | HC (P25-50) L (P10-25) W (P>97) | high, narrow palate (HP:0002705), Incisor macrodontia (HP:0011081), diastema (HP:0000699), large earlobe (HP:0009748), mild ptosis (HP:0000508), abnormality of muscle of facial expression (HP:0430019), inverted nipples (HP:0003186), hyperlordosis (HP:0003307), talipes calcaneovarus (HP:0008124 | Sleep disturbance (HP:0002360), sleep apnea syndrome (HP:0002870), polyphagia (HP:0002591) |  |
| 47651 | 8m | DEE | Epileptic spasms, tonic, clonic, atonic, absences, gelastic seizures | Grasping at age 4m (lost in the further course) | Severe ID at age 13y (sitting, walking, grasping and speech not achieved) | no | Normal at age 4m, delayed myelination and reduced NAA peak at age 8m, cortical atrophy at age 16m, progressive but reduced myelination, white matter atrophy, frontal bilateral supratentorial atrophy, thin corpus callosum, reduced NAA peak, elevated lactate and myoinositol peak (MRS) at age 12y | Horizontal nystagmus (HP:0000666), dystonia of upper extremities (HP:0002451), spastic tetraparesis (HP:0001285) | HC (P3-10) L (P<3) W (P3-10) | none | Sleep disturbance |  |
| 72943 | 6m | EE | Epileptic spasms, generalized tonic-clonic, focal impaired awareness | Grasping at 4,5m, rolling to the side at 9m, rolling over at 12m, babbling at 12m, crawling at 14m | Severe ID at age 1y 8m (sitting and grasping not achieved) | na | Dysmorphic corpus callosum, septations in the frontal ventricles at age 7m, polymicrogyria in the frontal lobes, in the right-sided parietal lobe | muscular hypotonia (HP:0001252) | HC (P>97) L (P>97) W (P50-75) | Broad forehead (HP:0000337), low-set short ears (HP:0400005), retrognathia (HP:0000278), short chin (HP:0000331), high, narrow palate (HP:0002705), lumbar ribs (HP:0000772), pectus carinatum (HP:0000768), coronal hypospadias (HP:0008743) | generalized joint laxity (HP:0002761), nuchal lipoma (HP:000103), reduced pain sensitivity |  |
| 76366 | 3m | EE | Epileptic spasms, clonic, tonic, myoclonic | Babbling at 16m | Severe ID at age 1y 9m (grasping, sitting, walking and speech not achieved) | na | Delayed myelination at age 4m | Generalized dystonia (HP:0007325), spastic tetraparesis (HP:0001285), inspiratory stridor (HP:0005348), absent fixation | HC (P<3) L (P<3) W (P>97) | High, narrow palate (HP:0002705), tented upper lip vermilion (HP:0010804), full cheeks (HP:0000293), hypoplasia of midface (HP:0011800), large earlobes (HP:0009748), short fingers (HP:0009803) | Hyperexcitability and myoclonuses in the first months, secondary failure to thrive, sleep disorder, recurrent vomiting |  |
| 68681 | 4m | EE | Epileptic spasms, generalized tonic-clonic (weather sensitive (snowing)), tonic, atonic, myoclonic, atypical absences | walking age 2y, first words at 3y, loss of speech, but some comprehension at age 38y | loss of skills, moderate-severe ID (walking, no speech) | yes | na | muscular hypotonia (HP:0001252) | 1.62 cm, 57 kg, HC 54.2 cm | broad forehead (HP:0000337), broad and straight eyebrows (HP:0011228) with mild synophrys (HP:0000664), abnormal teeth positon (HP:0000678), prominent chin (HP:0000303), large ears (HP:0000400), short nails (HP:0001799) | thoracic kyphosis, scoliosis; Menarche with 15 years |  |

Table S2. Rare coding variants detected by chromosomal microarray analysis (CMA) (technical details on variant calling described elsewhere[^1^](#_ENREF_1))

| **Patient ID**  **Decipher ID** | **Sex** | **Genome location (hg19)** | **Copy number** | **Size (kb)** | **Inheritance** | **Gene content** | **Classification concerning ID/EE** | **Comments**  **(copied from OMIM, NCBI Gene database, expression from The Human Protein Atlas, or taken from cited references)** |
| --- | --- | --- | --- | --- | --- | --- | --- | --- |
| 59248  Decipher 286320 | F | chr1:171867341-172239637 | 1 | 372 | **DN** | DNM3 | likely benign | Few coding deletions in DECIPHER population copy-number variants and DGV; Ashraf et al, 2015[^2^](#_ENREF_2) described a partial deletion of DNM3 and of 2 microRNAs (harbored within intron 14 of this gene: miR199 and miR214) in a patient with normal intelligence and skeletal abnormalities and conclude that haploinsufficiency for DNM3 is unlikely to be the sole cause for cognitive impairment in 1q24q25 deletion syndrome and proposed miR199 and miR214 as likely candidates for the striking skeletal phenotype;  Sanger sequencing of the DNM3 gene revealed no variant; the deletion of this patient was already reported in Asadollahi et al., 2014[^1^](#_ENREF_1) |
| 66415  Decipher 370004 | F | chr1:60004763-60046291 | 1 | 42 | Mat | FGGY | likely benign | Frequent coding deletions in DGV and DECIPHER population copy-number variants; highest expression in liver and muscle, FGGY carbohydrate kinase domain containing no second variant in WES, gene fully covered |
| 66415  Decipher 370004 | F | chr10:35080426-35166805 | 3 | 86 | Mat | PARD3 | benign | Partial duplication of PARD3, inherited from the healthy mother, reported in DGV |
| 72128  Decipher 370072 | F | chrX:18592741-18617503 | 1 (mosaic) | 25 | **DN** | CDKL5 | **pathogenic** | **Variant Rett syndrome**; confirmation by MLPA |
| 72128  Decipher 370072 | F | chr4:176203171-176719979 | 3 | 517 | **DN** | GPM6A | VOUS | Partial duplication of GPM6A reaching far outside the 3’UTR; few coding deletions in DGV; only one entire duplication of this gene reported in a girl with learning disability and behavioral problems[^3^](#_ENREF_3) ; this patient has also a *de novo* CDKL5 deletion fully explaining the phenotype |
| 73705  Decipher 370085 | M | chrX:140641814-140740125 | 2 | 98 | Mat | SPANXA2-OT1 | VOUS | Non-coding RNA; duplications in this region have already been observed in the female control population; also observed in DGV, but gender unknown |
| 73705  Decipher 370085 | M | chr10:49387490-49422916 | 1 | 35 | Pat | FRMPD2 | VOUS | Reported in few DECIPHER population copy-number variants, E-cadherin dependent recruitment of FRMPD2 to cell-cell junctions, knockdown in Caco-2 cells is associated with impairment of tight junction formation[^4^](#_ENREF_4); highest expression in brain and testis no second hit in this gene in WES, but last exons very GC-rich not covered; parents are first cousins |
| 73214  Decipher 370067 | F | chr2:63926544-64280712 | 1 | 354 | Mat | UGP2, VPS54 | VOUS | Few coding deletions in DGV in VPS54 and few small deletions in coding region of UGP2; bigger deletions in DECIPHER; vacuolar protein sorting-associated protein 54 expressed in brain; mouse model: Loss of Vps54 function leads to the phenotype of amyotrophic lateral sclerosis (motor neuron degeneration with retrograde vesicle traffic)[^5^](#_ENREF_5); UPD-glucose pyrophosphorylase 2 expressed in brain, highest in muscle and liver WES revealed a causative *de novo* variant in KCNQ2 |
| 71412  Decipher 370084 | F | chr15:23620191-28545355 | 1 | 4900 | **DN** | 105 genes, including UBE3A | **pathogenic** | **Angelman syndrome** confirmation of deletion of the maternal allele by methylation sensitive MLPA |
| 68942  Decipher 286324 | M | chrX:122803456-122986898 | 3 | 83 | Mat-grandfather | THOC2 | benign | Variants in THOC2 are causing X-linked mental retardation 12 (MIM #300957) The same duplication was found in the healthy maternal grandfather |
| 73704  Decipher 370069 | F | chr3:37783051-37950009 | 3 | 167 | Pat | ITGA9, CTDSPL | benign | Three similar duplications in DGV |
| 49635  Decipher 370081 | M | chr15:25583408-25717757 | 1 | 134 | **DN** | UBE3A maternal copy | **pathogenic** | **Angelman syndrome** SNP analysis showed deletion of maternal allele |
| 50126  Decipher 370070 | M | chr2:148762374-148959158 | 1 | 197 | **DN** | ORC4, MBD5 | **pathogenic** | **2q23.1 deletion syndrome** |
| 50126  Decipher 370070 | M | chr11:117956262-118021740 | 3 | 65 | Pat | LOC100526771, TMPRSS4, SCN4B | likely benign | Partially affecting the longest isoforms of TMPRSS4 and SCN4B genes; 2 duplications reported in DGV; SCN4B reported in Atrial fibrillation, familial, 17 (AD) or Long QT syndrome-10 (AD); 2 duplications in DGV |
| 50126  Decipher 370070 | M | chr1:54435448-54482725 | 3 | 47 | Pat | LDLRAD1 | benign | Partially affecting LDLRAD1; two duplications reported in DGV; DECIPHER only larger duplications entirely covering this gene and others without details on inheritance and functional effects |
| 69937  Decipher 370089 | M | chr20:60288507-60321403 | 1 | 33 | Pat, Mat | CDH4 | likely benign | Not segregating with the phenotype; intragenic deletion which according to the raw data may not even affect an exon; homozygous deletion is present in the deceased brother with same phenotype; smaller losses in this region not affecting the exon deleted in our patient arereported in DGV, one big loss in DGV, 3 small deletions in this gene in DECIPHER: 288495, 273606, 268127 not clearly disease-associated. CDH4 is expressed in testis, brain, prostate, and fetal brain. Based on studies in chicken and mouse, this cadherin is thought to play an important role during brain segmentation and neuronal outgrowth. In addition, a role in kidney and muscle development is indicated (NCBI GeneID 1002) |
| 69937  Decipher 370089 | M | chr15:64479739-64661380 | 1 | 182 | Pat, Mat | CSNK1G1, KIAA0101 | likely benign | Not segregating with the phenotype; the same deletion but homozygous is present in the likewise affected brother also; no reports in DGV; a deletion bigger than ours 1.79 Mb reported in DECIPHER (249717) in a patient with ID, microcephaly, seizures, and other phenotypical features; in a child from Bangladesh with early infantile epileptic encephalopathy and microcephaly, Martin et al., 2014 identified a *de novo* heterozygous c.688C-T transition in the CSNK1G1 gene, resulting in an Arg230Trp substitution at a highly conserved residue in the catalytic domain. The variant was found by whole genome sequencing and confirmed by Sanger sequencing; functional studies of the variant were not performed[^6^](#_ENREF_6) |
| 73068  Decipher 370090 | M | chr14:41825601-42169236 | 1 | 344 | Mat | LRFN5 | VOUS | Deletion of LRFN5 reported in autism (DECIPHER ID 284540, this patient has another 2 small deletions); in mice, Lrfn5 expression was reported to be limited to mature nervous tissue[^7^](#_ENREF_7); no CNVs affecting this region in DGV |
| 71592  Decipher 370076 | M | chr22:22953514-25026857 | 3 | 2073 | **DN** | 45 genes | **pathogenic for milder phenotype** | **Recurrent 22q11.22 q11.23 duplication.** OMIM-genes: BCR, IGLL1, CHCHD10, SMARCB1, MIF, SPECC1L, UPB1 |
| 48264  Decipher 370094 | F | chrX:6449752-8135644 | 4 | 1686 | 1 copy DN, 2 copies Pat | VCX3A, HDHD1, MIR4767, STS, VCX, PNPLA4, MIR651 | VOUS | Healthy father and healthy sister have duplication (2 and 3 copies, respectively); duplication not significantly more common in patients than in controls, insufficient data for triplication; mildly skewed X-inactivation (72:28) in patient; Interphase FISH results: one X with one STS copy and one X with three STS copies (Figure S2) |
| 48264  Decipher 370094 | F | chr17:4042587-4129786 | 3 | 87 | Pat | ZZEF1, CYB5D2, ANKFY1 | likely benign | ZZEF1 and ANKFY1 are partially affected; only one large duplication and other partial duplications of this region reported in DGV. DECIPHER: 277486 - likely benign duplication, 271494 - duplication inherited from a healthy parent; ANKFY1 synaptic gene |
| 73175  Decipher 370079 | M | chr3:11727760-11797881 | 3 | 70 | Pat | VGLL4 | benign | del/dup reported in DGV |
| 73175  Decipher 370079 | M | chrY:6172738-9164654 | 0 | 2992 | Pat | AMELY, TBL1Y, PRKY | benign | DECIPHER: many deletions reported as benign or unknown disease-association |
| 73175  Decipher 370079 | M | chrY:9542706-9699626 | 0 | 157 | Pat | 0 | benign |  |
| 52236  Decipher 370078 | F | chr4:72842899-73222937 | 3 | 380 | Mat | NPFFR2, ADAMTS3 | likely benign | ADAMTS3 is partially duplicated; no DGV reports; variants in ADAMTS3 reported once in schizophrenia[^8^](#_ENREF_8) and in NPFFR2 reported once in autism[^9^](#_ENREF_9)  causative STXBP1 variant in WES |
| 52236  Decipher 370078 | F | chr6:168173794-168223121 | 3 | 49 | Mat | 0 | benign | Reported in DGV causative STXBP1 variant in WES |
| 52236  Decipher 370078 | F | chr1:217951508-217972125 | 1 | 21 | Pat | SPATA17, SPATA17-AS1 | likely benign | One big deletion reported in DGV; DECIPHER: 256543 - the smallest deletion including this gene and 8 others is 3.53Mb (unknown disease-association) reported *de novo* in a patient with ataxia, ptosis, muscular hypotonia, Talipes equinovarus  causative STXBP1 variant in WES; in SPATA17 no second variant affecting function, well covered in WES |
| 32546  Decipher 370080 | M | chr2:110504318-111365996 | 1 | 862 | NA | **NPHP1** | **recessive allele** | Deletions in this region have already been observed in the control population (DGV: one report with 5 deletions in 873 samples[^10^](#_ENREF_10)). Recessive allele for nephronophthisis 1 and Joubert syndrome 4 (MIM *607100) In WES NPHP1 gene is completely covered - no second variant affecting function detected |
| 72356  Decipher 370071 | F | chr1:0-7666975 | 1 | 7700 | **DN** | 118 | **pathogenic** | **1p36 deletion syndrome** |
| 65195  Decipher 370092 | F | chr6:41250398-41257744 | 1 | 7 | **DN** | TREM1 | likely benign | No DGV reports, only large deletions reported in DECIPHER, the smallest deletion (277771) includes at least 30 other genes and the clinical details are not available; expected LoF 7.8 and observed 6 in ExAC. This gene encodes a receptor belonging to the Ig superfamily that is expressed on myeloid cells. This protein amplifies neutrophil and monocyte-mediated inflammatory responses triggered by bacterial and fungal infections (NCBI GeneID 54210), highly expressed in blood, not in brain. Confirmed by WES-CNV analysis, no second variant affecting function in WES |
| 73450  Decipher 370095 | M | chr1:229545536-229577325 | 1 | 32 | Pat | **ACTA1**, NUP133 | **recessive allele** | ACTA1 causes AR Nemaline-Myopathy 3 (OMIM 161800);  no second variant affecting function in WES |
| 43092  Decipher 370088 | M | chr2:106719520-107272634 | 3 | 553 | NA | UXS1, RGPD3 | likely benign | Partial duplication of UXS1; 2 large deletions including PLGLA, RGPD3 reported in DGV, no small duplications in DECIPHER, only one of 5 MB (264805) inherited from a parent with similar phenotype (no clinical details); UXS1 - expressed in brain, RGPD3 - high expression in testis |
| 7395  Decipher 370087 | F | chr15:101955447-102041488 | 3 | 86 | NA | PCSK6 | likely benign | Partial duplication of PCSK6. Duplication in this region have already been observed in the DGV database. The encoded protease is constitutively secreted into the extracellular matrix and expressed in many tissues, including neuroendocrine, liver, gut, and brain. This gene is thought to play a role in tumor progression and left-right patterning  WES revealed causative SCN8A variant |
| 73134  Decipher 370091 | M | chr11:62838126-62861409 | 1 | 23 | Pat | SLC22A24 | likely benign | Deletions in this region have already been observed in the DGV database. High expression of rat Slc22a24 in liver and skeletal muscle and in coronal sections taken from the olfactory bulb and forebrain. Expression was weaker in other cortical sections, and none was detected in other specific brain regions or in peripheral tissues[^11^](#_ENREF_11). Human: expressed in liver and kidney  WES revealed VOUS in SCN1A, no second variant affecting function in SLC22A24 in WES and gene well covered |
| 73805  Decipher 370086 | M | chr10:5225299-5429298 | 1 | 204 | Mat | AKR1CL1, AKR1C4, UCN3 | likely benign | One big deletion reported in DGV, small deletion not covering the UNC3 also reported in DGV. DECIPHER: only one small deletion (VOUS) covering AKR1CL1, AKR1C4 (288284) maternally inherited in a patient with tall stature, abnormality of skin pigmentation, bell-shaped thorax, micrognathia and abnormality of toe; one small deletion (288783) of AKR1C4 gene reported as likely benign (unknown inheritance) in a patient with specific learning disability; AKR1C4 - expressed in liver, highest expression in pituitary; variants in AKR1C4 are reported as modifier of 46XY sex reversal 8 (MIM *600451); WES revealed a causative variant in STXBP1 gene; no second variants affecting function, genes well covered in WES |
| 73805  Decipher 370086 | M | chr11:108669930-108691491 | 1 | 22 | Mat | DDX10 | benign | Intragenic deletion, not affecting the coding region of DDX10 gene. One loss in DGV. Only large deletions affecting numerous genes reported in DECIPHER  WES revealed a causative variant in STXBP1 gene; no second variants affecting function, genes well covered in WES |
| 56302  Decipher 370075 | M | chrX:6446579-8135644 | 2 | 1689 | Mat | VCX3A, HDHD1, MIR4767, STS, VCX, PNPLA4, MIR651 | VOUS | The mother and 3 brothers are also carriers of the duplication; the mother has a random X-Inactivation of 49:51, 2 of the brothers are healthy, one has mild developmental problems including ataxic gait, visuomotor impairment, abnormal behavior and short attention span |
| 71693  Decipher 370093 | M | chr15:84587641- 85075449 | 3 | 488 | Mat | ZSCAN2, WDR73, NMB, SEC11A, ZNF592, ALPK3, SLC28A1, PDE8A | likely benign | No DGV reports, no duplications reported in DECIPHER, but 4 deletions: 248182 - deletion of 774 kb in this region in a patient with tall stature and ulnar deviation of finger, another 3 deletions partially covering this region: 300045 (delayed speech and language development, esophageal atresia - maternally inherited), 290103 (autism, ID - unknown inheritance), 308155 (without clinical details); WDR73 gene variants in Galloway-Mowat syndrome (AR, neurodegenerative disorder characterized by infantile onset of microcephaly and central nervous system abnormalities resulting in severely delayed psychomotor development. Brain imaging shows cerebellar atrophy and sometimes cerebral atrophy. More variable features include optic atrophy, movement disorders, seizures, and nephrotic syndrome (MIM #251300))  WES revealed causative CH variants in POLG and a VOUS heterozygous variant in KCNQ2 (not present in the mother, father not available testing) |
| 71693  Decipher 370093 | M | chr22:42815447-42955616 | 3 | 140 | Mat | ARFGAP3, PACSIN2 | likely benign | Partial duplication of the involved genes; no DGV reports, no duplications reported in DECIPHER, only large deletions reported in NDD |
| 72440  Decipher 370073 | M | chr1:110178180-110264600 | 1 | 86 | Pat | GSTM4, GSTM2, GSTM1, GSTM5 | likely benign | Partial deletions and duplications reported in DGV, no small deletions reported in DECIPHER. Large deletions including more than 10 genes reported in patients with ID. GSTM1 encodes an enzyme expressed in liver and peripheral blood, while the product of GSTM2 is expressed in muscle (MIM *138380) GSTM4, GSTM2, GSTM5 well covered and no second variant affecting function in WES; GSTM1 not covered |
| 65136  Decipher 370066 | F | chrX:7039463-8135644 | 3 | 1683 | not Mat | VCX3A, HDHD1, MIR4767, STS, VCX, PNPLA4, MIR651 | VOUS | Frequency in in-house database of controls 0.3-0.4%, more common in females than in males, several studies discussing it as risk factor for developmental disorders and seizures[^12^](#_ENREF_12)^,^[^13^](#_ENREF_13) father not available for testing |
| 65136  Decipher 370066 | F | chr9:33140975-33261725 | 3 | 121 | not Mat | B4GALT1, SPRINK4, BAG1 | likely benign | Not in mother, father not available for testing; partially affecting B4GALT1 and BAG1 genes; duplication in this region observed in DGV |
| 75143  Decipher 370068 | F | chr18:718854-852388 | 1 | 134 | Mat | YES1 | VOUS | Deletions reported in DGV, only large deletions reported in DECIPHER; YES proto-oncogene 1, Src family tyrosine kinase; tyrosine kinase activity and belongs to the src family of proteins (NCBI GeneID 7525). Non-receptor protein tyrosine kinase, involved in the regulation of cell growth and survival, apoptosis, cell-cell adhesion, cytoskeleton remodeling, and differentiation. Plays a role in cell cycle progression (UniProtKB P07947) WES revealed a DN variant in SCN1A, no second variant affecting function in WES, good coverage |
| 75143  Decipher 370068 | F | chr1:247893618-247941192 | 1 | 48 | Mat | OR1C1 | benign | Large deletions reported in DGV, Olfactory Receptor Family 1 Subfamily C Member 1 WES revealed a DN variant in SCN1A, no second variant affecting function in WES, good coverage |
| 75605  Decipher 370077 | M | chr6:5172637-5454482 | 1 | 282 | Mat | LYRM4, **FARS2** | **recessive allele** | Variants in FARS2 reported in AR Combined oxidative phosphorylation deficiency 14 (MIM #614946) CNV analysis of WES data confirms deletion in patient and his mother and does not show further exon deletions/duplications |
| 47651  Decipher 370082 | M | chr2:203705909-203782735 | 1 | 77 | Pat | ICA1L, WDR12, ALS2CR8 | likely benign | No DGV reports, only large deletions reported in DECIPHER; ICA1L islet cell autoantigen 1 like; the WDR12 encoded protein is a component of a nucleolar protein complex that affects maturation of the large ribosomal subunit (NCBI GeneID 55759), highly expressed in mammary tissue and EBV transformed lymphocytes; amyotrophic lateral sclerosis 2 (juvenile) chromosome region, candidate 8, associated with early-onset myocardial infarction and important for rat heart function[^14^](#_ENREF_14) , no second variant affecting function in WES, good coverage The patient has biallelic *SPATA5* variants explaining the phenotype, |
| 47651  Decipher 370082 | M | chr4:123952079-124003383 | 1 | 51 | Mat | **SPATA5** | **recessive allele** | AR Epilepsy, hearing loss and mental retardation syndrome (MIM #616577) WES revealed variant in trans in the *SPATA5* gene |
| 47651  Decipher 370082 | M | chr17:10404449-10432638 | 3 | 28 | Mat | MYH1, MYH2 | benign | Duplication that partially affects the involved genes; DGV reports: one larger duplication including both genes, smaller duplications partially affecting only one of the two genes  The patient has biallelic *SPATA5* variants explaining the phenotype |
| 72984  Decipher 370083 | M | chr20:62523752-62535485 | 3 | 12 | Mat | TPD52L2, DNAJC5 | benign | Variants in DNAJC5 are known to cause AD Ceroid Lipofuscinosis, Neuronal 4B (MIM #162350) would be late onset and rapidly progressing[^15^](#_ENREF_15); first exon (NG_029805.1) is non-coding CNV analysis of WES data confirms duplication in patient and his mother |
| 71118  Decipher 370074 | M | chr9:99081511-99091669 | 1 | 10 | Pat | SLC35D2 | VOUS | Member of the SLC35 nucleotide sugar transporter family, localized in the Golgi membrane, it may be the ortholog of yeast frc transporter, which is involved in the Notch signaling[^16^](#_ENREF_16) WES revealed no second variant affecting function |

Table S3. Disease-associated sequence and copy number variants in established EE/ID genes or regions

| **Patient ID^a^**  **Decipher or LOVD ID** | **Sex** | **Gene / region** | **Inheritance** | **Type of variant** | **Genome position (hg19) and HGVS variant nomenclature** | **Predicted protein effect** | **Known variant in HGMD (HGMD accession number) / ExAC allele frequency / references** | **Known disorder**  **(MIM number)** |
| --- | --- | --- | --- | --- | --- | --- | --- | --- |
| 72356  Decipher 370071 | F | **1p36.23-pter** | AD, DN | CNV (7.7 Mb) | chr1:g.(0_849466)_(7666975_7683885)del | Loss (62 OMIM genes, total of 118) | Reviewed by Jordan et al., 2015[^17^](#_ENREF_17) | 1p36 deletion syndrome (607872) |
| 71592  Decipher 370076 | M | **22q11.22 -q11.23** | AD, DN | Heterozygous CNV (2 Mb) | chr22:g.(22953405_22953514)_(25026857_25027086)dup | Gain (25 OMIM genes, total of 45) | Reported in a patient with developmental delay, hyperactivity, epilepsy, and distinctive facial features[^18^](#_ENREF_18) | 22q11.22q11.23 recurrent microduplication |
| 69986  LOVD 176985 | F | **ACO2** | AR, CH | Heterozygous missense | chr22:41922363  NM_001098.2:c.1859G>A | p.(Gly620Asp) | unreported in HGMD  unreported in ExAC | Infantile cerebellar-retinal degeneration (614559) |
|  |  |  |  | Heterozygous missense | chr22:41923386  NM_001098.2:c.2048G>T | p.(Gly683Val) | unreported in HGMD  unreported in ExAC |  |
| 70757  LOVD 176997 | F | **AP4S1** | AR, HO | Homozygous splice site | chr14:31535543-31535546NG_031913.1(NM_007077.4):c.138+3_138+6del | p.(?) | Seizures, fever-sensitive, developmental delay & spastic paraplegia[^19^](#_ENREF_19)  (HGMD CD152095)  unreported in ExAC | Spastic paraplegia 52, autosomal recessive (614067) |
| 72892  LOVD 177002 | M | **ARX** | XL-R | Hemizygous missense | chrX:25031055  NM_139058.2:c.1057C>T | p.(Pro353Ser)  see also supplemental table S6 | unreported in HGMD  p.(Pro353Arg) (Lissencephaly, X-linked, with abnormal genitalia[^20^](#_ENREF_20))  p.(Pro353Leu) (Mental retardation and epilepsy[^21^](#_ENREF_21))  unreported in ExAC | Epileptic encephalopathy, early infantile, 1 (308350) |
| 73311 /  76351  LOVD 177003 | both M | **BRAT1** | AR, CH | Heterozygous frameshift | chr7:2578041-2578044  NM_152743.3:c.2125_2128del | p.(Phe709Thrfs*17) | Reported in 2 siblings with developmental delay, microcephaly, refractory seizures and infant death[^22^](#_ENREF_22)  (HGMD CD1613285)  ExAC: 0.0025% | Rigidity and multifocal seizure syndrome, lethal neonatal (614498) |
|  |  |  |  | Heterozygous frameshift | chr7:2583389  NM_152743.3:c.638dup | p.(Val214Glyfs*189) | Reported 5 times in Lethal neonatal rigidity and seizure syndrome[^23-27^](#_ENREF_23)  (HGMD CI121212)  unreported in ExAC |  |
| 72128  Decipher 370072 | F | **CDKL5** | XL-D, DN | Heterozygous mosaic CNV (25 Kb) | chrX:g.(18592712_18592741)_(18617503_18617862)del | Loss (CDKL5) | - | Epileptic encephalopathy, early infantile, 2 (300672) |
| 72404  LOVD 177004 | M | **CDKL5** | XL-D, DN | Hemizygous splice site | chrX:18593613_18593616  NG_008475.1(NM_003159.2):c.282+3_282+6del | p.(?) | CDKL5 disorder[^28^](#_ENREF_28)  (HGMD CD139237)  unreported in ExAC | Epileptic encephalopathy, early infantile, 2 (300672) |
| 73324  LOVD 177005 | F | **GABRB2** | AD, DN | Heterozygous missense | chr5:160761872  NM_021911.2:c.719G>C | p.(Arg240Thr)  see also supplemental table S6 | unreported in HGMD  unreported in ExAC | Novel disease gene previously reported in a few patients, only. De novo missense variants in this gene have been reported in one patient with ID & epilepsy[^29^](#_ENREF_29); in 2 patients with epilepsy[^30^](#_ENREF_30) and eleven patients with mostly ID and refractory seizures[^31^](#_ENREF_31). |
| 73214  LOVD 176996 | F | **KCNQ2** | AD, DN | Heterozygous missense | chr20:62073835  NM_172107.2:c.740C>T | p.(Ser247Leu) | unreported in HGMD  p.(Ser247*) reported in benign infantile epilepsy[^32^](#_ENREF_32)  p.(Ser247Trp) reported in epilepsy with regression and EE, inherited from mother with milder phenotype[^33^](#_ENREF_33)  unreported in ExAC | Epileptic encephalopathy, early infantile, 7 (613720) |
| 50126  Decipher 370070 | M | **MBD5** | AD, DN | Heterozygous CNV (197 Kb) | chr2:g.(148757084_148762374)_(148959158_148960882)del | Loss (MBD5,ORC4) | - | Mental retardation, autosomal dominant 1 (156200) |
| 71693  LOVD 177006 | M | **POLG** | AR, CH  Likewise affected sister also CH | Heterozygous missense | chr15:89865023  NM_002693.2:c.2542G>A | p.(Gly848Ser) | Multiple times reported in Alpers syndrome with intractable seizures, sensory ataxic neuropathy with dysarthria and ophthalmoparesis, etc  (HGMD CM021662)  ExAC (European): 0.02%  ExAC (global): 0.016% | *POLG*-related disorder[^34^](#_ENREF_34) |
|  |  |  |  | Heterozygous missense | chr15:89873343  NM_002693.2:c.824G>A | p.(Arg275Gln) | Multiple sclerosis-like features[^35^](#_ENREF_35) and sensory ataxic neuropathy with ophthalmoparesis^[36](#_ENREF_36)^  (HGMD CM105918)  unreported in ExAC |  |
| 69937  LOVD 177008 | M | **PRUNE1** | AR, HO | Homozygous missense | chr1:150991126  NM_021222.2:c.316G>A | p.(Asp106Asn)  see also supplemental table S6 | Described by Karaca et al., 2015 in 2 patients from different families with microcephaly, cortical and cerebellar atrophy[^37^](#_ENREF_37)  (HGMD CM1512541)  ExAC : 0.004% | Novel gene for Neurodevelopmental disorder with microcephaly, hypotonia, and variable brain anomalies (617481) previously reported in a few patients, only. |
| 34124  LOVD 177009 | M | **SCN1A** | AD, DN | Heterozygous missense | chr2:166848437  NM_001165963.1:c.5348C>T | p.(Ala1783Val) | Myoclonic epilepsy of infancy[^38^](#_ENREF_38)^,^[^39^](#_ENREF_39)  (HGMD CM076501)  unreported in ExAC | Dravet syndrome (607208) |
| 47970  LOVD 177010 | M | **SCN1A** | AD, DN | Heterozygous frameshift | chr2:166850754  NM_001165963.1:c.4754del | p.(Thr1585Metfs*6) | unreported in HGMD  unreported in ExAC | Dravet syndrome (607208) |
| 75143  LOVD 177011 | F | **SCN1A** | AD, DN | Heterozygous frameshift | chr2:166904165  NM_001165963.1:c.1142del | p.(Gln381Argfs*10) | unreported in HGMD | Dravet syndrome (607208) |
| 42680  LOVD 177012 | M | **SCN2A** | AD, DN | Heterozygous missense | chr2:166245724  NM_021007.2:c.5408A>G | p.(Glu1803Gly) | unreported in HGMD  unreported in ExAC | Epileptic encephalopathy, early infantile, 11 (613721) |
| 43092  LOVD 177013 | M | **SCN8A** | AD, DN | Heterozygous missense | chr12:52200885  NM_014191.3:c.5615G>A | p.(Arg1872Gln) | Epileptic encephalopathy[^40-42^](#_ENREF_40)  (HGMD CM150892)  unreported in ExAC | Epileptic encephalopathy, early infantile, 13 (614558) |
| 72555 / 72719  LOVD 177014 | both M | **SMS** | XL-R, maternal, DN in mother | Hemizygous missense | chrX:21995237  NM_004595.4:c.388C>T | p.(Arg130Cys) | unreported in HGMD  unreported in ExAC | Snyder-Robinson syndrome (182290); our patients are described in great detail in Abela et al., 2016[^43^](#_ENREF_43) |
| 47651  LOVD 177015 | M | **SPATA5** | AR, CH | CNV (51 Kb) | chr4:g.(123951799_123952079)_(124003383_124003384)del | Loss | - | Epilepsy, hearing loss, and mental retardation  syndrome (616577) |
|  |  |  |  | In-frame deletion | chr4:123855735-123855737  NM_145207.2:c.989_991del | p.(Thr330del) | Reported in microcephaly, intellectual disability, seizures & hearing loss[^44^](#_ENREF_44)^,^[^45^](#_ENREF_45)  (HGMD CD1514219)  ExAC (European): 0.023%  ExAC (global): 0.013% |  |
| 73068  LOVD 177016 | M | **SPATA5** | AR, CH | Heterozygous missense, DN, paternal allele | chr4:124177219  NM_145207.2:c.2389C>G | p.(Pro797Ala) | unreported in HGMD  unreported in ExAC | Epilepsy, hearing loss, and mental retardation  syndrome (616577) |
|  |  |  |  | Heterozygous loss of the primary start codon, inherited from the mother | chr4:123844298  NM_145207.2:c.1A>C | p.(Met1?) | unreported in HGMD  ExAC: 0.005% |  |
| 52236  LOVD 181099 | F | **STXBP1** | AD, DN | Heterozygous splice site | chr9:130422393  NG_016623.1(NM_003165.3):c.325+6T>C | p.(?) | unreported in HGMD  c.325+4A>G and c.325+5G>A are reported 4 times in epileptic encephalopathy[^46^](#_ENREF_46)^,^[^47^](#_ENREF_47)^,^[^48^](#_ENREF_48)^,^[^49^](#_ENREF_49)  unreported in ExAC | Epileptic encephalopathy, early infantile, 4 (612164) |
| 73805  LOVD 177023 | M | **STXBP1** | AD, DN | Heterozygous missense | chr9:130438941  NM_003165.3:c.1268T>C | p.(Leu423Pro) | unreported in HGMD  unreported in ExAC | Epileptic encephalopathy, early infantile, 4 (612164) |
| 72943  LOVD 177024 | M | **SZT2** | AR | Heterozygous frameshift | chr1:43885570  NM_015284.3:c.1045del | p.(Ser349Profs*9) | unreported in HGMD  unreported in ExAC | Epileptic encephalopathy, early infantile, 18  (615476) |
|  |  |  |  | DN, heterozygous missense | chr1:43888272  NM_015284.3:c.1891G>A | p.(Glu631Lys) | unreported in HGMD  unreported in ExAC |  |
| 71412  Decipher 370084 | F | **15q11.2 - q13.11 (UBE3A)** | AD, DN, maternal allele | Heterozygous, CNV (4.9 Mb) | chr15:g.(23620154_23620191)_(28545355_28545445)del | Loss (19 OMIM genes out of a total of 105) | Recurrent deletion | Angelman Syndrome (105830) |
| 49635  Decipher 370081 | M | **UBE3A** | AD, DN, maternal allele | Heterozygous, CNV (134 Kb) | chr15:g.(25583244_25583408)_(25717757_ 25717851)del | Loss (UBE3A) | Overlapping 270 kb deletion in epileptic encephalopathy [^50^](#_ENREF_50) | Angelman Syndrome (105830) |
| Note: ^a^ two ID numbers indicate affected siblings with the same phenotype | | | | | | | | |

Table S4. ACMG variant classification of disease-associated variants in established genes causing epileptic encephalopathy

Genes highlighted in beige are recently established disease genes described previously in few patients only (details on variant nomenclature see table S3)

| **Patient ID** | **Gene** | **Variant call** | **Predicted protein change** | **Allele Frequency (ExAC)** | **Genotype** | **Inheritance** | **Overall ACMG classification** | **Evidence of pathogenicity** |
| --- | --- | --- | --- | --- | --- | --- | --- | --- |
|  |  |  |  |  |  |  |  |  |
| 73214 | **KCNQ2** | c.740C>T | p.(Ser247Lys) | 0 | Heterozygous | AD, DN | **Pathogenic** | PS2, PM1, PM2, PM5, PP3 |
| 72555/ 72719 | **SMS** | c.388C>T | p.(Arg130Cys) | 0 | Hemizygous | XL-R | **Pathogenic** | PS3, PM1, PM2, PP1, PP3 |
| 72404 | **CDKL5** | c.282+3_282+6 | p.(?) | 0 | Hemizygous | XL-D, DN | **Pathogenic** | PS1, PS2, PM2, PP3 |
| 70757 | **AP4S1** | c.138+3_138+6del | p.(?) | 0 | Homozygous | AR | **Pathogenic** | PS1, PS3, PM2, PP1, PP3 |
| 47970 | **SCN1A** | c.4754del | p.(Thr1585Metfs*6) | 0 | Heterozygous | AD, DN | **Pathogenic** | PVS1, PS2, PM2 |
| 73068 | **SPATA5** | c.2389C>G | p.(Pro797Ala) | 0 | Compound Heterozygous | AR | **Likely pathogenic** | PS2, PM1, PM2, PP3 |
|  | **SPATA5** | c.1A>C | p.(M1?) | 4.945E-05 |  |  | **Likely pathogenic** | PVS1, PM2 |
| 47651 | **SPATA5** | c.989_991del | p.(Thr330del) | 0.0001334 | Compound Heterozygous | AR | **Likely pathogenic** | PS1, PM2, PM4 |
|  | **SPATA5** | 51 kb del |  |  |  |  | **Likely pathogenic** | PVS1, PM2 |
| 52236 | **STXBP1** | c.325+6T>C | p.(?) | 0 | Heterozygous | AD, DN | **Likely pathogenic** | PS2, PM2, PP3 |
| 69986 | **ACO2** | c.1859G>A | p.(Gly620Asp) | 0 | Compound Heterozygous | AR | **Likely pathogenic** | PS3, PM2, PP3 |
|  | **ACO2** | c.2048G>T | p.(Gly683Val) | 0 |  |  | **Likely pathogenic** | PS3, PM2, PP3 |
| 73311/ 76351 | **BRAT1** | c.2125_2128del | p.(Phe709Thrfs*17) | 0 | Compound Heterozygous | AR | **Pathogenic** | PVS1, PS1, PM2, PM3, PP1 |
|  | **BRAT1** | c.638dup | p.(Val214Glyfs*189) | 0 |  |  | **Pathogenic** | PVS1, PS1, PM2, PM3, PP1 |
| 43092 | **SCN8A** | c.5615G>A | p.(Arg1872Gln) | 0 | Heterozygous | AD, DN | **Pathogenic** | PS1, PS2, PS3, PM2, PP3 |
| 34124 | **SCN1A** | c.5348C>T | p.(Ala1783Val) | 0 | Heterozygous | AD, DN | **Pathogenic** | PS1, PS2, PM1, PM2, PP3 |
| 73805 | **STXBP1** | c.1268T>C | p.(Lys423Pro) | 0 | Heterozygous | AD, DN | **Likely pathogenic** | PS2, PM2, PP3 |
| 72892 | **ARX** | c.1057C>T | p.(Pro353Ser) | 0 | Hemizygous | XL-R | **Likely pathogenic** | PM1, PM2, PM5, PP3 |
| 42680 | **SCN2A** | c.5408A>G | p.(Glu1803Gly) | 0 | Heterozygous | AD, DN | **Likely pathogenic** | PS2, PM2, PP3 |
| 73324 | **GABRB2** | c.719G>C | p.(Arg240RThr) | 0 | Heterozygous | AD, DN | **Likely pathogenic** | PS2, PM1, PM2, PP3 |
| 71693 | **POLG** | c.2542G>A | p.(Gly848Ser) | 0.0001566 | Compound Heterozygous | AR | **Pathogenic** | PS1, PS3, PM2, PP1, PP3 |
|  | **POLG** | c.824G>A | p.(Arg275Gln) | 0 |  |  | **Pathogenic** | PS1, PM2, PM3, PP1, PP3 |
| 75143 | **SCN1A** | c.1142del | p.(Gln381Argfs*10) | 0 | Heterozygous | AD, DN | **Pathogenic** | PVS1, PS2, PM2 |
| 72943 | **SZT2** | c.1045del | p.(Ser349Profs*9) | 0 | Compound Heterozygous | AR | **Likely pathogenic** | PVS1, PM2 |
|  | **SZT2** | c.1891G>A | p.(Glu631Lys) | 0 |  |  | **Likely pathogenic** | PS2, PM2, PP3 |
| 69937 | **PRUNE1** | c.316G>A | p.(Asp106Asn) | 4.141E-05 | Homozygous | AR | **Likely pathogenic** | PS1, PM1, PM2, PP3 |

**Key for ACMG categories:**

**Evidence of pathogenicity**

Very Strong - PVS1 Null variant (nonsense, frameshift, canonical ±1 or 2 splice sites, initiation codon, single or multi-exon deletion) in a gene where LOF is a known mechanism of disease

Strong - PS1 Same amino acid change as a previously established pathogenic variant regardless of nucleotide change

Strong - PS2 De novo (both maternity and paternity confirmed) in a patient with the disease and no family history

Strong - PS3 Well-established in vitro or in vivo functional studies supportive of a damaging effect on the gene or gene product

Strong - PS4 The prevalence of the variant in affected individuals is significantly increased compared with the prevalence in controls

Moderate - PM1 Located in a mutational hot spot and/or critical and well-established functional domain (e.g., active site of an enzyme) without benign variation

Moderate - PM2 Absent from controls (or at extremely low frequency if recessive) (Table 6) in Exome Sequencing Project, 1000 Genomes Project, or Exome Aggregation Consortium

Moderate - PM3 For recessive disorders, detected in trans with a pathogenic variant

Moderate - PM4 Protein length changes as a result of in-frame deletions/insertions in a non-repeat region or stop-loss variants

Moderate - PM5 Novel missense change at an amino acid residue where a different missense change determined to be pathogenic has been seen before

Moderate - PM6 Assumed de novo, but without confirmation of paternity and maternity

Supporting - PP1 Co-segregation with disease in multiple affected family members in a gene definitively known to cause the disease

Supporting - PP2 Missense variant in a gene that has a low rate of benign missense variation and in which missense variants are a common mechanism of disease

Supporting - PP3 Multiple lines of computational evidence support a deleterious effect on the gene or gene product (conservation, evolutionary, splicing impact, etc.)

Supporting - PP4 Patient’s phenotype or family history is highly specific for a disease with a single genetic etiology

Supporting - PP5 Reputable source recently reports variant as pathogenic, but the evidence is not available to the laboratory to perform an independent evaluation

**Evidence of benign impact**

Stand Alone - BA1 Allele frequency is >5% in Exome Sequencing Project, 1000 Genomes Project, or Exome Aggregation Consortium

Strong - BS1 Allele frequency is greater than expected for disorder

Strong - BS2 Observed in a healthy adult individual for a recessive (homozygous), dominant (heterozygous), or X-linked (hemizygous) disorder, with full penetrance expected at an early age

Strong - BS3 Well-establish in vitro or in vivo functional studies show no damaging effect on protein function or splicing

Strong - BS4 Lack of segregation in affected members of a family

Supporting - BP4 Multiple lines of computational evidence suggest no impact on gene or gene product (conservation, evolutionary, splicing impact, etc.)

Supporting - BP6 Reputable source recently reports variant as benign, but the evidence is not available to the laboratory to perform an independent evaluation

Table S5. Details on variants in recurrent candidate genes for neurodevelopmental disorders

| **Patient ID**  **LOVD ID** | **Sex** | **Gene / region** | **Inheritance pattern** | **Type of variant (inheritance)** | **Genome position (hg19) and HGVS variant nomenclature** | **Supporting evidence**  **(information copied from UCSC and Gene Card or taken from cited references)**  **Details on structural modeling and functional evidence for selected variants in supplemental table S6** | **ExAC**  **Gene LoF constraint information**  **Allele count/ Allele number/**  **Homozygotes**  **dbSNP reference cluster ID** |
| --- | --- | --- | --- | --- | --- | --- | --- |
| 33386  LOVD 177064 | F | GTF3C3 | CH | Heterozygous missense (PAT)  Present in healthy sister | chr2:197631408  NM_012086.4:c.2419C>T  p.(Arg807Cys) | 1 of 2 RefSeq isoforms affected  Subunit of the DNA-binding subcomplex (TFIIIC2) of transcription factor IIIC (TFIIIC), ageneral transcription factor involved in RNA polymerase III-mediated transcription. GTF3C3 interacts with BRF1[^51^](#_ENREF_51)^,^[^52^](#_ENREF_52) (a subunit of TFIIIB), which is involved in Cerebellofaciodental syndrome (AR, delayed development, intellectual disability, abnormal facial and dental findings, and cerebellar hypoplasia). [^53^](#_ENREF_53)^,^[^54^](#_ENREF_54) This patient also has marked cerebellar atrophy and large incisors.  Reuter et al. (2017) reported a homozygous missense variant in *GTF3C3* in two affected sisters with a phenotype including mild ID, seizures and dysmorphisms and classified it as a moderately confident candidate gene.[^55^](#_ENREF_55) Anazi et al. (2017) found a homozygous splice site variant which was shown to cause skipping of exon 10 and parts of exon 11 (in-frame deletion) in a patient with profound microcephaly, characteristic facial appearance and failure to thrive.[^56^](#_ENREF_56)  Expressed in cerebral cortex (The Human Protein Atlas)  ALAMUT predictions: deleterious  CADD score: 22.4  Structural modeling is inconclusive, see also supplemental table S6 | LoF expected: 35  LoF observed: 15  not reported in ExAC  not in dbSNP |
|  |  |  |  | Heterozygous missense(MAT)  Not present in healthy sister | chr2:197656093  NM_012086.4:c.503C>T  p.(Ala168Val) | All RefSeq isoforms affected  ALAMUT predictions: deleterious  CADD score: 32  Structural modeling is inconclusive, see also supplemental table S6 | not reported in ExAC  not in dbSNP |
| 69314  LOVD 177065 | F | PIK3AP1 | DN | Heterozygous missense | chr10:98411052  NM_152309.2:c.941T>C  p.(Leu314Pro) | De novo missense variants in PIK3AP1 have been described in one patient with intractable infantile spasms, developmental delay and hypotonia and in one patient with infantile spasms responsive to treatment and a normal development.[^57^](#_ENREF_57)  ALAMUT predictions: deleterious  The variant is located in DBB domain, which is required in other proteins to mediate protein-protein interaction (see also supplemental table S6) | LoF expected: 27.0  LoF observed: 1  not reported in ExAC  not in dbSNP |
| 76366  LOVD 177066 | M | UFC1 | HO | Homozygous missense  (parents heterozygous) | chr1:161123855  NM_016406.3:c.68G>A  p.(Arg23Gln) | All RefSeq isoforms affected  Anazi et al., 2017 first reported this gene as candidate for recessive ID. The affected patient (failure to thrive, brain atrophy, global developmental delay, hypotonia) carries a missense homozygous variant in UFC1 with predicted effect on Ufm1-modification function.[^58^](#_ENREF_58) Further five patients from two families carrying the same recurrent missense variant in a homozygous state share a phenotype of microcephaly, short stature, global developmental delay, seizures, and failure to thrive.[^59^](#_ENREF_59)  UFC1 is, together with UBA5, part of the UFM1 ufmylation pathway. Variants in UBA5 and UFM1 have also been described in patients with early-onset encephalopathy.[^59-63^](#_ENREF_59)  GO annotations related to this gene include *UFM1 transferase activity*.  Expressed in cerebellum, hippocampus, cerebral cortex and lateral ventricle (The Human Protein Atlas)  ALAMUT predictions: deleterious  CADD score: 22.3  Mutation modeling is inconclusive, see also supplemental table S6 | LoF expected: 12.7  LoF observed: 4  not reported in ExAC  not in dbSNP |
| 73450  LOVD 177082 | M | WRAP53 | AR, CH | Heterozygous missense (MAT) | chr17:7605740  NM_018081.2:c.1034A>G p.(Tyr345Cys) | All RefSeq isoforms affected  Autosomal-recessive variants in *WRAP53* have been reported as disease cause in two unrelated patients with the classical phenotype of Dyskeratosis congenita (DC) (MIM 613988),[^64^](#_ENREF_64) supported by functional studies of the detected variants.[^64^](#_ENREF_64)^,^[^65^](#_ENREF_65) As previously described in other DC subtypes,[^66^](#_ENREF_66) our patient shows a delayed development, intrauterine growth retardation, cerebellar hypoplasia, microcephaly and thrombocytopenia. He also has absence seizures, which is an extension of the phenotypic spectrum of DC reported here for the first time. Interestingly, seizures have been reported before in a DC mouse model in a part of the mice.[^67^](#_ENREF_67)  ALAMUT predictions: deleterious  The variant is located in a WD repeat and might be part of regions mediating protein-protein interactions (see also supplemental table S6). | LoF expected: 24.6  LoF observed: 5  3/120960/0  rs778192810 |
|  |  |  |  | Heterozygous missense (PAT) | chr17:7606345  NM_018081.2:c.1303G>A  p.(Gly435Arg) | All RefSeq isoforms affected  Zhong et al., 2011 reported 1 patient with Dyskeratosis congenita and performed functional studies[^64^](#_ENREF_64)  Clinvar: [RCV000034151.7](https://www.ncbi.nlm.nih.gov/clinvar/RCV000034151.7) (Pathogenic - Dyskeratosis congenita, autosomal recessive, 3).  ALAMUT predictions: deleterious  This variant disrupts telomerase localization to Cajal bodies resulting in misdirection of telomerase RNA to nucleoli (see also supplemental table S6). | 1/121300/0  rs281865550 |

ALAMUT predictions include SIFT [^68^](#_ENREF_68), MutationTaster [^69^](#_ENREF_69) and PolyPhen-2 [^70^](#_ENREF_70): deleterious (at least 2 of 3), moderately deleterious (1 of 3), tolerated (all 3 predictions benign)

**Table S6. Structural modeling of and functional information on selected variants**

**Color code of variant classification deriving from molecular modelling only (no color in variants without structural information):**

**Variant deleterious** (strong effect on protein structure deduced from molecular modeling)

**variant likely deleterious** (effect on protein structure deduced from molecular modeling)

**variant located in a structurally/functionally important domain** (but no statement on a residue-level possible)

**41637_ADCK3: p.(Arg353Cys)**

- Arg353 forms salt bridges with Glu432 and Glu446
- The known 3D-structure of ADCK3 (PDB: 4PED; doi: 10.1016/j.molcel.2014.11.002) is shown below. Residues involved in salt-bridge formation are depicted as sticks and interactions are shown as dotted lines. These stabilizing salt-bridges would be lost after mutation to cysteine

**
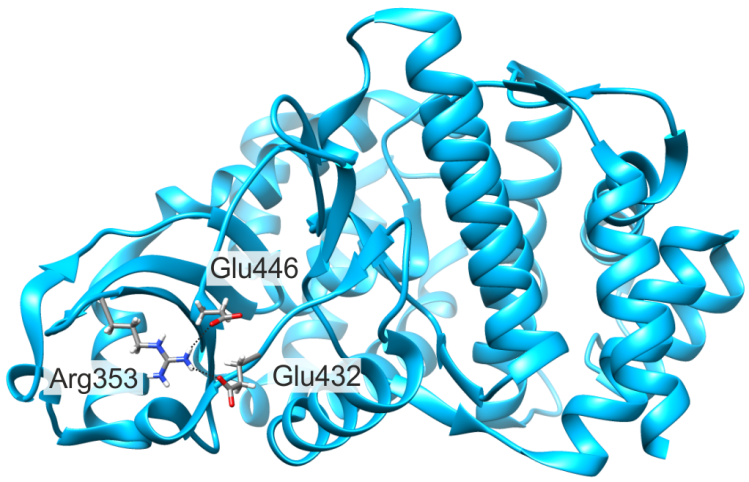
**


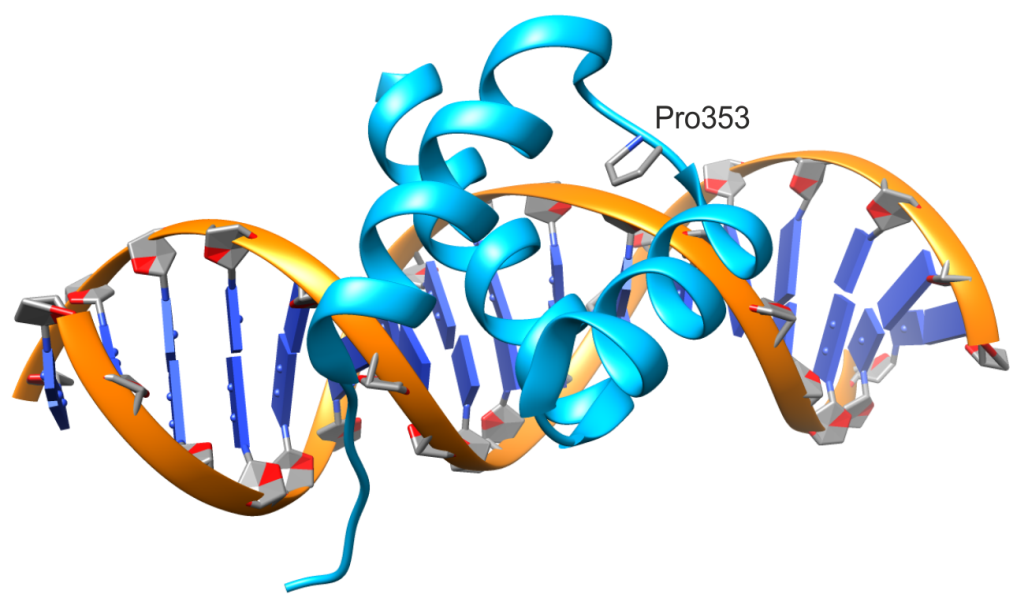
**72892 _ARX: p.(Pro353Ser)**

- Pro353 is in the conserved DNA-binding homeobox
- Other variants of position 353 are already described as pathologic:
  - to arginine (Lissencephaly, X-linked 2 (LISX2))
    - doi: 10.1002/humu.10310;
  - to leucine (corresponds to variant rs28936074; Epileptic encephalopathy, early infantile, 1 (EIEE1))
    - doi: 10.1038/ng862;
    - doi: 10.1212/WNL.59.3.348;
- Homologous protein 3D- structures are available (PDB: 3A01 (doi: 10.1038/emboj.2010.53))
  - Pro353 is in a loop between two helices, not directly involved in DNA-binding (see picture on the right)
- Mutation of Pro353 to serine: Homology modeling suggests that a replacement of the rigid hydrophobic proline by hydrophilic serine destabilizes the respective region

**70757_FKTN: p.(Val8Met) – likely tolerated**

- helical transmembrane region; signal-anchor for type II membrane protein
- only very small effect of the exchange on the properties of the TM-helix predicted
- no 3D structural data are available

**73324_GABRB2: p.(Arg240Thr)**

- Arg240 is highly conserved and in the extracellular part of the protein
- In the highly homologous structure of the GABA(A)R-beta3 homopentamer (PDB: 4COF; doi: 10.1038/nature13293; 91% sequence identity) this arginine interacts with an aspartate, glutamate and glutamine (all interacting residues are conserved in GABRB2). These interactions will no longer be possible after mutation to threonine
- The figure below shows the location of Arg240 in the pentameric structure (left panel) and the interactions of Arg240 are shown as an enlargement on the right side. Interactions are shown as dotted lines.

**
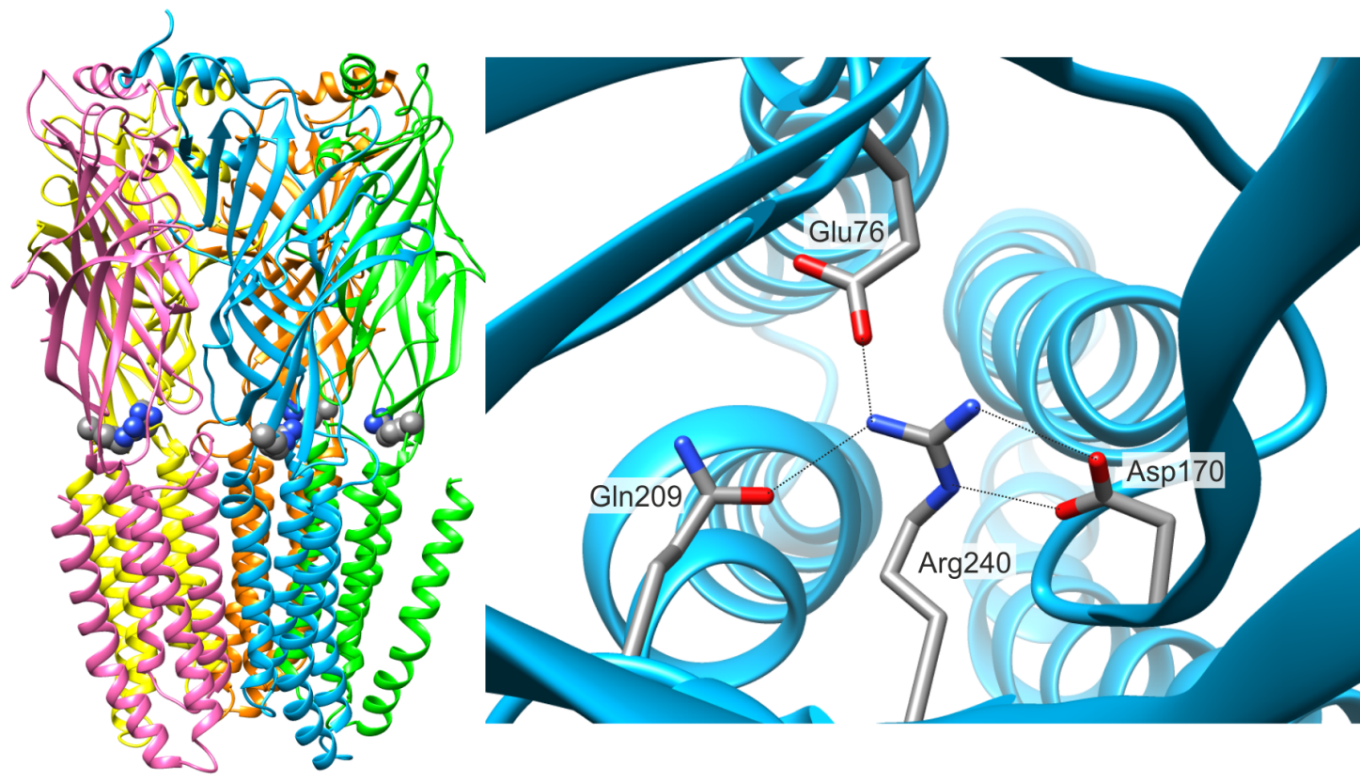
**

**33386_GTF3C3: p.(Ala168Val) and p.(Arg807Cys)**

- Ala168
  - conserved alanine side chain has no important contacts to neighboring residues in the homologous structures
  - in an homologous structure, a mutation to valine doesn’t cause clashes with neighboring residues suggesting that it might be tolerated
- Arg807
  - not conserved in homologous structures
  - residues at this position do not make specific contacts in homologous structures
- However, an effect of these variants on the interaction with other protein domains or interactions partners cannot be excluded. These interactions cannot be modeled due to the lack of structural information.

**71693_KCNQ2: p.(Arg647Gln)**

- In the cytoplasmic region of the protein
- No 3D structural data available

**73790 _KDM5C: p.(Arg787Trp)**

- Not conserved in the structure of homologous proteins - in addition, the respective sequence stretch lacks in in the known crystal structure (PDB:5FWJ; doi: 10.1038/nchembio.2087) indicating that it is dispensable for the formation of the 3D structure

**69314_PIK3AP1: p.(Leu314Pro)**

- Leu314 is predicted to be in the DBB domain, which is required in other proteins to mediate protein-protein interaction (PROSITE; Reference indicating that this domain may have a more general role in mediating protein-protein interactions doi: 10.1016/S0022-2836(03)00489-3)
- No 3D structural data available

**69937_PRUNE1: p.(Asp106Asn)**

- PRUNE1 is a phosphodiesterase (PDE) of unknown3D structure belonging to the DHH superfamily
- Asp106 is part of the conserved DHH motif (Asp-His-His; residues 106 – 108)
- the DHH motif is in involved in metal ion binding and catalysis
- An Asp106Ala variant was shown to lead to a reduced activity with a three-fold smaller K_cat_ compared to the wildtype (Tammenkoski et al. Biochemistry, 2008, 47 (36), pp 9707–9713; doi: 10.1021/bi8010847)

**71592_RAF1: p.(Ile474Val)**

- Crystal structure is available (PDB: 3OMV; doi: 10.1038/nature08833)
- Ile474 is in the protein kinase domain, located close to the ATP-binding site
- Figure below, left panel: In the wildtype, the Cδ-methyl group of Ile474 (orange balls) interacts with the methyl groups of Val537 (magenta balls).
- Figure below, right panel: Val474 lacks the Cδ-methyl group and consequently shows no interactions with Val537, which is expected to destabilize the enzyme

**
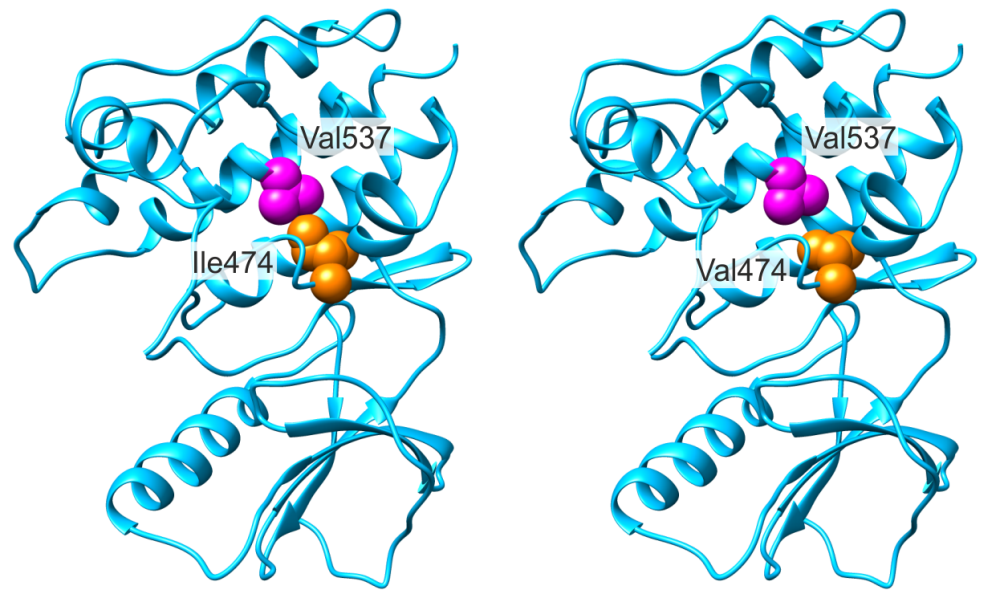
**

**73134 _SCN1A: p.(Arg542Gln)**

- Arg542 is in a cytoplasmic region of the protein
- No 3D structural data are available

**73068_SPATA5: p.(Thr330del)**

- Thr330 is located in the N-terminal proximity of the globular AAA ATPase domain (doi: 10.1186/s13023-016-0509-9)
- No detailed modeling possible, because no proper structural templates available

**76366_UFC1: p.(Arg23Gln)**

- different 3D structures are available PDBs: 2K07 (doi: 10.1007/s10969-008-9054-7), 2Z6O and 2Z6P (doi: 10.1016/j.bbrc.2007.08.129), 3EVX (doi: 10.1007/s10969-008-9054-7)): arginine side chain without important contacts to other residues of the protein, at the surface of the protein, however disturbance of interactions with other proteins and molecules cannot be excluded

**73450_WRAP53: p.(Tyr345Cys) and p.(Gly435Arg)**

- Only structural data from homologous proteins are available
- Homologous residues are located at the surface of the protein in the WD repeats and might be part of regions mediating protein-protein interactions. However, no modelling is possible, since the mode of ligand binding differs widely in the WD domain family.
- Gly435 variant (rs281865550) Gly → Arg in DKCB3; disrupts telomerase localization to Cajal bodies resulting in misdirection of telomerase RNA to nucleoli (doi: 10.1101/gad.2006411).

Table S7. Patients with incidental findings of previously reported or truncating variants in known disease genes (pink – epilepsy genes)

| **Patient**  **ID**  **LOVD or Decipher ID** | **Sex** | **Gene / region** | **Inheritance** | **Genome position (hg19), HGVS variant nomenclature and predicted protein effect** | **Known disorder**  **(OMIM)** | **Known variant**  **(HGMD, dbSNP, ExAC Allele count/Allele number/Homozygotes/Hemizygotes, References, other comments)** | **Variant classification**  **(ACMG standards and guidelines**[**^71^**](#_ENREF_71)**)** |
| --- | --- | --- | --- | --- | --- | --- | --- |
| 72440  LOVD 181150 | M | ABCC2 | CH,  MAT  PAT | chr10:101591889  NG_011798.1(NM_000392.4):c.3258+1G>A  p.(?)  chr10:101578835  NG_011798.1(NM_000392.4):c.2440-11T>C  p.(?) | Dubin-Johnson syndrome (AR) (MIM #237500) | Reported in HGMD as DM (CS132432)  dbSNP: rs762243203  ExAC: 8/121246/0  ALAMUT predictions: loss of splice donor  RT-PCR on blood revealed skipping of exon 23.  Not reported in HGMD  Not in dbSNP or ExAC  ALAMUT predictions: no splice effect  RT-PCR on blood did not show any aberrant splicing. | Heterozygous carrier of pathogenic variant for AR Dubin-Johnson syndrome  likely benign |
| 32546  LOVD 181152 | M | AR | XL, MAT | chrX:66931295  NM_000044.3:c.1937C>A  p.(Ala646Asp) | Androgen insensitivity syndrome (XLR) (MIM **#**300068) | Reported in HGMD (DM) (accession number CM962533)  dbSNP: rs1800053  ExAC: 78/39910/0/20  ALAMUT predictions: moderately deleterious  No other reports; too frequent in general population and 20x hemizygous in ExAC | Likely benign |
| 75605  LOVD 181193 | M | ASB10 | MAT | chr7:150878258  NM_001142459.1:c.872A>G  p.(Asp291Gly) | Glaucoma, primary open angle (MIM **#**603383) | Reported in HGMD (DM?) (CM1210122)  dbSNP: rs781304410  ExAC: 9/115224/0  ALAMUT predictions: moderately deleterious | VOUS |
| 70855  LOVD  181155 | F | ATM | MAT | chr11:108181014  NM_000051.3:c.5890A>G  p.(Lys1964Glu) | Breast cancer, susceptibility to (AD) (MIM **#**114480) | Reported in HGMD (DM?) (CM0910514)  dbSNP: rs201963507  ExAC: 13/121054/0  ALAMUT predictions: moderately deleterious  No second variant found by high-coverage panel sequencing including CNV analysis | VOUS |
| 47651  LOVD 177015 | M | ATM | PAT | chr11:108124761  NM_000051.3:c.2119T>C  p.(Ser707Pro) |  | Reported in HGMD (DFP) (CM013692)  dbSNP: rs4986761  ExAC: 947/119470/7  ALAMUT predictions: benign  Dörk et al., 2001[^72^](#_ENREF_72): In a comparison of clinical characteristics of patients carrying the Ser707Pro substitution with those of non-carriers, the authors didn’t observe differences regarding the age at onset, but found a markedly higher proportion of axillary node-positive patients and a higher proportion of bilateral breast cancer among the Ser707Pro heterozygotes. Altogether, the Ser707Pro substitution was five times more frequent in patients with bilateral breast cancer than in their random sample from the general population | Likely benign |
| 47651  LOVD 177015 | M | BMP15 | XL, MAT | chrX:50658966  NM_005448.2:c.538G>A  p.(Ala180Thr) | Ovarian dysgenesis 2 and premature ovarian failure 4 (MIM **#**300510) | Reported in HGMD (DM?) (CM061654)  dbSNP: rs104894767  ExAC: 920/87533/6/334  ALAMUT predictions: benign  Rossetti et al., 2009 performed functional studies with no deleterious effect[^73^](#_ENREF_73) | Benign |
| 68944  LOVD 181170 | M | SUGCT (C7orf10) | PAT | chr7:40498796  NM_024728.2:c.895C>T  p.(Arg299Trp) | Glutaric aciduria III (AR) (#231690) | Reported in HGMD (DM) (CM085298)  ExAC: 645/114326/7  dbSNP: rs137852860  ALAMUT predictions: moderately deleterious  Furthermore, Calvo et al., 2010 reported 2 likely deleterious missense variants in this gene as candidate for complex I deficiency (macrocephaly with progressive leukodystrophy, nonspecific encephalopathy, hypertrophic cardiomyopathy, myopathy, liver disease, Leigh syndrome)[^74^](#_ENREF_74) | Likely benign |
| 62075  LOVD  181186 | F | CACNA1H | PAT | chr16:1265066  NM_021098.2:c.5024G>A  p.(Arg1675Gln) | Epilepsy, childhood absence, susceptibility to, 6 and  Epilepsy, idiopathic generalized, susceptibility to, 6 (MIM #611942) | Reported in HGMD (DM?) (CM158740)  ExAC: 22/98300/0  dbSNP: rs149367557  ALAMUT predictions: deleterious  Pippucci et al., 2015 report this variant as possibly disease-associated (no functional studies) in epilepsy with auditory features - inherited from the mother with sleep-related bilateral tonic-clonic seizures at 36 years of age. Variants in this gene show partial or lack of segregation[^75^](#_ENREF_75)  Heron et al., 2007 concluded that CACNA1H variants contributed to susceptibility to epilepsy but are not sufficient to cause epilepsy on their own[^76^](#_ENREF_76) | VOUS |
| 43092  LOVD 177013 | M | CHST6 | NA | chr16:75513536  NM_021615.4:c.191C>T  p.(Pro64Leu) | Macular corneal dystrophy (AR) (MIM #217800) | Reported in HGMD (DM) (CM1410040)  Not in dbSNP or ExAC  ALAMUT predictions: deleterious | Heterozygous carrier of likely pathogenic variant for AR Macular corneal dystrophy |
| 69733  LOVD 181161 | M | CLCN1 | CH  MAT  PAT | chr7:143018525  NM_000083.2:c.501C>G  p.(Phe167Leu)  chr7:143043349  NG_009815.1(NM_000083.2):c.2284+5C>T  p.(?) | Myotonia congenita, dominant (AD) (MIM #160800) and  Myotonia congenita, recessive (AR) (MIM #255700) | Reported in HGMD (DM) (CM940283)  dbSNP: rs149729531  ExAC: 132/121392/0  ALAMUT predictions: moderately deleterious  Peddareddygari et al., 2016 report in their patient also multiple episodes of focal seizures[^77^](#_ENREF_77)  Functional studies of this variant referenced in HGMD found no or only a slight effect  Reported in HGMD (DM?) (CS013818)  dbSNP: rs74824159  ExAC: 2172/120482/27  Reported by Sun et al., 2001:no functional studies but not detected in 50 normal controls, segregates in a recessive manner (however, the father has symptoms but is heterozygous for this variant, only)[^78^](#_ENREF_78)  Tincheva et al., 2016 describe a healthy family member with exactly these two variants[^79^](#_ENREF_79) | VOUS  Likely benign |
| 73237  LOVD 181162 | F | CLCN2 | MAT | chr3:184071575  NM_004366.5:c.1730G>A  p.(Arg577Gln) | Epilepsy, idiopathic generalized, susceptibility to, 11 (AD) (MIM **#**607628) | Reported in HGMD (DM) (CM091413)  ExAC: 24/79728/0  dbSNP: rs137852682  ALAMUT predictions: moderately deleterious  Saint-Martin et al., 2009[^80^](#_ENREF_80): The variant was inherited from healthy father in a family with 2 siblings affected. Not present in 535 controls. The genetic data was supported by functional studies on recombinant CLC2 channels, which revealed a faster deactivation, predicting a loss-of-function Arg577Gln mutant; However, the incomplete segregation of the variants among affected members and the transmission by unaffected parents suggests that these CLCN2 variants alone are not sufficient to induce epilepsy. They may instead represent susceptibility factors among other so far undetected genetic alterations in the respective families | VOUS |
| 69937  LOVD 177008 | M | CLCNKB | DN | chr1:16377387  NM_000085.4:c.1071T>A  p.(His357Gln) | Bartter syndrome, type 3 (AR) (MIM #607364) | Reported in HGMD (DM?) (CM005520)  dbSNP: rs201245211  ExAC: 1/119450/0  ALAMUT predictions: benign | VOUS |
| 41637  LOVD 181151 | M | COQ8A (ADCK3) | CH  PAT  MAT | chr1:227170648  NM_020247.4:c.993C>T  p.(Phe331Phe)  chr1: 227170712  NM_020247.4:c.1057C>T  p.(Arg353Cys) | Coenzyme Q10 deficiency, primary, 4 (AR) (MIM **#**612016) | Not reported in HGMD  dbSNP: rs41303129  ExAC:1795/85834/33  ALAMUT predictions: not affecting the splice site  This variant is reported by Lagier-Tourenne et al., 2008 in a patient with Ubiquinone deficiency with cerebellar ataxia. The patient has a compound-heterozygous variant: c.[993C>T];[1645G>A]. The synonymous variant was shown to lead to exon skipping resulting in an in-frame deletion of around 45 amino acids (p.(Lys314_Gln360del)) demonstrated by RT-PCR in lymphoblastoid cell lines and Sanger sequencing[^81^](#_ENREF_81)  **RT-PCR in our patient on RNA extracted from peripheral blood did not show an abnormal transcript and Sanger sequencing of the transcript revealed the variant heterozygously on cDNA level. Therefore our results are not confirming the published data on exon skipping due to this variant**  Not reported in HGMD  dbSNP: rs373083392  ExAC: 11/49890/0  ALAMUT predictions: deleterious  CADD score 30  Structural modeling predicts severe effects for this variant, see also supplemental table S6  Also present in healthy brother, who does not carry the c.993C>T variant | Likely benign  VOUS |
| 59248  LOVD 181164 | F | COL5A1 | HO, parents hetero | chr9:137642654  NM_000093.4:c.1588G>A  p.(Gly530Ser) | Ehlers-Danlos syndrome, classic type (AD) (MIM #130000) | Reported in HGMD (DM?) (CM020927)  Reported in HO status in a patient with mild classical Ehlers-Danlos syndrome[^82^](#_ENREF_82)  dbSNP: rs61735045  ExAC: 4263/119292/103  ALAMUT: moderately deleterious | Likely benign |
| 73068  177016 | M | COL6A3 | DN | chr2:238274523  NM_004369.3:c.5656C>T  p.(Arg1886Cys) | Bethlem myopathy 1 (AD, AR) (MIM #158810) | Reported in HGMD (DM) (CM161508)  dbSNP: rs121908583  ExAC: 10/121008/0  ALAMUT predictions: deleterious  Reported as VOUS by Lévesque et al., 2016 in a patient with limb-girdle muscle weakness[^83^](#_ENREF_83) | VOUS |
| 59248  LOVD 181164 | F | DPM1 | PAT | chr20:49565187  NM_003859.1:c.274C>G  p.(Arg92Gly) | Congenital disorder of glycosylation, type Ie (AR) (MIM #608799) | Reported in HGMD (DM) (CM000008)  dbSNP: rs121908583  ExAC: 4/121412/0  ALAMUT predictions: deleterious  Kim et al., 2000 reported this variant in HO status in 2 patients with congenital disorder of glycosylation, type Ie (AR, developmental delay, hypotonia, seizures, and acquired microcephaly)[^84^](#_ENREF_84)  This variant is predicted to induce a large splicing change by Xiong et al., 2015[^85^](#_ENREF_85) | Heterozygous carrier for AR congenital disorder of glycosylation, type Ie |
| 72555 / 72719  LOVD 177014 | M | FIG4 | PAT | chr6:110098182_110098185  NM_014845.5:c.1808_1811dup  p.(Pro605Glufs*45) | Amyotrophic lateral sclerosis 11 (AD) (MIM #612577)  Charcot-Marie-Tooth disease, type 4J (AR) (MIM #611228)  Yunis-Varon syndrome (AR) (MIM #216340) | Not reported in HGMD  ExAC: 1/121022/0  dbSNP: rs775015373 | Heterozygous carrier of a likely pathogenic variant for AR Charcot-Marie-Tooth disease type 4J  VOUS for Amyotrophic lateral sclerosis 11 |
| 70757  LOVD 176997 | F | FKTN | HO, parents hetero | chr9:108337335  NM_001079802.1:c.22G>A  p.(Val8Met) | Cardiomyopathy, dilated, 1X (AR) (MIM #611615)  Muscular dystrophy-dystroglycanopathy (congenital with brain and eye anomalies), type A, 4 (MIM #253800)  Muscular dystrophy-dystroglycanopathy (congenital without mental retardation), type B, 4 (MIM #613152)  Muscular dystrophy-dystroglycanopathy (limb-girdle), type C, 4 (MIM #611588) | Not in HGMD  dbSNP: rs368981218  ExAC: 5/121290/0  ALAMUT predictions: deleterious  No 3D structure available for modeling; variant is located in helical transmembrane region, but only very small effect of the exchange on the properties of the TM-helix is predicted (table S6 in the supplementary appendix 2)  serum creatine kinase level normal at the age of 4 years | VOUS |
| 56302  LOVD  181169 | M | GALNTL5 | MAT | chr7: 151664484  NM_145292.3:c.153dup  p.(Val52Serfs*23) | No disease reported in OMIM | Not reported in HGMD  dbSNP: rs781551471  ExAC: 5/120588/0 (low quality call)  One frameshift variant reported in Asthenozoospermia with supporting functional studies in a mouse model[^86^](#_ENREF_86) | Pathogenic for Asthenozoospermia (?) |
| 68944  LOVD 181170 | M | GJB2 | CH,  PAT  MAT | chr13:20763686  NM_004004.5:c.35del  p.(Gly12Valfs*2)  chr13:20763620  NM_004004.5:c.101T>C  p.(Met34Thr) | Bart-Pumphrey syndrome (AD) (MIM #149200) and  Deafness, autosomal dominant 3A (AD) (MIM #601544) and  Deafness, autosomal recessive 1A (AR, DD) (MIM #220290) and  Hystrix-like ichthyosis with deafness (AD) (MIM #602540) and  Keratitis-ichthyosis-deafness syndrome (AD) (MIM #148210) and  Keratoderma, palmoplantar, with deafness (AD) (MIM #148350) and  Vohwinkel syndrome (AD) (MIM #124500) | Reported in HGMD (DM) (CD972240)  dbSNP: rs80338939  ExAC: 733/121352/3  [Mahdieh et](http://www.ncbi.nlm.nih.gov/pubmed/?term=Mahdieh%20N%5BAuthor%5D&cauthor=true&cauthor_uid=19925344) al., 2009 studied the frequency of this variant in general population: Mean carrier frequencies of c.35del variant were found to be 1.89, 1.52, 0.64, 1, and 0.64 for European, American, Asian, Ocean, and African populations, respectively[^87^](#_ENREF_87)  Reported in HGMD (DM) (CM970679)  dbSNP: rs35887622  ExAC1032/121354/13  ALAMUT predictions: deleterious  By *in vitro* functional studies in Xenopus oocytes, White et al., 1998 observed a dominant-negative effect of the Met34Thr mutant polypeptide on the intercellular coupling activity of the wildtype GJB2 polypeptide;[^88^](#_ENREF_88) conflicting reports concerning difference of frequency in cases of hearing loss versus controls; Pollak et al., 2007 suggested that the Met34Thr variant is associated with decreased penetrance and causes mild hearing impairment characterized by relatively late onset and progression[^89^](#_ENREF_89) | Carrier for AR non-syndromic deafness  Likely pathogenic for AR late onset mild hearing impairment |
| 73704 / 46917  LOVD 181199 | F | GLI2 | PAT | chr2:121712934  NM_005270.4:c.571G>A  p.(Gly191Arg) | Culler-Jones syndrome (AD) (MIM #615849) and Holoprosencephaly 9 (AD) (MIM #610829) | Reported in HGMD (DM) (CM1514050)  dbSNP: rs202141899  ExAC: 17/118554/0  ALAMUT predictions: deleterious  This variant was reported by Liu et al., 2015 with supporting functional studies in one male patient with sporadic Hirschsprung disease[^90^](#_ENREF_90) | Likely benign |
| 71693  LOVD 177006 | M | KCNQ2 | PAT | chr20:62038676  NM_172107.2:c.1940G>A  p.(Arg647Gln) | Epileptic encephalopathy, early infantile, 7 (AD) (MIM #613720) and  Myokymia (AD) and Seizures, benign neonatal, 1 (AD) (MIM #121200) | Not in HGMD  dbSNP: rs765583552  ExAC: 5/113936/0  Inherited from the healthy father  This patient has CH POLG variants considered as causative; the likewise affected deceased sister does not carry this KCNQ2 variant  ALAMUT predictions: deleterious  CADD score: 27.7  No 3D protein structure available for modeling | Likely benign |
| 59248  LOVD 181164 | F | KCNQ3 | PAT (not affected) | chr8:133146616  NM_004519.3:c.1720C>T  p.(Pro574Ser) | Seizures, benign neonatal, type 2 (AD) (MIM #121201) | Reported in HGMD (DM) (CM083709)  dbSNP: rs74582884  ExAC: 242/121296/1  ALAMUT predictions: deleterious  [Neubauer](http://www.ncbi.nlm.nih.gov/pubmed/?term=Neubauer%20BA%5BAuthor%5D&cauthor=true&cauthor_uid=18625963) et al., 2008 report this variant in 2 unrelated families with Rolandic epilepsy (pedigrees show inheritance from healthy parents). Screening of 455 patients with idiopathic generalized epilepsy revealed another 8 patients with this variant but not present in 454 controls (p= 0.008)[^91^](#_ENREF_91)  Miceli at al., 2009 detected this variant in one sporadic case of benign familial neonatal seizures together with a variant in KCNQ2. Electrophysiological studies in transiently transfected CHO cells showed no significant functional consequence of this variant[^92^](#_ENREF_92)  This variant was also reported in three unrelated individuals with childhood autism and no history of convulsions; here, the authors reported a significant reduction of potassium current amplitude when the mutant was co-expressed with KV7.5 in Xenopus oocytes[^93^](#_ENREF_93)  Another study reported this variant in a patient with Rolandic epilepsy, developmental delay, ADHD, autistic behavior (only 3 genes were sequenced, this variant was found only in affected patient)[^94^](#_ENREF_94) | VOUS |
| 73450  LOVD  177082 | M | KRT85 | HO, parents HET | chr12:52760957  NM_002283.3:c.233G>A  p.(Arg78His) | Ectodermal dysplasia 4, hair/nail type (AR) (MIM #602032) | Reported in HGMD (DM?) (CM061087)  dbSNP: rs61630004  ExAC: 4508/119964/95  ALAMUT predictions: moderately deleterious  Our patient has thick eyebrows and lashes | Likely benign |
| 73450  LOVD 177082 | M | MIB1 | DN | chr18:19437118_19437119  NM_020774.3:c.2693_2694del  p.(Val898Alafs*7) | Left ventricular noncompaction 7 (AD) (MIM #615092) | Not reported in HGMD  Not in dbSNP or ExAC  2 MIB1 variants were reported in familial cases of left ventricular noncompaction cardiomyopathy (1 missense, 1 stop)[^95^](#_ENREF_95)  One stop variant was reported in a patient with thoracic aortic aneurysm and dissection[^96^](#_ENREF_96)  De Ligt et all, 2012 reported one missense variant in an ID patient (severe developmental delay, behavior problems, self-mutilation, normal cerebral CT, couldn’t speak at adult age, severe ID, normal growth parameters, abnormal breathing pattern, deep set eyes, short philtrum)[^97^](#_ENREF_97)  One FS variant (paternally inherited, rather healthy brother) was reported in 2 siblings with ASD[^98^](#_ENREF_98) | Pathogenic for heart disorder |
| 73450  LOVD 177082 | M | MPO | CH,  PAT  MAT | chr17:56355397  NM_000250.1:c.995C>T  p.(Ala332Val)  chr17:56356502  NM_000250.1:c.752T>C  p.(Met251Thr) | Myeloperoxidase deficiency (AR) (MIM #254600) | Reported in HGMD (DM) (CM041040)  dbSNP: rs28730837  ExAC: 1609/121328/27  ALAMUT predictions: moderately deleterious  Reported in 2 patients in MPObase variation browser: one with partial MPO deficiency and one with complete MPO deficiency.  Reported in HGMD (DM) (CM972840)  dbSNP: rs56378716  ExAC: 1180/97826/9  ALAMUT predictions: deleterious  Reported in 5 patients with partial or complete MPO deficiency (MPObase variation browser) | Pathogenic for MPO deficiency |
| 68944  LOVD 181170 | M | NDUFA1 | XL, MAT | chrX:119005968  NM_004541.3:c.94G>C  p.(Gly32Arg) | Mitochondrial complex I deficiency (XLD) (MIM #252010) | Not in the healthy brother of our patient  Reported in HGMD (DFP) (CM091572)  dbSNP: rs1801316  ExAC: 545/87696/1/206  ALAMUT predictions: benign  Potluri et al., 2009[^99^](#_ENREF_99) reported this variant in a family with 3 affected males: progressive mitochondrial complex I-specific neurodegenerative disease; Piton et al., 2013 reported this variant in 18 unaffected males and conclude that this variant is likely not affecting function or at least not fully penetrant[^100^](#_ENREF_100) | Likely benign |
| 72440  LOVD 181150 | M | NPC1 | PAT | chr18:21141461  NM_000271.4:c.494C>A  p.(Ala165Asp) | Niemann-Pick disease, type C1 and D (AR) (MIM #257220) | c.494C>T; p.(Ala165Val) reported in HGMD  ALAMUT predictions: moderately deleterious  Not in dbSNP or ExAC | VOUS |
| 71412  LOVD 181179 | F | OTOF | DN | chr2:26717853  NM_194248.2:c.854dup  p.(Tyr285*) | Auditory neuropathy, autosomal recessive, 1 (AR) and  Deafness, autosomal recessive 9 (AR) (MIM #601071) | Not reported in HGMD  Not in dbSNP or ExAC | Carrier for AR deafness |
| 69314  LOVD 177065 | F | PDE11A | CH,  MAT  PAT | chr2:178528608  NM_016953.3:c.2632A>G  p.(Met878Val)  chr2:178565891  NM_016953.3:c.2202del  p.(Glu734Aspfs*9) | Pigmented nodular adrenocortical disease, primary, 2 (AD) (MIM #610475) | Reported in HGMD (DM?) (CM094023)  dbSNP: rs74357545  ExAC: 788/121148/4  ALAMUT predictions: moderately deleterious  Not reported in HGMD  Not in dbSNP or ExAC  2 LoF variants reported in adrenocortical hyperplasia; truncating variants predispose to a variety of endocrine tumors, including adrenal and testicular tumors[^101-104^](#_ENREF_101) | Likely benign  Pathogenic for predisposition to endocrine tumors and adrenal Cushing syndrome |
| 76366  LOVD 177066 | M | PIF1 | HO, parents HET | chr15:65116390  NM_025049.3:c.145G>T  p.(Glu49*) | No disease reported in OMIM | Reported in HGMD (DM) (CM1313558)  ExAC: 59/3762/0  dbSNP: rs75683534  This variant has been reported **heterozygous** in one patient with multiple sessile serrated adenoma[^105^](#_ENREF_105) | VOUS |
| 48264  LOVD 181183 | F | PKD1 | MAT | chr16:2158672  NM_001009944.2:c.6496C>T  p.(Arg2166Cys) | Polycystic kidney disease, adult type I (AD) (MIM #173900) | Reported in HGMD (DM) (CM074428)  dbSNP: rs146096401  ExAC:121/23946/2  ALAMUT predictions: deleterious  Reported twice in Polycystic kidney disease 1, each time in combination with other PKD1 variants potentially affecting function in the same patient[^106^](#_ENREF_106)^,^[^107^](#_ENREF_107) | VOUS |
| 68047  LOVD 181184 | M | PLXNA3 | XL, MAT | chrX:153690631  NM_017514.4:c.1298G>A  p.(Arg433His) | No disease reported in OMIM | Reported in HGMD (DM?) (CM140393) for nonsyndromic intellectual disability  dbSNP: rs141197316  ExAC:734/ 80782/3/289  ALAMUT predictions: moderately deleterious  This gene is often mutated in our cohort | Likely benign |
| 71118  LOVD 181185 | M | POF1B | XL, MAT  (mother het) | chrX:84563194  NM_024921.3:c.986G>A  p.(Arg329Gln) | Premature ovarian failure 2B (MIM #300604) | Reported in HGMD (DFP) (CM066591)  dbSNP: rs75398746  ExAC: 31/84789/1/78  ALAMUT predictions: benign  Reported in homozygous state in affected women of a consanguineous family and with functional supporting evidence[^108^](#_ENREF_108) | Carrier of pathogenic variant for Premature ovarian failure 2B |
| 62075  LOVD 181186 | F | PRODH | CH  MAT  PAT | chr22:18905899  NM_016335.4:c.1357C>T  p.(Arg453Cys)  chr22:18901004  NM_016335.4:c.1562A>G  p.(Gln521Arg) | Hyperprolinemia, type I (AR) (MIM #239500)  Schizophrenia, susceptibility to, 4 (AD) (MIM #600850) | Reported in HGMD (DP) (CM057360)  dbSNP: [rs3970559](http://www.ncbi.nlm.nih.gov/SNP/snp_ref.cgi?type=rs&rs=rs3970559)  ExAC: 1473/120628/20  ALAMUT predictions: deleterious  ClinVar: pathogenic, risk factor (RCV000004215.4:Risk factor - Schizophrenia 4; RCV000004214.4: Pathogenic - Proline dehydrogenase deficiency)  Reported in HGMD (DFP) (CM057943) in increased enzyme activity and hyperprolinemia type I  dbSNP: [rs450046](http://www.ncbi.nlm.nih.gov/SNP/snp_ref.cgi?type=rs&rs=rs450046), MAF = 0.0944/473 (1000 Genomes)  ExAC: 60990/66504/27922 (low quality call)  ALAMUT predictions: benign  ClinVar: pathogenic, risk factor (RCV000004223.5: Risk factor - Schizophrenia 4; RCV000004222.5: Pathogenic - Proline dehydrogenase deficiency)  Bender et al., 2005 analyzed the functional consequence of these variants and conclude that Arg453Cys result in severe (>70%) reduction in proline oxidase activity, while Gln521Arg increases it[^109^](#_ENREF_109)  Thus the two variants may compensate each other; accordingly, the proline level of the patient was normal | Carrier of two pathogenic variants for Proline dehydrogenase deficiency, likely compensating each other |
| 71592  LOVD 181187 | M | RAF1 | MAT | chr3:12627296  NM_002880.3:c.1420A>G  p.(Ile474Val) | Noonan syndrome 5 (MIM #611553) | Not reported in HGMD  dbSNP: rs759107333  ExAC: 1/121284/0  ALAMUT predictions: deleterious  CADD score: 22.4  Inherited from the mother who has depression and has mildly low set ears;  variant located in the activated segment of the protein kinase domain with nearby variants causing Noonan syndrome[^110^](#_ENREF_110)  Val474 lacks the Cδ-methyl group and consequently shows no interactions with Val537, which is expected to destabilize the enzyme, see also supplemental table S6 | Likely pathogenic for Noonan syndrome 5 |
| 70757  LOVD 176997 | F | RP1L1 | PAT | chr8:10465490  NM_178857.5:c.6118G>T  p.(Glu2040*) | Occult macular dystrophy (AD) (MIM #613587) | Not reported in HGMD  dbSNP: rs201774530  ExAC: 9/120764/0  Only missense variants reported in occult Macular dystrophy (HGMD)  One LoF variant reported in Retinitis pigmentosa^[111](#_ENREF_111)^  This variant is located at the end of the last exon | VOUS |
| 68944  LOVD 181170 | M | RYR2 | PAT | chr1:237711862  NM_001035.2:c.3038G>A  p.(Arg1013Gln) | Arrhythmogenic right ventricular dysplasia 2 (AD) (MIM #600996) and  Ventricular tachycardia, catecholaminergic polymorphic, 1 (AD) (MIM #604772) | Reported in HGMD (DM) (CM097930)  dbSNP: rs149514924  ExAC: 55/120324/0  ALAMUT predictions: deleterious  Reported in one patient with Catecholaminergic polymorphic ventricular tachycardia by Medeiros-Domingo et al., 2009[^112^](#_ENREF_112); leaky Ca^2+^ release channel/ryanodine receptor 2 causes seizures and sudden cardiac death in mice[^113^](#_ENREF_113)  Classified as VOUS in several studies[^114-116^](#_ENREF_114) | VOUS |
| 73134  LOVD 181190 | M | SCN1A | PAT | chr2:166901590  NM_001165963.1:c.1625G>A  p.(Arg542Gln) | Epilepsy, generalized, with febrile seizures plus, type 2 (AD) (MIM 604403) and  Epileptic encephalopathy, early infantile, 6 (AD) (MIM # 607208) and  Febrile seizures, familial, 3A (AD) (MIM 604403) and  Migraine, familial hemiplegic, 3 (AD) (MIM 609634) | Reported in HGMD (DM?) (CM034567)  dbSNP: rs121918817  ExAC: 186/121374/0  ALAMUT predictions: deleterious  Inherited from the father, the father and grandfather had each one seizure in adolescence  This variant is reported with conflicting support for functional effects  Reported in familial autism[^117^](#_ENREF_117), Juvenile myoclonic epilepsy[^118^](#_ENREF_118), Generalized epilepsy with febrile seizures[^119^](#_ENREF_119), Intractable epilepsy[^120^](#_ENREF_120) and epileptic encephalopathy[^121^](#_ENREF_121) and reported likely not affecting function based on higher prevalence in controls than in patients on previous studies by Lal et al., 2016[^122^](#_ENREF_122)  No 3D protein structure available for modeling | VOUS |
| 69986  LOVD 176985 | F | SDHAF2 | DN | chr11:61205299  NM_017841.2:c.239T>C  p.(Leu80Ser) | Paragangliomas 2 (AD) (MIM #601650) | Not in HGMD, but two missense variants (both leading to p.(G78R)) and 3 LoF variant reported in HGMD for paraganglioma  dbSNP: rs376560419  ExAC: 3/119826/0  ALAMUT predictions: deleterious | VOUS |
| 71118  LOVD 181185 | M | TEX13B | XL, MAT | chrX:107224976  NM_031273.2:c.382C>T  p.(Gln128*) | No disease reported in OMIM | Not reported in HGMD, only one splice site variant reported in ASD (DM?)[^123^](#_ENREF_123)  dbSNP: rs41304466  ExAC: 504/86063/1/178 | Likely benign |
| 75605  LOVD 181193 | M | TGFBI | DN | chr5:135382090  NM_000358.2:c.365C>T  p.(Thr122Met) | Corneal dystrophy, Avellino type (AD) (MIM #607541) and  Corneal dystrophy, epithelial basement membrane (AD) (MIM #121820) and  Corneal dystrophy, Groenouw type I (AD) (MIM #121900) and  Corneal dystrophy, lattice type I (AD) (MIM #122200) and  Corneal dystrophy, lattice type IIIA (AD) (MIM #608471) and  Corneal dystrophy, Reis-Bucklers type (MIM #608470) and  Corneal dystrophy, Thiel-Behnke type (AD) (MIM #602082) | Not reported in HGMD, but variants affecting the neighboring amino acids (p.(D123H), p.(R124C/H/L/S)) are reported in Corneal dystrophy (HGMD)  dbSNP: rs780759366  ExAC: 10/120390/0  ALAMUT predictions: deleterious  This gene encodes an RGD-containing protein that binds to type I, II and IV collagens. The RGD motif is found in many extracellular matrix proteins modulating cell adhesion and serves as a ligand recognition sequence for several integrins. This protein plays a role in cell-collagen interactions and may be involved in endochondrial bone formation in cartilage. (NCBI Gene ID 7045) | VOUS |
| 43990  LOVD 181194 | M | TNFRSF1A | PAT | chr12:6442643  NM_001065.3:c.362G>A  p.(Arg121Gln) | Periodic fever, familial (AD) (MIM #142680) | Reported in HGMD (DM) (CM012483)  dbSNP: rs4149584  ExAC: 1701/120556/14  ALAMUT predictions: benign  [Maillard-Lefebvre](http://www.sciencedirect.com/science/article/pii/S0755498212005106) et al., 2013: R92Q (Arg121Gln) is one of 60 different variants currently described. R92Q is associated with an atypical or incomplete phenotype: later onset, shorter duration of attacks and less frequent abdominal pain. This variant has a low penetrance thus accounting for the sporadic cases. Recently, R92Q raises a lot of discussion about its direct functional effect as its frequency in the general population range from 1 to 8%. Others authors consider than R92Q variant can be regarded as a low-penetrance variant with a mild and broad contribution to auto-inflammatory disease, most likely depending on other modifying genes or environmental factors[^124^](#_ENREF_124) | Likely benign |
| 76366  LOVD 177066 | M | TRAPPC2 | XL, MAT | chrX:13752217_13752223  NM_001128835.2:c.29_35del  p.(Gly10Aspfs*5) | Spondyloepiphyseal dysplasia tarda (XLR) (MIM #313400) | Not reported in HGMD and HGMD isoform (NM_001011658.3) not affected. This variant only affects 2 of 3 RefSeq isoforms and only one of them in a coding exon  Not in dbSNP or ExAC  More than 25 LoF variants reported in HGMD (DM) in Spondyloepiphyseal dysplasia tarda | VOUS |
| 68944  LOVD 181170 | M | TSC2 | MAT | chr16:2138570  NM_000548.3:c.5383C>T  p.(Arg1795Cys) | Lymphangioleiomyomatosis, somatic (MIM #606690) and  Tuberous sclerosis-2 (AD) (MIM #613254) | Reported in HGMD (DM) (CM014752)  dbSNP: rs45517423  ExAC: 143/117156/0  ALAMUT predictions: deleterious  Classified as VOUS or benign in several studies[^114-116^](#_ENREF_114)^,^[^125^](#_ENREF_125)  Inherited from the mother who has never been evaluated with imaging of the brain or kidney | Likely benign |
| 43092  LOVD 177013 | M | TTN | PAT | chr2:179631201  NM_133378.4:c.9610C>T  p.(Arg3204*) | Cardiomyopathy, dilated, 1G (MIM #604145) and  Cardiomyopathy, familial hypertrophic, 9 (AD) (MIM #613765) and  Muscular dystrophy, limb-girdle, type 2J (AR) (MIM #608807) and  Myopathy, proximal, with early respiratory muscle involvement (MIM#603689) and  Salih myopathy (AR) (MIM #611705) and  Tibial muscular dystrophy, tardive (AD) (MIM #600334) | Not reported in HGMD  dbSNP: rs757836789  Not in ExAC  ClinVar: Uncertain significance (RCV000338005.1)  Dominant LoF variants in this region (I-band of the protein) were described in patients with dilated cardiomyopathy.[^126^](#_ENREF_126)^,^[^127^](#_ENREF_127) A compound heterozygous variant (one splice-site variant in I-band of the protein, and a missense variant in M-band of the protein) was identified in a patient with core myopathy with heart disease.[^128^](#_ENREF_128)  A functional study using iPS cells derived from patients with variants in I- or A-band of TTN demonstrates that LoF variants in this gene cause dilated cardiomyopathy by disrupting critical linkages between sarcomerogenesis and adaptive remodeling, based on different functional effects. They showed that the deleterious functional effect of I-band FS variants is the result of alternative splicing with exclusion of I-band exons while the A-band domain is still present in the mutant TTN protein. TTN LoF variants in the A-band produce longer and stable mutant proteins with a dominant negative effect on sarcomere biology.[^129^](#_ENREF_129)  Taking into account that many LoF variants in I-band of TTN protein have been identified in general population without dilated cardiomyopathy[^130^](#_ENREF_130), the unknown functional impact of our mutant protein on sarcomere biology, and in absence of family history related to this disorder, we classified this variant as VOUS. | VOUS |
| 47651  LOVD 177015 | M | USP26 | XL, MAT | chrX:132160273  NM_031907.1:c.1976C>T  p.(Thr659Met) | No disease reported in OMIM | Reported in HGMD (DM) (CM056717)  dbSNP: rs145695346  ExAC: 125/87501/0/47  ALAMUT predictions: benign  Reported in azoospermia in one association study with only 17 controls[^131^](#_ENREF_131) | Likely benign |
| 73704 / 46917  LOVD 181199 | F | WNT10A | CH,  MAT  PAT | chr2:219754822  NM_025216.2:c.493G>A  p.(Gly165Arg)  chr2:219754840  NM_025216.2:c.511C>T  p.(Arg171Cys) | Odontoonychodermal dysplasia (AR) (MIM #257980) and  Schopf-Schulz-Passarge syndrome (AR) (MIM #224750) and  Tooth agenesis, selective, 4 (AD, AR) (MIM #150400) | Reported in HGMD (DM?) (CM138364)  dbSNP: rs77583146  ExAC: 888/121056/4  ALAMUT predictions: deleterious  In one study found in compound heterozygous constellation with p.(Asn363His) in an unaffected individual[^132^](#_ENREF_132)  Reported in HGMD (DM) (CM1313214)  dbSNP: rs116998555  ExAC: 246/120922/1  ALAMUT predictions: deleterious | Likely benign  VOUS |

ALAMUT predictions include SIFT [^68^](#_ENREF_68), Mutationtaster [^69^](#_ENREF_69) and PolyPhen-2 [^70^](#_ENREF_70): deleterious (at least 2 of 3), moderately deleterious (1 of 3), tolerated (all 3 predictions benign)

HGMD categorization: DM Disease causing mutation; DM? Likely disease causing mutation; DP Disease-associated polymorphism; DFP Disease associated polymorphism with additional supporting functional evidence

Table S8. Summary of relevant findings per patient

Color code: Pink = (likely) pathogenic, yellow = carrier of a pathogenic recessive allele, blue = incidental finding, white = candidate gene

| **Patient ID^1^**  **Decipher or LOVD ID** | **Sex** | **Gene / region** | **Inheritance** | **Type of variant** | **Genome position (hg19) and HGVS variant nomenclature** | **Predicted protein effect** | **Known disorder**  **(MIM number)** | **Relevance of finding** |
| --- | --- | --- | --- | --- | --- | --- | --- | --- |
| 32546  Decipher 370080 | M | **NPHP1** | AR, NA | Heterozygous, CNV (862Kb) | chr2:g.(110498142_110504318)_(111365996_111369233)del | Loss (NPHP1) | Recessive allele for nephronophthisis 1 and Joubert syndrome 4 (607100) | Carrier of recessive allele |
| 33386  LOVD 177064 | F | **GTF3C3** | CH | Heterozygous missense (PAT) | chr2:197631408  NM_012086.4:c.2419C>T | p.(Arg807Cys) | - | Recurrent candidate gene |
|  |  |  |  | Heterozygous missense (MAT) | chr2:197656093  NM_012086.4:c.503C>T | p.(Ala168Val) |  |  |
| 34124  LOVD 177009 | M | **SCN1A** | AD, DN | Heterozygous missense | chr2:166848437  NM_001165963.1:c.5348C>T | p.(Ala1783Val) | Dravet syndrome (607208) | Pathogenic |
| 42680  177012 | M | **SCN2A** | AD, DN | Heterozygous missense | chr2:166245724  NM_021007.2:c.5408A>G | p.(Glu1803Gly) | Epileptic encephalopathy, early infantile, 11 (613721) | Likely pathogenic |
| 43092  LOVD 177013 | M | **SCN8A** | AD, DN | Heterozygous missense | chr12:52200885  NM_014191.3:c.5615G>A | p.(Arg1872Gln) | Epileptic encephalopathy, early infantile, 13 (614558) | Pathogenic |
|  |  | **CHST6** | AR, NA | Heterozygous missense | chr16:75513536  NM_021615.4:c.191C>T | p.(Pro64Leu) | Macular corneal dystrophy (AR) (217800) | Carrier of likely pathogenic variant for AR Macular corneal dystrophy |
| 47651  LOVD 177015 | M | **SPATA5** | AR, CH | CNV (51 Kb) (MAT) | chr4:g.(123951799_123952079)_(124003383_ 124003384)del | Loss | Epilepsy, hearing loss, and mental retardation  syndrome (616577) | Likely pathogenic |
|  |  |  |  | In-frame deletion (PAT) | chr4:123855729  NM_145207.2: c.989_991del | p.(Thr330del) |  |  |
| 47970  LOVD 177010 | M | **SCN1A** | AD, DN | Heterozygous frameshift | chr2:166850754  NM_001165963.1:c.4754del | p.(Thr1585Metfs*6) | Dravet syndrome (607208) | Pathogenic |
| 49635  Decipher 370081 | M | **UBE3A** | AD, DN, maternal allele | Heterozygous, CNV (134 Kb) | chr15:g.(25583244_25583408)_(25717757_ 25717851)del | Loss (UBE3A) | Angelman Syndrome (105830) | Pathogenic |
| 50126  Decipher 370070 | M | **MBD5** | AD, DN | Heterozygous CNV (197 Kb) | chr2:g.(148757084_148762374)_(148959158_ 148960882)del | Loss (MBD5,ORC4) | Mental retardation, autosomal dominant 1 (156200) | Pathogenic |
| 52236  LOVD 181099 | F | **STXBP1** | AD, DN | Heterozygous splice site | chr9:130422393  NG_016623.1(NM_003165.3):c.325+6T>C | p.(?) | Epileptic encephalopathy, early infantile, 4 (612164) | Pathogenic |
| 56302  LOVD  181169 | M | **GALNTL5** | AD (?), inherited from mother | Heterozygous frameshift | chr7: 151664484  NM_145292.3:c.153dup | p.(Val52Serfs*23) | One frameshift variant reported in Asthenozoospermia with supporting functional studies in a mouse model[^86^](#_ENREF_86) | Incidental finding: Pathogenic for Asthenozoospermia (?) |
| 59248  LOVD 181166 | F | **DPM1** | AR, inherited from father | Heterozygous missense | chr20:49565187  NM_003859.1:c.274C>G | p.(Arg92Gly) | Congenital disorder of glycosylation, type Ie (608799) | Carrier for AR congenital disorder of glycosylation, type Ie |
| 62075  LOVD 181186 | F | **PRODH** | AR, CH | Heterozygous missense (MAT) | chr22:18905899  NM_016335.4:c.1357C>T | p.(Arg453Cys) | Hyperprolinemia, type I (239500) | Carrier of two pathogenic variants for AR Proline dehydrogenase deficiency, likely compensating each other |
|  |  |  |  | Heterozygous missense (PAT) | chr22:18901004  NM_016335.4:c.1562A>G | p.(Gln521Arg) |  |  |
| 68944  LOVD 181170 | M | **GJB2** | AR, CH | Heterozygous frameshift (PAT) | chr13:20763686  NM_004004.5:c.35del | p.(Gly12Valfs*2) | AR deafness (HGMD accession numbers CD972240 and CM970679) | Incidental finding: Carrier for AR non-syndromic deafness |
|  |  |  |  | Heterozygous missense (MAT) | chr13:20763620  NM_004004.5:c.101T>C | p.(Met34Thr) |  | Incidental finding:  Likely pathogenic for AR late onset mild hearing impairment |
| 69314  LOVD 177065 | F | **PIK3AP1** | AD, DN | Heterozygous missense | chr10:98411052  NM_152309.2:c.941T>C | p.(Leu314Pro) | 2 *de novo* missense variants reported in 2 independent patients with infantile spasms[^57^](#_ENREF_57) | Recurrent candidate gene |
|  |  | **PDE11A** | AD, inherited from father | Heterozygous frameshift | chr2:178565891  NM_016953.3:c.2202del | p.(Glu734Aspfs*9) | Pigmented nodular adrenocortical disease, primary, 2 (610475) | Incidental finding: pathogenic for predisposition to endocrine tumors and adrenal Cushing syndrome |
| 69937  LOVD 177008 | M | **PRUNE1** | AR, HO | Homozygous missense | chr1:150991126  NM_021222.2:c.316G>A | p.(Asp106Asn)  see also supplemental table S6 | Recently described gene in recessive brain malformation. Five patients described with microcephaly, cortical atrophy, thin or hypoplastic corpus callosum and prominent cerebellar atrophy, 2 patients with seizures.[^37^](#_ENREF_37)  15 further patients described with similar MRI anomalies, profound intellectual disability, seizures in 6 patients, progressive course and early death in at least 3 patients.[^133^](#_ENREF_133) Further patients described.[^134^](#_ENREF_134)^,^[^135^](#_ENREF_135) | Likely pathogenic |
| 69986  LOVD 176985 | F | **ACO2** | AR, CH | Heterozygous missense (PAT) | chr22:41922363  NM_001098.2:c.1859G>A | p.(Gly620Asp) | Infantile cerebellar-retinal degeneration (614559) | Likely pathogenic |
|  |  |  |  | Heterozygous missense (MAT) | Chr22:41923386  NM_001098.2:c.2048G>T | p.(Gly683Val) |  |  |
| 70757  LOVD 176997 | F | **AP4S1** | AR, HO | Homozygous splice site | chr14:31535539 NG_031913.1(NM_007077.4): c.138+3_138+6del | p.(?) | Spastic paraplegia 52, autosomal recessive (614067) | Pathogenic |
| 71118  LOVD 181185 | M | **POF1B** | XL  mother het | Hemizygous missense | chrX:84563194  NM_024921.3:c.986G>A | p.(Arg329Gln) | Premature ovarian failure 2B (300604) | Carrier of pathogenic variant for Premature ovarian failure 2B |
| 71412  Decipher 370084  LOVD 181179 | F | **15q11.2**  **q13.11 (UBE3A)** | AD, DN, maternal allele | Heterozygous, CNV (4.9 Mb) | chr15:g.(23620154_23620191)_(28545355_ 28545445)del | Loss (19 OMIM genes out of a total of 105) | Angelman Syndrome (105830) | Pathogenic |
|  |  | **OTOF** | DN | Heterozygous stop | chr2:26717853  NM_194248.2:c.854dup | p.(Tyr285*) | Auditory neuropathy, autosomal recessive, 1 and  Deafness, autosomal recessive 9 (601071) | Carrier for AR deafness |
| 71592  Decipher 370076  LOVD 181187 | M | **22q11.22 -q11.23** | AD, DN | Heterozygous CNV (2 Mb) | chr22:g.(22953405_22953514)_(25026857_25027086)dup | Gain (25 OMIM genes, total of 45) | 22q11.22q11.23 recurrent microduplication | Pathogenic |
|  |  | **RAF1** | AD, inherited from mother | Heterozygous missense | chr3:12627296  NM_002880.3:c.1420A>G | p.(Ile474Val) | Noonan syndrome 5 (611553) | Incidental finding: likely pathogenic for Noonan syndrome 5 |
| 71693  LOVD 177006 | M | **POLG** | AR, CH  Likewise affected sister also CH | Heterozygous missense  (not in mother, father not available) | chr15:89865023 NM_002693.2:c.2542G>A | p.(Gly848Ser) | *POLG*-related disorder[^34^](#_ENREF_34) | Pathogenic |
|  |  |  |  | Heterozygous missense (MAT) | chr15:89873343  NM_002693.2:c.824G>A | p.(Arg275Gln) |  |  |
| 72128  Decipher 370072 | F | **CDKL5** | XL-D, DN | Heterozygous mosaic CNV (25 Kb) | chrX:g.(18592712_18592741)_(18617503_ 18617862)del | Loss (CDKL5) | Epileptic encephalopathy, early infantile, 2 (300672) | Pathogenic |
| 72356  Decipher 370071 | F | **1p36.23-pter** | AD, DN | CNV (7.7 Mb) | chr1:g.(0_849466)_(7666975_7683885)del | Loss (62 OMIM genes, total of 118) | 1p36 deletion syndrome (607872) | Pathogenic |
| 72404  LOVD 177004 | M | **CDKL5** | XL-D, DN | Hemizygous splice site | chrX:18593609  NG_008475.1(NM_003159.2):c.282+3_282+6del | p.(?) | Epileptic encephalopathy, early infantile, 2 (300672) | Pathogenic |
| 72440  LOVD 181150 | M | **ABCC2** | AR, inherited from mother | Heterozygous splice site | chr10:101591889  NG_011798.1(NM_000392.43):c.3258+1G>A | p.(?) | Dubin-Johnson syndrome (237500) | Carrier of pathogenic variant for AR Dubin-Johnson syndrome |
| 72555 / 72719  LOVD 177014 | both M | **SMS** | XL-R, in mother DN | Hemizygous missense | chrX:21995237  NM_004595.4:c.388C>T | p.(Arg130Cys) | Snyder-Robinson syndrome (182290); our patients are described in great detail in Abela et al., 2016[^43^](#_ENREF_43) | Pathogenic |
|  |  | **FIG4** | AD/AR, inherited from father | Heterozygous frameshift | chr6:110098182  NM_014845.5:c.1808_1811dup | p.(Pro605Glufs*45) | Amyotrophic lateral sclerosis 11 (AD) (612577)  Charcot-Marie-Tooth disease, type 4J (AR) (611228) | Carrier of a likely pathogenic variant for AR Charcot-Marie-Tooth disease type 4J |
| 72892  LOVD 177002 | M | **ARX** | XL-R, mother het | Hemizygous missense | chrX:25031055  NM_139058.2:c.1057C>T | p.(Pro353Ser)  see also supplemental table S6 | Epileptic encephalopathy, early infantile, 1 (308350) | Likely pathogenic |
| 72943  LOVD 177024 | M | **SZT2** | AR | Heterozygous frameshift | chr1:43885569  NM_015284.3:c.1045del | p.(Ser349Profs*9) | Epileptic encephalopathy, early infantile, 18  (615476) | Likely pathogenic |
|  |  |  |  | DN, heterozygous missense | chr1:43888272  NM_015284.3:c.1891G>A | p.(Glu631Lys) |  |  |
| 73068  LOVD 177016 | M | **SPATA5** | AR, CH | Heterozygous missense, DN, paternal allele | chr4:124177219  NM_145207.2:c.2389C>G | p.(Pro797Ala) | Epilepsy, hearing loss, and mental retardation  syndrome (616577) | Likely pathogenic |
|  |  |  |  | Heterozygous loss of the primary start codon (MAT) | chr4:123844298  NM_145207.2:c.1A>C | p.(Met1?) |  |  |
| 73214  LOVD 176996 | F | **KCNQ2** | AD, DN | Heterozygous missense | chr20:62073835  NM_172107.2:c.740C>T | p.(Ser247Leu) | Epileptic encephalopathy, early infantile, 7 (613720) | Pathogenic |
| 73311 /  76351  LOVD 177003 | both M | **BRAT1** | AR, CH | Heterozygous frameshift (PAT) | chr7:2578041  NM_152743.3:c.2125_2128del | p.(Phe709Thrfs*17) | Rigidity and multifocal seizure syndrome, lethal neonatal (614498) | Pathogenic |
|  |  |  |  | Heterozygous frameshift (MAT) | chr7:2583389  NM_152743.3:c.638dup | p.(Val214Glyfs*189) |  |  |
| 73324  LOVD 177005 | F | **GABRB2** | AD, DN | Heterozygous missense | chr5:160761872  NM_021911.2:c.719G>C | p.(Arg240Thr)  see also supplemental table S6 | Novel gene, reported in one patient with intellectual disability & epilepsy[^29^](#_ENREF_29), in two patients with epilepsy[^30^](#_ENREF_30) and eleven patients with mostly ID and refractory seizures[^31^](#_ENREF_31) | Likely pathogenic |
| 73450  LOVD 177082  Decipher 370095 | M | **WRAP53** | AR, CH | Heterozygous missense (MAT) | chr17:7605740  NM_018081.2:c.1034A>G | p.(Tyr345Cys) | Dyskeratosis congenita (DC), autosomal recessive 3 (613988) with extended clinical phenotype including microcephaly, ataxia, developmental delay, intrauterine growth retardation as observed in other DC subtypes and absence seizures as novel finding for DC | Candidate gene |
|  |  |  |  | Heterozygous missense (PAT) | chr17:7606345  NM_018081.2:c.1303G>A | p.(Gly435Arg) |  |  |
|  |  | **ACTA1** | AR, inherited from father | Heterozygous, CNV (32 Kb) | chr1:g.(229545391_229545536)_(229577325_229577431)del | Loss (ACTA1) | Nemaline Myopathy 3 (161800) | Carrier of recessive allele |
|  |  | **MIB1** | AD, DN | Heterozygous frameshift | chr18:19437118_19437119  NM_020774.3:c.2693_2694del | p.(Val898Alafs*7) | Left ventricular noncompaction 7 (615092) | Incidental finding: pathogenic for heart disorder |
|  |  | **MPO** | AR, CH | Heterozygous missense (PAT) | chr17:56355397  NM_000250.1:c.995C>T | p.(Ala332Val) | Myeloperoxidase deficiency (254600) | Incidental finding: pathogenic for MPO deficiency |
|  |  |  |  | Heterozygous missense (MAT) | chr17:56356502  NM_000250.1:c.752T>C | p.(Met251Thr) |  |  |
| 73805  LOVD 177023 | M | **STXBP1** | AD, DN | Heterozygous missense | chr9:130438941  NM_003165.3:c.1268T>C | p.(Leu423Pro) | Epileptic encephalopathy, early infantile, 4 (612164) | Likely pathogenic |
| 75143  LOVD 177011 | F | **SCN1A** | AD, DN | Heterozygous frameshift | chr2:166904165  NM_001165963.1:c.1142del | p.(Gln381Argfs*10) | Dravet syndrome (607208) | Pathogenic |
| 75605  Decipher 370077 | M | **FARS2** | AR, inherited from mother | Heterozygous, CNV (282 Kb) | chr6:g.(5172637_5172637)_(5454482_5455052)del | Loss (FARS2) | Combined oxidative phosphorylation deficiency 14 (614946) | Carrier of recessive allele |
| 76366  LOVD 177066 |  | **UFC1** | HO | Homozygous missense  (parents heterozygous) | chr1:161123855  NM_016406.3:c.68G>A | p.(Arg23Gln) | - | Recurrent candidate gene |

Note: ^a^ two ID numbers indicate affected siblings with the same phenotype

**Figure S1. Interphase FISH with a red STS probe and a green centromeric X probe indicating triplication of the STS gene region in the female patient 48264.** Two representative nuclei stained blue by DAPI illustrating presence of one single red signal in the proximity of one green signal, and three additional red signals closely lying next to each other in the proximity of the second green signal indicating a heterozygous triplication of the STS locus in Xp22.3 on one X-chromosome, while the other chromosome is normal). Probes: LSI STS (Xp22.3), Spectrum orange / CEP X, Spectrum green (Vysis).


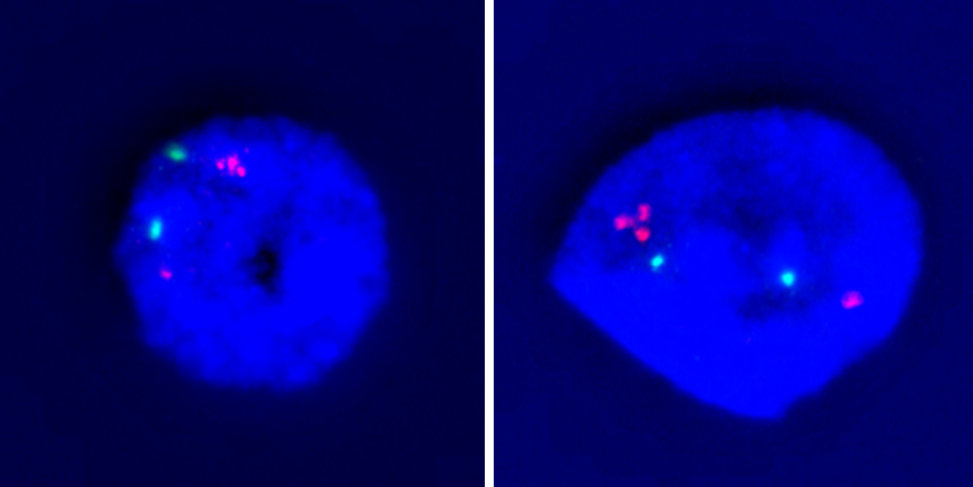


**Web Resources**

CADD Score Combined Annotation Dependent Depletion Score <http://cadd.gs.washington.edu/> [^136^](#_ENREF_136) retrieved with NextGENe Software (SoftGenetics, State College, PA)

ClinVar <http://www.ncbi.nlm.nih.gov/clinvar> [^137^](#_ENREF_137)

COSMIC Catalogue of somatic mutations in cancer <http://cancer.sanger.ac.uk/> [^138^](#_ENREF_138)

dbSNP <https://www.ncbi.nlm.nih.gov/snp/> [^139^](#_ENREF_139)

DECIPHER Database of Chromosome Imbalance and Phenotype in Humans using Ensemble Resources <https://decipher.sanger.ac.uk/> [^140^](#_ENREF_140)

DGV Database of Genomic Variants <http://dgv.tcag.ca/> [^141^](#_ENREF_141)

ExAC Exome Aggregation Consortium <http://exac.broadinstitute.org/> [^142^](#_ENREF_142)

GO Gene Ontology <http://www.geneontology.org/> [^143^](#_ENREF_143)

GTEx Portal Genotype-Tissue Expression Project <http://www.gtexportal.org/>

HGMD Human Gene Mutation Database <http://www.hgmd.org> [^144^](#_ENREF_144)

Human Metabolome database <http://www.hmdb.ca/> [^145^](#_ENREF_145)

ISCA International Standards for Cytogenomic Arrays Consortium <https://www.iscaconsortium.org/>

KEGG Kyoto Encyclopedia of Genes and Genomes <http://www.kegg.jp/kegg/> [^146^](#_ENREF_146)

LOVD Leiden Open Variation Database <http://www.lovd.nl/3.0/home> [^147^](#_ENREF_147)

MetaCyc database <http://www.metacyc.org/> [^148^](#_ENREF_148)

Metlin database <https://metlin.scripps.edu/> [^149^](#_ENREF_149)

MPObase Variation registry for Myeloperoxidase deficiency <http://structure.bmc.lu.se/idbase> [**^150^**](#_ENREF_150)

MutationTaster <http://www.mutationtaster.org> [^69^](#_ENREF_69)

OMIM Online Mendelian Inheritance in Man <https://www.omim.org/>

PDB <http://www.rcsb.org/pdb/> [^151^](#_ENREF_151)

PhosphositePlus <http://www.phosphosite.org/> [^152^](#_ENREF_152)

PolyPhen-2 <http://genetics.bwh.harvard.edu/pph2/> [^70^](#_ENREF_70)

PROSITE <http://prosite.expasy.org/> [^153^](#_ENREF_153)

PubMed <http://www.ncbi.nlm.nih.gov/pubmed>

R software environment for statistical computing and graphics <http://www.r-project.org/>

RefSeq Reference sequence database <https://www.ncbi.nlm.nih.gov/refseq/> [^154^](#_ENREF_154)

SFARI Gene <https://gene.sfari.org/> [^155^](#_ENREF_155)

SIFT Sorting Intolerant From Tolerant <http://sift.jcvi.org/> [^68^](#_ENREF_68)

SysID database <http://sysid.cmbi.umcn.nl/> [^156^](#_ENREF_156)

TMHMM Server <http://www.cbs.dtu.dk/services/TMHMM-2.0/> [^157^](#_ENREF_157)

UCSC Genome Browser [http://genome.ucsc.edu/](https://genome.ucsc.edu/index.html) [^158^](#_ENREF_158)

UniProtKB <http://www.uniprot.org/> [^159^](#_ENREF_159)

**Supplemental References**

1. Asadollahi R, Oneda B, Joset P et al. The clinical significance of small copy number variants in neurodevelopmental disorders. *J Med Genet.* 2014; **51:** 677-688.

2. Ashraf T, Collinson MN, Fairhurst J, Wang R, Wilson LC, Foulds N. Two further patients with the 1q24 deletion syndrome expand the phenotype: A possible role for the miR199-214 cluster in the skeletal features of the condition. *Am J Med Genet A.* 2015; **167A:** 3153-3160.

3. Gregor A, Kramer JM, van der Voet M et al. Altered GPM6A/M6 dosage impairs cognition and causes phenotypes responsive to cholesterol in human and Drosophila. *Hum Mutat.* 2014; **35:** 1495-1505.

4. Stenzel N, Fetzer CP, Heumann R, Erdmann KS. PDZ-domain-directed basolateral targeting of the peripheral membrane protein FRMPD2 in epithelial cells. *J Cell Sci.* 2009; **122:** 3374-3384.

5. Karlsson P, Droce A, Moser JM et al. Loss of vps54 function leads to vesicle traffic impairment, protein mis-sorting and embryonic lethality. *Int J Mol Sci.* 2013; **14:** 10908-10925.

6. Martin HC, Kim GE, Pagnamenta AT et al. Clinical whole-genome sequencing in severe early-onset epilepsy reveals new genes and improves molecular diagnosis. *Hum Mol Genet.* 2014; **23:** 3200-3211.

7. Morimura N, Inoue T, Katayama K, Aruga J. Comparative analysis of structure, expression and PSD95-binding capacity of Lrfn, a novel family of neuronal transmembrane proteins. *Gene.* 2006; **380:** 72-83.

8. Fromer M, Pocklington AJ, Kavanagh DH et al. De novo mutations in schizophrenia implicate synaptic networks. *Nature.* 2014; **506:** 179-184.

9. Sanders SJ, Murtha MT, Gupta AR et al. De novo mutations revealed by whole-exome sequencing are strongly associated with autism. *Nature.* 2012; **485:** 237-241.

10. Uddin M, Thiruvahindrapuram B, Walker S et al. A high-resolution copy-number variation resource for clinical and population genetics. *Genet Med.* 2015; **17:** 747-752.

11. Jacobsson JA, Haitina T, Lindblom J, Fredriksson R. Identification of six putative human transporters with structural similarity to the drug transporter SLC22 family. *Genomics.* 2007; **90:** 595-609.

12. Liu P, Erez A, Nagamani SC et al. Copy number gain at Xp22.31 includes complex duplication rearrangements and recurrent triplications. *Hum Mol Genet.* 2011; **20:** 1975-1988.

13. Li F, Shen Y, Kohler U et al. Interstitial microduplication of Xp22.31: Causative of intellectual disability or benign copy number variant? *Eur J Med Genet.* 2010; **53:** 93-99.

14. Moilanen AM, Rysa J, Kaikkonen L et al. WDR12, a Member of Nucleolar PeBoW-Complex, Is Up-Regulated in Failing Hearts and Causes Deterioration of Cardiac Function. *PLoS One.* 2015; **10:** e0124907.

15. Noskova L, Stranecky V, Hartmannova H et al. Mutations in DNAJC5, encoding cysteine-string protein alpha, cause autosomal-dominant adult-onset neuronal ceroid lipofuscinosis. *Am J Hum Genet.* 2011; **89:** 241-252.

16. Ishida N, Kuba T, Aoki K, Miyatake S, Kawakita M, Sanai Y. Identification and characterization of human Golgi nucleotide sugar transporter SLC35D2, a novel member of the SLC35 nucleotide sugar transporter family. *Genomics.* 2005; **85:** 106-116.

17. Jordan VK, Zaveri HP, Scott DA. 1p36 deletion syndrome: an update. *Appl Clin Genet.* 2015; **8:** 189-200.

18. Shimojima K, Imai K, Yamamoto T. A de novo 22q11.22q11.23 interchromosomal tandem duplication in a boy with developmental delay, hyperactivity, and epilepsy. *Am J Med Genet A.* 2010; **152A:** 2820-2826.

19. Hardies K, May P, Djemie T et al. Recessive loss-of-function mutations in AP4S1 cause mild fever-sensitive seizures, developmental delay and spastic paraplegia through loss of AP-4 complex assembly. *Hum Mol Genet.* 2015; **24:** 2218-2227.

20. Kato M, Das S, Petras K et al. Mutations of ARX are associated with striking pleiotropy and consistent genotype-phenotype correlation. *Hum Mutat.* 2004; **23:** 147-159.

21. Stromme P, Mangelsdorf ME, Shaw MA et al. Mutations in the human ortholog of Aristaless cause X-linked mental retardation and epilepsy. *Nat Genet.* 2002; **30:** 441-445.

22. Smith NJ, Lipsett J, Dibbens LM, Heron SE. BRAT1-associated neurodegeneration: Intra-familial phenotypic differences in siblings. *Am J Med Genet A.* 2016; **170:** 3033-3038.

23. Puffenberger EG, Jinks RN, Sougnez C et al. Genetic mapping and exome sequencing identify variants associated with five novel diseases. *PLoS One.* 2012; **7:** e28936.

24. Fernandez-Jaen A, Alvarez S, So EY et al. Mutations in BRAT1 cause autosomal recessive progressive encephalopathy: Report of a Spanish patient. *Eur J Paediatr Neurol.* 2016; **20:** 421-425.

25. Horn D, Weschke B, Knierim E et al. BRAT1 mutations are associated with infantile epileptic encephalopathy, mitochondrial dysfunction, and survival into childhood. *Am J Med Genet A.* 2016; **170:** 2274-2281.

26. Srivastava S, Olson HE, Cohen JS et al. BRAT1 mutations present with a spectrum of clinical severity. *Am J Med Genet A.* 2016; **170:** 2265-2273.

27. van de Pol LA, Wolf NI, van Weissenbruch MM et al. Early-Onset Severe Encephalopathy with Epilepsy: The BRAT1 Gene Should Be Added to the List of Causes. *Neuropediatrics.* 2015; **46:** 392-400.

28. Fehr S, Wilson M, Downs J et al. The CDKL5 disorder is an independent clinical entity associated with early-onset encephalopathy. *Eur J Hum Genet.* 2013; **21:** 266-273.

29. Srivastava S, Cohen J, Pevsner J et al. A novel variant in GABRB2 associated with intellectual disability and epilepsy. *Am J Med Genet A.* 2014; **164A:** 2914-2921.

30. Hernandez CC, Klassen TL, Jackson LG et al. Deleterious Rare Variants Reveal Risk for Loss of GABAA Receptor Function in Patients with Genetic Epilepsy and in the General Population. *PLoS One.* 2016; **11:** e0162883.

31. Hamdan FF, Myers CT, Cossette P et al. High Rate of Recurrent De Novo Mutations in Developmental and Epileptic Encephalopathies. *Am J Hum Genet.* 2017; **101:** 664-685.

32. Hunter J, Maljevic S, Shankar A et al. Subthreshold changes of voltage-dependent activation of the K(V)7.2 channel in neonatal epilepsy. *Neurobiol Dis.* 2006; **24:** 194-201.

33. Dedek K, Fusco L, Teloy N, Steinlein OK. Neonatal convulsions and epileptic encephalopathy in an Italian family with a missense mutation in the fifth transmembrane region of KCNQ2. *Epilepsy Res.* 2003; **54:** 21-27.

34. Cohen BH, Chinnery PF, Copeland WC: POLG-Related Disorders; in: Pagon RA, Adam MP, Ardinger HH *et al.* (eds): *GeneReviews(R)*. Seattle (WA), 1993.

35. Echaniz-Laguna A, Chassagne M, de Seze J et al. POLG1 variations presenting as multiple sclerosis. *Arch Neurol.* 2010; **67:** 1140-1143.

36. Bereau M, Anheim M, Echaniz-Laguna A et al. The wide POLG-related spectrum: An integrated view. *J Neurol Sci.* 2016; **368:** 70-76.

37. Karaca E, Harel T, Pehlivan D et al. Genes that Affect Brain Structure and Function Identified by Rare Variant Analyses of Mendelian Neurologic Disease. *Neuron.* 2015; **88:** 499-513.

38. Marini C, Mei D, Temudo T et al. Idiopathic epilepsies with seizures precipitated by fever and SCN1A abnormalities. *Epilepsia.* 2007; **48:** 1678-1685.

39. Klassen T, Davis C, Goldman A et al. Exome sequencing of ion channel genes reveals complex profiles confounding personal risk assessment in epilepsy. *Cell.* 2011; **145:** 1036-1048.

40. Larsen J, Carvill GL, Gardella E et al. The phenotypic spectrum of SCN8A encephalopathy. *Neurology.* 2015; **84:** 480-489.

41. Horvath GA, Demos M, Shyr C et al. Secondary neurotransmitter deficiencies in epilepsy caused by voltage-gated sodium channelopathies: A potential treatment target? *Mol Genet Metab.* 2016; **117:** 42-48.

42. Wagnon JL, Barker BS, Hounshell JA et al. Pathogenic mechanism of recurrent mutations of SCN8A in epileptic encephalopathy. *Ann Clin Transl Neurol.* 2016; **3:** 114-123.

43. Abela L, Simmons L, Steindl K et al. N(8)-acetylspermidine as a potential plasma biomarker for Snyder-Robinson syndrome identified by clinical metabolomics. *J Inherit Metab Dis.* 2016; **39:** 131-137.

44. Tanaka AJ, Cho MT, Millan F et al. Mutations in SPATA5 Are Associated with Microcephaly, Intellectual Disability, Seizures, and Hearing Loss. *Am J Hum Genet.* 2015; **97:** 457-464.

45. Buchert R, Nesbitt AI, Tawamie H et al. SPATA5 mutations cause a distinct autosomal recessive phenotype of intellectual disability, hypotonia and hearing loss. *Orphanet J Rare Dis.* 2016; **11:** 130.

46. Di Meglio C, Lesca G, Villeneuve N et al. Epileptic patients with de novo STXBP1 mutations: Key clinical features based on 24 cases. *Epilepsia.* 2015; **56:** 1931-1940.

47. Weckhuysen S, Holmgren P, Hendrickx R et al. Reduction of seizure frequency after epilepsy surgery in a patient with STXBP1 encephalopathy and clinical description of six novel mutation carriers. *Epilepsia.* 2013; **54:** e74-80.

48. Stamberger H, Nikanorova M, Willemsen MH et al. STXBP1 encephalopathy: A neurodevelopmental disorder including epilepsy. *Neurology.* 2016; **86:** 954-962.

49. Boutry-Kryza N, Labalme A, Ville D et al. Molecular characterization of a cohort of 73 patients with infantile spasms syndrome. *Eur J Med Genet.* 2015; **58:** 51-58.

50. Mefford HC, Yendle SC, Hsu C et al. Rare copy number variants are an important cause of epileptic encephalopathies. *Ann Neurol.* 2011; **70:** 974-985.

51. Dumay-Odelot H, Marck C, Durrieu-Gaillard S et al. Identification, molecular cloning, and characterization of the sixth subunit of human transcription factor TFIIIC. *J Biol Chem.* 2007; **282:** 17179-17189.

52. Male G, von Appen A, Glatt S et al. Architecture of TFIIIC and its role in RNA polymerase III pre-initiation complex assembly. *Nat Commun.* 2015; **6:** 7387.

53. Borck G, Hog F, Dentici ML et al. BRF1 mutations alter RNA polymerase III-dependent transcription and cause neurodevelopmental anomalies. *Genome Res.* 2015; **25:** 155-166.

54. Jee YH, Sowada N, Markello TC, Rezvani I, Borck G, Baron J. BRF1 mutations in a family with growth failure, markedly delayed bone age, and central nervous system anomalies. *Clin Genet.* 2017; **91:** 739-747.

55. Reuter MS, Tawamie H, Buchert R et al. Diagnostic Yield and Novel Candidate Genes by Exome Sequencing in 152 Consanguineous Families With Neurodevelopmental Disorders. *JAMA Psychiatry.* 2017; **74:** 293-299.

56. Anazi S, Maddirevula S, Salpietro V et al. Expanding the genetic heterogeneity of intellectual disability. *Hum Genet.* 2017; **136:** 1419-1429.

57. Appenzeller S BR, Barisic N, Baulac S, Caglayan H, Craiu D, De Jonghe P, Depienne C, Dimova P, Djémié T, Gormley P, Guerrini R, Helbig I, Hjalgrim H, Hoffman-Zacharska D, Jähn J, Klein KM, Koeleman B, Komarek V, Krause R, Kuhlenbäumer G, Leguern E, Lehesjoki AE, Lemke JR, Lerche H, Linnankivi T, Marini C, May P, Møller RS, Muhle H, Pal D, Palotie A, Pendziwiat M, Robbiano A, Roelens F, Rosenow F, Selmer K, Serratosa JM, Sisodiya S, Stephani U, Sterbova K, Striano P, Suls A, Talvik T, von Spiczak S, Weber Y, Weckhuysen S, Zara F, Abou-Khalil B, Alldredge BK, Andermann E, Andermann F, Amron D, Bautista JF, Berkovic SF, Bluvstein J, Boro A, Cascino G, Consalvo D, Crumrine P, Devinsky O, Dlugos D, Epstein MP, Fiol M, Fountain NB, French J, Friedman D, Geller EB, Glauser T, Glynn S, Haas K, Haut SR, Hayward J, Helmers SL, Joshi S, Kanner A, Kirsch HE, Knowlton RC, Kossoff EH, Kuperman R, Kuzniecky R, Lowenstein DH, McGuire SM, Motika PV, Novotny EJ, Ottman R, Paolicchi JM, Parent J, Park K, Poduri A, Sadleir L, Scheffer IE, Shellhaas RA, Sherr E, Shih JJ, Singh R, Sirven J, Smith MC, Sullivan J, Thio LL, Venkat A, Vining EP, Von Allmen GK, Weisenberg JL, Widdess-Walsh P, Winawer MR, Allen AS, Berkovic SF, Cossette P, Delanty N, Dlugos D, Eichler EE, Epstein MP, Glauser T, Goldstein DB, Han Y, Heinzen EL, Johnson MR, Kuzniecky R, Lowenstein DH, Marson AG, Mefford HC, Nieh SE, O'Brien TJ, Ottman R, Petrou S, Petrovski S, Poduri A, Ruzzo EK, Scheffer IE, Sherr E. De novo mutations in synaptic transmission genes including DNM1 cause epileptic encephalopathies. *Am J Hum Genet.* 2014; **95:** 360-370.

58. Anazi S, Maddirevula S, Faqeih E et al. Clinical genomics expands the morbid genome of intellectual disability and offers a high diagnostic yield. *Mol Psychiatry.* 2017; **22:** 615-624.

59. Nahorski MS, Maddirevula S, Ishimura R et al. Biallelic UFM1 and UFC1 mutations expand the essential role of ufmylation in brain development. *Brain.* 2018; **141:** 1934-1945.

60. Colin E, Daniel J, Ziegler A et al. Biallelic Variants in UBA5 Reveal that Disruption of the UFM1 Cascade Can Result in Early-Onset Encephalopathy. *Am J Hum Genet.* 2016; **99:** 695-703.

61. Arnadottir GA, Jensson BO, Marelsson SE et al. Compound heterozygous mutations in UBA5 causing early-onset epileptic encephalopathy in two sisters. *BMC Med Genet.* 2017; **18:** 103.

62. Muona M, Ishimura R, Laari A et al. Biallelic Variants in UBA5 Link Dysfunctional UFM1 Ubiquitin-like Modifier Pathway to Severe Infantile-Onset Encephalopathy. *Am J Hum Genet.* 2016; **99:** 683-694.

63. Low KJ, Baptista J, Babiker M et al. Hemizygous UBA5 missense mutation unmasks recessive disorder in a patient with infantile-onset encephalopathy, acquired microcephaly, small cerebellum, movement disorder and severe neurodevelopmental delay. *Eur J Med Genet.* 2018.

64. Zhong F, Savage SA, Shkreli M et al. Disruption of telomerase trafficking by TCAB1 mutation causes dyskeratosis congenita. *Genes Dev.* 2011; **25:** 11-16.

65. Batista LF, Pech MF, Zhong FL et al. Telomere shortening and loss of self-renewal in dyskeratosis congenita induced pluripotent stem cells. *Nature.* 2011; **474:** 399-402.

66. Walne AJ, Vulliamy T, Kirwan M, Plagnol V, Dokal I. Constitutional mutations in RTEL1 cause severe dyskeratosis congenita. *Am J Hum Genet.* 2013; **92:** 448-453.

67. Frescas D, de Lange T. A TIN2 dyskeratosis congenita mutation causes telomerase-independent telomere shortening in mice. *Genes Dev.* 2014; **28:** 153-166.

68. Kumar P, Henikoff S, Ng PC. Predicting the effects of coding non-synonymous variants on protein function using the SIFT algorithm. *Nat Protoc.* 2009; **4:** 1073-1081.

69. Schwarz JM, Cooper DN, Schuelke M, Seelow D. MutationTaster2: mutation prediction for the deep-sequencing age. *Nat Methods.* 2014; **11:** 361-362.

70. Adzhubei I, Jordan DM, Sunyaev SR. Predicting functional effect of human missense mutations using PolyPhen-2. *Curr Protoc Hum Genet.* 2013; **Chapter 7:** Unit7 20.

71. Richards S, Aziz N, Bale S et al. Standards and guidelines for the interpretation of sequence variants: a joint consensus recommendation of the American College of Medical Genetics and Genomics and the Association for Molecular Pathology. *Genet Med.* 2015; **17:** 405-424.

72. Dork T, Bendix R, Bremer M et al. Spectrum of ATM gene mutations in a hospital-based series of unselected breast cancer patients. *Cancer Res.* 2001; **61:** 7608-7615.

73. Rossetti R, Di Pasquale E, Marozzi A et al. BMP15 mutations associated with primary ovarian insufficiency cause a defective production of bioactive protein. *Hum Mutat.* 2009; **30:** 804-810.

74. Calvo SE, Tucker EJ, Compton AG et al. High-throughput, pooled sequencing identifies mutations in NUBPL and FOXRED1 in human complex I deficiency. *Nat Genet.* 2010; **42:** 851-858.

75. Pippucci T, Licchetta L, Baldassari S et al. Epilepsy with auditory features: A heterogeneous clinico-molecular disease. *Neurol Genet.* 2015; **1:** e5.

76. Heron SE, Khosravani H, Varela D et al. Extended spectrum of idiopathic generalized epilepsies associated with CACNA1H functional variants. *Ann Neurol.* 2007; **62:** 560-568.

77. Peddareddygari LR, Grewal AS, Grewal RP. Focal seizures in a patient with myotonic disorder type 2 co-segregating with a chloride voltage-gated channel 1 gene mutation: a case report. *J Med Case Rep.* 2016; **10:** 167.

78. Sun C, Tranebjaerg L, Torbergsen T, Holmgren G, Van Ghelue M. Spectrum of CLCN1 mutations in patients with myotonia congenita in Northern Scandinavia. *Eur J Hum Genet.* 2001; **9:** 903-909.

79. Tincheva S, Georgieva B, Todorov T et al. Myotonia congenita type Becker in Bulgaria: First genetically proven cases and mutation screening of two presumable endemic regions. *Neuromuscul Disord.* 2016; **26:** 675-680.

80. Saint-Martin C, Gauvain G, Teodorescu G et al. Two novel CLCN2 mutations accelerating chloride channel deactivation are associated with idiopathic generalized epilepsy. *Hum Mutat.* 2009; **30:** 397-405.

81. Lagier-Tourenne C, Tazir M, Lopez LC et al. ADCK3, an ancestral kinase, is mutated in a form of recessive ataxia associated with coenzyme Q10 deficiency. *Am J Hum Genet.* 2008; **82:** 661-672.

82. Giunta C, Nuytinck L, Raghunath M, Hausser I, De Paepe A, Steinmann B. Homozygous Gly530Ser substitution in COL5A1 causes mild classical Ehlers-Danlos syndrome. *Am J Med Genet.* 2002; **109:** 284-290.

83. Levesque S, Auray-Blais C, Gravel E et al. Diagnosis of late-onset Pompe disease and other muscle disorders by next-generation sequencing. *Orphanet J Rare Dis.* 2016; **11:** 8.

84. Kim S, Westphal V, Srikrishna G et al. Dolichol phosphate mannose synthase (DPM1) mutations define congenital disorder of glycosylation Ie (CDG-Ie). *J Clin Invest.* 2000; **105:** 191-198.

85. Xiong HY, Alipanahi B, Lee LJ et al. RNA splicing. The human splicing code reveals new insights into the genetic determinants of disease. *Science.* 2015; **347:** 1254806.

86. Takasaki N, Tachibana K, Ogasawara S et al. A heterozygous mutation of GALNTL5 affects male infertility with impairment of sperm motility. *Proc Natl Acad Sci U S A.* 2014; **111:** 1120-1125.

87. Mahdieh N, Rabbani B. Statistical study of 35delG mutation of GJB2 gene: a meta-analysis of carrier frequency. *Int J Audiol.* 2009; **48:** 363-370.

88. White TW, Deans MR, Kelsell DP, Paul DL. Connexin mutations in deafness. *Nature.* 1998; **394:** 630-631.

89. Pollak A, Skorka A, Mueller-Malesinska M et al. M34T and V37I mutations in GJB2 associated hearing impairment: evidence for pathogenicity and reduced penetrance. *Am J Med Genet A.* 2007; **143A:** 2534-2543.

90. Liu JA, Lai FP, Gui HS et al. Identification of GLI Mutations in Patients With Hirschsprung Disease That Disrupt Enteric Nervous System Development in Mice. *Gastroenterology.* 2015; **149:** 1837-1848 e1835.

91. Neubauer BA, Waldegger S, Heinzinger J et al. KCNQ2 and KCNQ3 mutations contribute to different idiopathic epilepsy syndromes. *Neurology.* 2008; **71:** 177-183.

92. Miceli F, Soldovieri MV, Lugli L et al. Neutralization of a unique, negatively-charged residue in the voltage sensor of K V 7.2 subunits in a sporadic case of benign familial neonatal seizures. *Neurobiol Dis.* 2009; **34:** 501-510.

93. Gilling M, Rasmussen HB, Calloe K et al. Dysfunction of the Heteromeric KV7.3/KV7.5 Potassium Channel is Associated with Autism Spectrum Disorders. *Front Genet.* 2013; **4:** 54.

94. Lemke JR, Riesch E, Scheurenbrand T et al. Targeted next generation sequencing as a diagnostic tool in epileptic disorders. *Epilepsia.* 2012; **53:** 1387-1398.

95. Luxan G, Casanova JC, Martinez-Poveda B et al. Mutations in the NOTCH pathway regulator MIB1 cause left ventricular noncompaction cardiomyopathy. *Nat Med.* 2013; **19:** 193-201.

96. Ziganshin BA, Bailey AE, Coons C et al. Routine Genetic Testing for Thoracic Aortic Aneurysm and Dissection in a Clinical Setting. *Ann Thorac Surg.* 2015; **100:** 1604-1611.

97. de Ligt J, Willemsen MH, van Bon BW et al. Diagnostic exome sequencing in persons with severe intellectual disability. *N Engl J Med.* 2012; **367:** 1921-1929.

98. Yuen RK, Thiruvahindrapuram B, Merico D et al. Whole-genome sequencing of quartet families with autism spectrum disorder. *Nat Med.* 2015; **21:** 185-191.

99. Potluri P, Davila A, Ruiz-Pesini E et al. A novel NDUFA1 mutation leads to a progressive mitochondrial complex I-specific neurodegenerative disease. *Mol Genet Metab.* 2009; **96:** 189-195.

100. Piton A, Redin C, Mandel JL. XLID-causing mutations and associated genes challenged in light of data from large-scale human exome sequencing. *Am J Hum Genet.* 2013; **93:** 368-383.

101. Horvath A, Boikos S, Giatzakis C et al. A genome-wide scan identifies mutations in the gene encoding phosphodiesterase 11A4 (PDE11A) in individuals with adrenocortical hyperplasia. *Nat Genet.* 2006; **38:** 794-800.

102. Carney JA, Gaillard RC, Bertherat J, Stratakis CA. Familial micronodular adrenocortical disease, Cushing syndrome, and mutations of the gene encoding phosphodiesterase 11A4 (PDE11A). *Am J Surg Pathol.* 2010; **34:** 547-555.

103. Libe R, Horvath A, Vezzosi D et al. Frequent phosphodiesterase 11A gene (PDE11A) defects in patients with Carney complex (CNC) caused by PRKAR1A mutations: PDE11A may contribute to adrenal and testicular tumors in CNC as a modifier of the phenotype. *J Clin Endocrinol Metab.* 2011; **96:** E208-214.

104. Azevedo MF, Horvath A, Bornstein ER et al. Cyclic AMP and c-KIT signaling in familial testicular germ cell tumor predisposition. *J Clin Endocrinol Metab.* 2013; **98:** E1393-1400.

105. Gala MK, Mizukami Y, Le LP et al. Germline mutations in oncogene-induced senescence pathways are associated with multiple sessile serrated adenomas. *Gastroenterology.* 2014; **146:** 520-529.

106. Garcia-Gonzalez MA, Jones JG, Allen SK et al. Evaluating the clinical utility of a molecular genetic test for polycystic kidney disease. *Mol Genet Metab.* 2007; **92:** 160-167.

107. Liu B, Chen SC, Yang YM et al. Identification of novel PKD1 and PKD2 mutations in a Chinese population with autosomal dominant polycystic kidney disease. *Sci Rep.* 2015; **5:** 17468.

108. Lacombe A, Lee H, Zahed L et al. Disruption of POF1B binding to nonmuscle actin filaments is associated with premature ovarian failure. *Am J Hum Genet.* 2006; **79:** 113-119.

109. Bender HU, Almashanu S, Steel G et al. Functional consequences of PRODH missense mutations. *Am J Hum Genet.* 2005; **76:** 409-420.

110. Razzaque MA, Nishizawa T, Komoike Y et al. Germline gain-of-function mutations in RAF1 cause Noonan syndrome. *Nat Genet.* 2007; **39:** 1013-1017.

111. Patel N, Aldahmesh MA, Alkuraya H et al. Expanding the clinical, allelic, and locus heterogeneity of retinal dystrophies. *Genet Med.* 2016; **18:** 554-562.

112. Medeiros-Domingo A, Bhuiyan ZA, Tester DJ et al. The RYR2-encoded ryanodine receptor/calcium release channel in patients diagnosed previously with either catecholaminergic polymorphic ventricular tachycardia or genotype negative, exercise-induced long QT syndrome: a comprehensive open reading frame mutational analysis. *J Am Coll Cardiol.* 2009; **54:** 2065-2074.

113. Lehnart SE, Mongillo M, Bellinger A et al. Leaky Ca2+ release channel/ryanodine receptor 2 causes seizures and sudden cardiac death in mice. *J Clin Invest.* 2008; **118:** 2230-2245.

114. Dorschner MO, Amendola LM, Turner EH et al. Actionable, pathogenic incidental findings in 1,000 participants' exomes. *Am J Hum Genet.* 2013; **93:** 631-640.

115. Amendola LM, Dorschner MO, Robertson PD et al. Actionable exomic incidental findings in 6503 participants: challenges of variant classification. *Genome Res.* 2015; **25:** 305-315.

116. Maxwell KN, Hart SN, Vijai J et al. Evaluation of ACMG-Guideline-Based Variant Classification of Cancer Susceptibility and Non-Cancer-Associated Genes in Families Affected by Breast Cancer. *Am J Hum Genet.* 2016; **98:** 801-817.

117. Weiss LA, Escayg A, Kearney JA et al. Sodium channels SCN1A, SCN2A and SCN3A in familial autism. *Mol Psychiatry.* 2003; **8:** 186-194.

118. Escayg A, Heils A, MacDonald BT, Haug K, Sander T, Meisler MH. A novel SCN1A mutation associated with generalized epilepsy with febrile seizures plus--and prevalence of variants in patients with epilepsy. *Am J Hum Genet.* 2001; **68:** 866-873.

119. Orrico A, Galli L, Grosso S et al. Mutational analysis of the SCN1A, SCN1B and GABRG2 genes in 150 Italian patients with idiopathic childhood epilepsies. *Clin Genet.* 2009; **75:** 579-581.

120. Wang JW, Shi XY, Kurahashi H et al. Prevalence of SCN1A mutations in children with suspected Dravet syndrome and intractable childhood epilepsy. *Epilepsy Res.* 2012; **102:** 195-200.

121. Usluer S, Salar S, Arslan M et al. SCN1A gene sequencing in 46 Turkish epilepsy patients disclosed 12 novel mutations. *Seizure.* 2016; **39:** 34-43.

122. Lal D, Reinthaler EM, Dejanovic B et al. Evaluation of Presumably Disease Causing SCN1A Variants in a Cohort of Common Epilepsy Syndromes. *PLoS One.* 2016; **11:** e0150426.

123. Lim ET, Raychaudhuri S, Sanders SJ et al. Rare complete knockouts in humans: population distribution and significant role in autism spectrum disorders. *Neuron.* 2013; **77:** 235-242.

124. Maillard-Lefebvre H, Charlanne H, Hatron PY, Dode C, Hachulla E. Recurrent limb and facial oedema with R92Q mutation: a non-febrile late-onset tumour necrosis factor receptor-associated periodic syndrome (TRAPS)? *Presse Med.* 2013; **42:** 905-906.

125. Hoogeveen-Westerveld M, Wentink M, van den Heuvel D et al. Functional assessment of variants in the TSC1 and TSC2 genes identified in individuals with Tuberous Sclerosis Complex. *Hum Mutat.* 2011; **32:** 424-435.

126. Itoh-Satoh M, Hayashi T, Nishi H et al. Titin mutations as the molecular basis for dilated cardiomyopathy. *Biochem Biophys Res Commun.* 2002; **291:** 385-393.

127. Herman DS, Lam L, Taylor MR et al. Truncations of titin causing dilated cardiomyopathy. *N Engl J Med.* 2012; **366:** 619-628.

128. Chauveau C, Bonnemann CG, Julien C et al. Recessive TTN truncating mutations define novel forms of core myopathy with heart disease. *Hum Mol Genet.* 2014; **23:** 980-991.

129. Hinson JT, Chopra A, Nafissi N et al. HEART DISEASE. Titin mutations in iPS cells define sarcomere insufficiency as a cause of dilated cardiomyopathy. *Science.* 2015; **349:** 982-986.

130. Roberts AM, Ware JS, Herman DS et al. Integrated allelic, transcriptional, and phenomic dissection of the cardiac effects of titin truncations in health and disease. *Sci Transl Med.* 2015; **7:** 270ra276.

131. Paduch DA, Mielnik A, Schlegel PN. Novel mutations in testis-specific ubiquitin protease 26 gene may cause male infertility and hypogonadism. *Reprod Biomed Online.* 2005; **10:** 747-754.

132. Yang J, Wang SK, Choi M et al. Taurodontism, variations in tooth number, and misshapened crowns in Wnt10a null mice and human kindreds. *Mol Genet Genomic Med.* 2015; **3:** 40-58.

133. Zollo M, Ahmed M, Ferrucci V et al. PRUNE is crucial for normal brain development and mutated in microcephaly with neurodevelopmental impairment. *Brain.* 2017; **140:** 940-952.

134. Costain G, Shugar A, Krishnan P, Mahmutoglu S, Laughlin S, Kannu P. Homozygous mutation in PRUNE1 in an Oji-Cree male with a complex neurological phenotype. *Am J Med Genet A.* 2017; **173:** 740-743.

135. Karakaya M, Yilmaz S, Storbeck M et al. PRUNE1: a disease-causing gene for secondary microcephaly. *Brain.* 2017; **140:** e61.

136. Kircher M, Witten DM, Jain P, O'Roak BJ, Cooper GM, Shendure J. A general framework for estimating the relative pathogenicity of human genetic variants. *Nat Genet.* 2014; **46:** 310-315.

137. Landrum MJ, Lee JM, Benson M et al. ClinVar: public archive of interpretations of clinically relevant variants. *Nucleic Acids Res.* 2016; **44:** D862-868.

138. Forbes SA, Beare D, Gunasekaran P et al. COSMIC: exploring the world's knowledge of somatic mutations in human cancer. *Nucleic Acids Res.* 2015; **43:** D805-811.

139. Sherry ST, Ward MH, Kholodov M et al. dbSNP: the NCBI database of genetic variation. *Nucleic Acids Res.* 2001; **29:** 308-311.

140. Firth HV, Richards SM, Bevan AP et al. DECIPHER: Database of Chromosomal Imbalance and Phenotype in Humans Using Ensembl Resources. *Am J Hum Genet.* 2009; **84:** 524-533.

141. MacDonald JR, Ziman R, Yuen RK, Feuk L, Scherer SW. The Database of Genomic Variants: a curated collection of structural variation in the human genome. *Nucleic Acids Res.* 2014; **42:** D986-992.

142. Lek M, Karczewski KJ, Minikel EV et al. Analysis of protein-coding genetic variation in 60,706 humans. *Nature.* 2016; **536:** 285-291.

143. Gene Ontology Consortium: going forward. *Nucleic Acids Res.* 2015; **43:** D1049-1056.

144. Stenson PD, Ball EV, Mort M et al. Human Gene Mutation Database (HGMD): 2003 update. *Hum Mutat.* 2003; **21:** 577-581.

145. Wishart DS, Jewison T, Guo AC et al. HMDB 3.0--The Human Metabolome Database in 2013. *Nucleic Acids Res.* 2013; **41:** D801-807.

146. Kanehisa M, Furumichi M, Tanabe M, Sato Y, Morishima K. KEGG: new perspectives on genomes, pathways, diseases and drugs. *Nucleic Acids Res.* 2017; **45:** D353-D361.

147. Fokkema IF, Taschner PE, Schaafsma GC, Celli J, Laros JF, den Dunnen JT. LOVD v.2.0: the next generation in gene variant databases. *Hum Mutat.* 2011; **32:** 557-563.

148. Caspi R, Altman T, Billington R et al. The MetaCyc database of metabolic pathways and enzymes and the BioCyc collection of Pathway/Genome Databases. *Nucleic Acids Res.* 2014; **42:** D459-471.

149. Smith CA, O'Maille G, Want EJ et al. METLIN: a metabolite mass spectral database. *Ther Drug Monit.* 2005; **27:** 747-751.

150. Piirila H, Valiaho J, Vihinen M. Immunodeficiency mutation databases (IDbases). *Hum Mutat.* 2006; **27:** 1200-1208.

151. Berman HM, Westbrook J, Feng Z et al. The Protein Data Bank. *Nucleic Acids Res.* 2000; **28:** 235-242.

152. Hornbeck PV, Zhang B, Murray B, Kornhauser JM, Latham V, Skrzypek E. PhosphoSitePlus, 2014: mutations, PTMs and recalibrations. *Nucleic Acids Res.* 2015; **43:** D512-520.

153. Sigrist CJ, Cerutti L, de Castro E et al. PROSITE, a protein domain database for functional characterization and annotation. *Nucleic Acids Res.* 2010; **38:** D161-166.

154. O'Leary NA, Wright MW, Brister JR et al. Reference sequence (RefSeq) database at NCBI: current status, taxonomic expansion, and functional annotation. *Nucleic Acids Res.* 2016; **44:** D733-745.

155. Basu SN, Kollu R, Banerjee-Basu S. AutDB: a gene reference resource for autism research. *Nucleic Acids Res.* 2009; **37:** D832-836.

156. Kochinke K, Zweier C, Nijhof B et al. Systematic Phenomics Analysis Deconvolutes Genes Mutated in Intellectual Disability into Biologically Coherent Modules. *Am J Hum Genet.* 2016; **98:** 149-164.

157. Krogh A, Larsson B, von Heijne G, Sonnhammer EL. Predicting transmembrane protein topology with a hidden Markov model: application to complete genomes. *J Mol Biol.* 2001; **305:** 567-580.

158. Kent WJ, Sugnet CW, Furey TS et al. The human genome browser at UCSC. *Genome Res.* 2002; **12:** 996-1006.

159. UniProt: the universal protein knowledgebase. *Nucleic Acids Res.* 2017; **45:** D158-D169.
